# Developing Indicators of Nutrient Pollution in Streams Using 16S rRNA Gene Metabarcoding of Periphyton-Associated Bacteria

**DOI:** 10.3390/w14152361

**Published:** 2022-07-30

**Authors:** Erik M. Pilgrim, Nathan J. Smucker, Huiyun Wu, John Martinson, Christopher T. Nietch, Marirosa Molina, John A. Darling, Brent R. Johnson

**Affiliations:** 1 United States Environmental Protection Agency, Office of Research and Development, Cincinnati, OH 45268, USA; 2 School of Public Health & Tropical Medicine, Tulane University, New Orleans, LA 70112, USA; 3 United States Environmental Protection Agency, Office of Research and Development, Research Triangle Park, NC 27711, USA

**Keywords:** phosphorus, nitrogen, agriculture, periphyton, biomonitoring, bioassessment, 16S, gradient forest, boosted regression trees, threshold indicator taxa analysis, TITAN

## Abstract

Indicators based on nutrient-biota relationships in streams can inform water quality restoration and protection programs. Bacterial assemblages could be particularly useful indicators of nutrient effects because they are species-rich, important contributors to ecosystem processes in streams, and responsive to rapidly changing conditions. Here, we sampled 25 streams weekly (12–14 times each) and used 16S rRNA gene metabarcoding of periphyton-associated bacteria to quantify the effects of total phosphorus (TP) and total nitrogen (TN). Threshold indicator taxa analysis identified assemblage-level changes and amplicon sequence variants (ASVs) that increased or decreased with increasing TP and TN concentrations (i.e., low P, high P, low N, and high N ASVs). Boosted regression trees confirmed that relative abundances of gene sequence reads for these four indicator groups were associated with nutrient concentrations. Gradient forest analysis complemented these results by using multiple predictors and random forest models for each ASV to identify portions of TP and TN gradients at which the greatest changes in assemblage structure occurred. Synthesized statistical results showed bacterial assemblage structure began changing at 24 μg TP/L with the greatest changes occurring from 110 to 195 μg/L. Changes in the bacterial assemblages associated with TN gradually occurred from 275 to 855 μg/L. Taxonomic and phylogenetic analyses showed that low nutrient ASVs were commonly Firmicutes, Verrucomicrobiota, Flavobacteriales, and Caulobacterales, Pseudomonadales, and Rhodobacterales of Proteobacteria, whereas other groups, such as Chitinophagales of Bacteroidota, and Burkholderiales, Rhizobiales, Sphingomonadales, and Steroidobacterales of Proteobacteria comprised the high nutrient ASVs. Overall, the responses of bacterial ASV indicators in this study highlight the utility of metabarcoding periphyton-associated bacteria for quantifying biotic responses to nutrient inputs in streams.

## 1. Introduction

Nutrient pollution of freshwater ecosystems is a persistent and growing concern around the world [1-3], often being the most common stressor affecting streams [4-7]. Inputs of nitrogen or phosphorus create environmental stress by causing changes in the primary producer communities, reductions in biodiversity, alterations to food webs, hypoxia, and harmful algal blooms [8-12]. These negative impacts are expected to continue to increase as human populations grow, requiring more food production and watershed development, leading to further stress and alteration of freshwater and coastal ecosystems [13-15]. The development of methods for measuring ecosystem responses to nutrient pollution is therefore crucial for understanding how to mitigate or prevent ecological damage. Decision makers, regulatory agencies, and other end users need the best tools possible to measure, address, and alleviate the negative effects of nutrient pollution.

In aquatic environments, the benthic bacterial assemblage is part of the periphyton (or stream biofilm community) that is critical to biogeochemical processing. Stream biogeochemistry is driven by water quality and abiotic-biotic interactions within the periphyton matrix [16,17]. Bacteria are biogeochemical workhorses, responsible for important processes such as nitrification, denitrification, metabolism of pollutants, and producing hydrolytic enzymes that promote the turnover of organic carbon and nutrients [18-21]. These bacteria-mediated processes, coupled with their abilities to quickly replicate and increase biomass in response to changes in environmental conditions [22,23], offer a basal response to changing nutrient conditions in streams [24]. As a result, the responses of the bacterial component of stream periphyton to nitrogen and phosphorus concentrations can be used as indicators that provide context for understanding nutrient effects on streams [25,26]. Tools that help elucidate periphytic microbial responses to nutrient pollution therefore can provide valuable information for understanding and managing the effects of nitrogen and phosphorus pollution.

Aquatic bacterial assemblages, however, can be challenging to characterize. Many microbes are impossible to culture and study in the laboratory, including the bulk of taxa that would be of interest in ecological and environmental studies [27,28]. For this reason, molecular genetic techniques like DNA metabarcoding have been employed to sample and analyze a variety of microbial communities [17,29]. DNA metabarcoding has become a valuable tool for gathering extensive amounts of microbial community data that would have been very costly or nearly impossible a few years ago [30-34]. Recent studies in freshwater environments have shown bacterial assemblage responses to nutrients [26,35,36], flow and fine sediment [37], temperature and pH [36], and conductivity [35].

In this study of streams in southwestern Ohio, USA, we applied DNA metabarcoding to the bacterial assemblages associated with periphyton. Using samples collected weekly from sites representing a broad range of nitrogen and phosphorus conditions within a large watershed, we analyzed bacterial taxa, defined as amplicon sequence variants (ASVs), and the associated water chemistry data with threshold indicator taxa analysis (TITAN; [38]), boosted regression trees (BRT; [39,40]), and gradient forests (GF; [41]) to determine how bacterial assemblages and individual taxa respond to different nutrient concentrations. We also used phylogenetic analysis to show the distribution of high and low nutrient indicator taxa among the major bacterial groups.

## 2. Materials and Methods

### 2.1. Site Selection and Sampling

**Figure 1.**
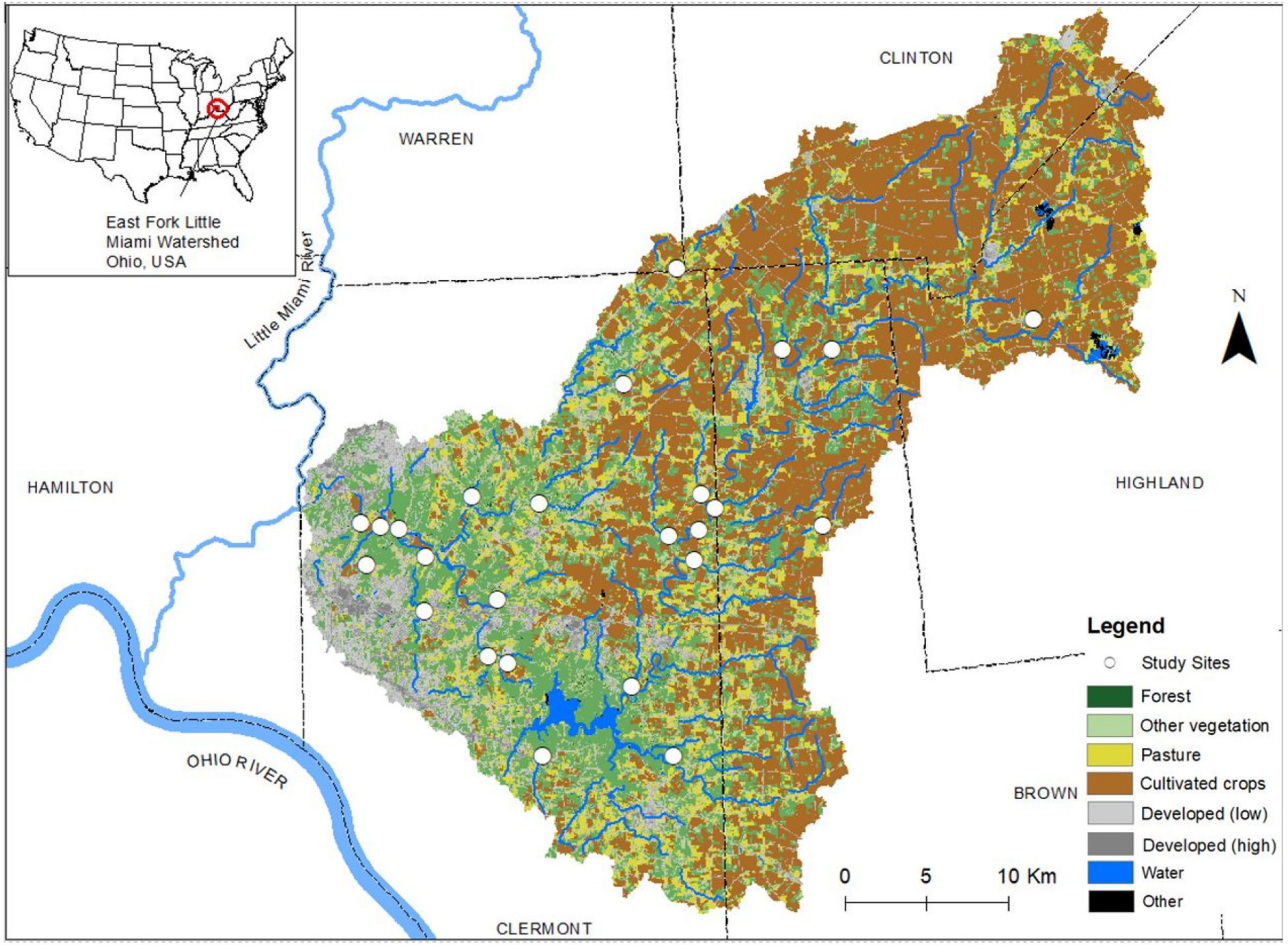
Map of collection sites in the East Fork of the Little Miami River Watershed.

Twenty-five sites in second- to third-order wadeable streams within the East Fork of the Little Miami River watershed of southwestern Ohio (USA) (Figure 1, Supplementary Table S1) were chosen based on historical monitoring data and site characterizations to comprise a gradient of both nitrogen and phosphorus concentrations across sites while minimizing possible confounding effects of non-nutrient factors. The watershed experiences a temperate seasonal climate and the 25 stream sites covered a range of watershed sizes (15.8–82.3 km^2^, median 37.6 km^2^, mean 40.3 km^2^ ± 16.4 km^2^), percent agriculture coverage (0–88%, median 53%, mean 48% ± 29%), percent forest coverage (9–59%, median 32%, mean 33% ± 14%), and percent urban coverage (0–69%, median 8%, mean 19% ± 20%).

Sites were sampled weekly from early July through September 2016 with another 1–2 samples taken in October. Each site had 13–14 periphyton samples (*n* = 342 among sites) and 10–12 nutrient samples collected (TP *n* = 281, TN *n* = 280 among sites). At each site, water was collected in 1-L acid-washed polypropylene bottles for nutrient analyses and periphyton was scraped and composited from five rocks from five equally spaced transects over a 75-m stream reach collected with a firm-bristled brush on a cordless drill using a 6.7 cm^2^ plastic guide. Periphyton samples were stored frozen at −80 °C until thawed for DNA analysis. Water samples for total phosphorus (TP) and total nitrogen (TN) were stored in the dark at 4 °C until analyzed within 24 h or were frozen at −20 °C for analysis within 14 days using a QuikChem 8500 nutrient autoanalyzer system (Lachat Instruments, Milwaukee, WI, USA). Acid persulfate wet digestions were conducted prior to using the molybdate and antimony potassium tartrate reaction and ascorbic acid reduction method to measure TP [42,43]. Total nitrogen was measured using an alkaline wet oxidation persulfate method prior to cadmium reduction [44,45].

### 2.2. DNA Metabarcoding Workflow

Upon slow thawing in a refrigerator, samples were immediately filtered through sterile polycarbonate (0.8 μm) filters. These filters were chosen to capture the algal and cyanobacterial portion of the periphyton (see [46]) but also were expected to capture most of the bacteria present in the periphyton. We recognize, however, that some loss of the smallest members (<0.8 μm) of the bacterial community could have occurred. Material collected on the filter was scraped and subsampled (~50 mg) for DNA extraction. This periphyton subsample was then ground in a 1.5-mL microcentrifuge tube with a pestle after treatment with liquid nitrogen, and the ground sample was extracted with the Qiagen DNeasy PowerLyzer PowerSoil kit (Germantown, MD, USA) according to the manufacturer’s protocol, with the addition of initial tissue digestion with proteinase K for at least 2 h at 56°C. Extraction blanks were processed approximately every 100 samples. Isolated DNA was quantified with PicoGreen on a Bio-Tek Synergy HT1 Microplate Reader (Winooski, VT, USA) and then normalized to 10 ng/ μL prior to amplification by polymerase chain reaction (PCR).

PCR was used on the normalized DNA samples to amplify a segment of the 16S ribosomal gene covering parts of variable regions V3 to V5, targeting bacteria. This genetic locus is commonly used in metabarcoding studies of microbial communities [33,47,48]. PCRs, including negative controls, were run at 20 μL final volumes that included 2 μL of Qiagen 10X PCR buffer (with MgCl_2_), 0.6 μL of 25 mM MgCl_2_, 1 μL each of the primers (forward, 515F: GTGCCAGCMGCCGCGGTAA; reverse, 806R: GGACTACHVGGGTWTCTAAT), 0.4 μL of 10 mM dNTPs, 4 μL of 1X BSA, 8.9 μL sterile water, 0.1 μL Taq polymerase (Qiagen), and 2 μL of normalized template DNA. The reaction program for the thermal cycler was 94 °C for 2 m 30 s, then 35 cycles of 94 ° C for 30 s, 50 ° C for 60 s, and 72 °C for 60 s and a final 10m extension at 72 °C. Each template was amplified in triplicate and those replicates were then pooled and cleaned (Qiagen Qiaquick 96 PCR Purification Kit). Negative controls and extraction blanks were not included beyond this step as amplicon bands were not present.

Cleaned PCR amplicons went through a second round of PCR (dual indexing) to add adapters and indexes for runs on an Illumina MiSeq (San Diego, CA, USA). The original PCR primers had upstream (5′) adapter sequences (forward: ACACTGACGACATGGTTCTACA; reverse: TACGGTAGCAGAGACTTGGTCT) to provide priming sites for the dual index PCR primers. This index PCR had a reaction program of eight cycles of 95 °C for 30 s, 55 ° C for 30 s, and 72 ° C for 30 s, with a final extension of 72 °C for 5 min. These index PCR amplicons were cleaned with the AMPure XP Kit (Beckman Coulter Life Science, Indianapolis, IN, USA). Cleaned amplicons were quantified with PicoGreen (as above), normalized in Qiagen EB buffer, and then all index PCR plates were pooled into a single solution using 3 μL of each amplicon. This pooled sample was then sequenced on an Illumina MiSeq using a 500-cycle (v2) MiSeq Sequencing Kit (paired-end, 250 base pairs) according to the manufacturer’s protocols.

### 2.3. Bioinformatics

QIIME2 was used for bioinformatic metagenomics analysis (version 2020; https://qiime2.org, accessed on 3 Jun 2020) [49]. Sample metadata were created by following the template for the project. Raw reads were imported into QIIME2 followed the “Casava 1.8 paired-end demultiplexed fastq” format. Quality filtration, denoising, merging of paired reads, and chimera removal were carried out by using the DADA2 pipeline [50] implemented in the QIIME2 environment. Filtration parameters were set as: 25 bases trimming at the left side of forward and reverse reads, and truncation of forward and reverse reads up to 250 bases. Raw DNA reads were not clustered into operational taxonomic units (OTUs), and instead kept as amplicon sequence variants (ASVs) to avoid reducing the diversity of potential indicator taxa and because OTUs from clustering are difficult to compare with other data sets. ASVs with only a single DNA sequence, however, were excluded. Although ASVs for this genetic locus could be considered analogous to species, we maintain a more conservative stance and only refer to them as ASVs. Taxonomic assignments were made with SILVA (Supplementary File) [51].

### 2.4. Statistical Analyses

Our objectives were to characterize bacterial assemblage–environment relationships and to identify indicator taxa responsive to TP and TN concentrations. Quantifying and summarizing these assemblage and taxa changes can help identify possible management targets for TP or TN concentrations. For each statistical analysis, we used data from all sampling events for each site because this incorporated the variability in nutrient concentrations over time and provides a fuller representation of nutrient conditions at sites, likely leading to more robust indicator development. All statistical analyses used relative abundances of gene sequence reads for each ASV in a sample.

We used nonmetric multidimensional scaling (NMDS) to visualize changes in bacterial assemblage structure among samples and Spearman correlations to examine relationships of axis scores with TN, TP, watershed land cover, and bacterial metrics (see below). For multivariate statistics, we originally included taxa with >1% relative abundance in > 1% of samples, which reduces the effects of noise introduced by including many rare taxa [52] or DNA sequencing errors such as tag jumping, and is a reasonable and conservative criterion [52-56]. This criterion, however, left us with using <60% of the total gene sequence reads among all samples. So, to compromise between excluding a large proportion of assemblages and including an excessive number of rare ASVs, we included ASVs in the NMDS that first were observed in >5 samples and then those that comprised 75% of the total gene sequence reads when ordered by decreasing average relative abundance (429 of 18,406 ASVs). The requirement for observations in >5 samples also meets a recommended criterion for inclusion in threshold indicator taxa analysis (TITAN). For the NMDS, relative abundances of ASV gene sequence reads were square-root transformed, the Bray–Curtis dissimilarity coefficient was used, and the ordination was rotated to maximize variation along the first axis. We used the vegan package and the metaMDS function [57] in R v. 4.0.3 [58] to conduct the NMDS.

We used TITAN to characterize assemblage level changes and to identify ASVs that either increased or decreased with increasing TP or TN concentrations [38]. TITAN uses bootstrapping and indicator species analysis to identify the point along an environmental gradient at which each ASV has the greatest change in its frequency and relative abundance. Response magnitudes are standardized to z-scores to facilitate cross-taxa comparisons. Bootstrapping also identifies ASVs with consistently strong responses that increase or decrease in >95% of bootstrapped replicates. For ease of communication, we subsequently refer to ASVs that decrease and ASVs that increase with greater TP or TN concentrations as low P or N taxa and high P or N taxa, respectively. The distribution of bootstrapped ASV change points and sum z-scores of all indicator taxa at each observed TP or TN concentration (i.e., partition) shows how taxa and overall assemblages change along the TP or TN gradient [59]. We used R v. 3.4.3 [56] and the ‘TITAN2’ package [60] to conduct TITAN for TP and TN gradients with 1000 bootstraps. We included all ASVs used in the NMDS for consistency among statistical analyses.

For each sample, we summed the relative abundances of ASVs in each indicator group—low P, high P, low N, and high N bacteria—which could be used as metrics interpreted in a manner like that of traditional biomonitoring based on other organisms in streams (e.g., [61-63]). Spearman correlations were used to describe relationships of bacterial metrics with nutrients and watershed land cover. We also used boosted regression trees to examine relationships between bacterial metrics and multiple predictor variables. Boosted regression tree analysis is a machine learning method that combines results from thousands of regression trees into a model with high predictive performance [39,40]. This analysis can handle correlated predictors, determine the relative importance of predictors, model a variety of responses, and generate partial dependence plots showing the effects of each predictor while controlling for the mean effects of other variables in the model. The partial dependence plots were used to describe nutrient-bacteria relationships. We used a 10-fold cross-validation procedure, which used all data for training and validation steps, to evaluate model performance and to determine mean correlations between fitted and raw values and mean predictive performance of models reported as the percentage of null deviance explained. We used TP, TN, and conductivity as predictors and each of the four bacterial metrics as response variables. Using R v. 3.4.3 [58] and the ‘dismo’ package [64], we set tree complexity = 2, learning rate = 0.001, and bag fraction = 0.5, which created more than the recommended minimum of 1000 trees for each model [65].

Lastly, we used gradient forest analysis to characterize relationships between multi-taxa assemblages, using ASV relative abundances, and multiple environmental predictor variables in one statistical analysis. This analysis provides complementary results to TITAN, which uses multi-taxa responses to a single predictor based on relative abundance and occurrence data, and to boosted regression trees, which model single variable responses to multiple predictors. Gradient forest analysis combines results from random forest models for each ASV with R^2^ values > 0 into a measure of overall change in assemblage structure [41]. For each ASV, the split values and their importance (i.e., variation explained by the partitioning) are collated using the R package ‘extendedForest’ and then used to compute multi-species and assemblage change functions along each environmental gradient and to produce visualizations of results with the R package ‘gradientForest’ [41]. Split density plots, which incorporate the weighted importance of ASV splits and are standardized as a ratio to the density of data, show where the greatest rates of assemblage change occur along environmental gradients (ratios >1). We interpreted peaks as the predictor values at which greatest change occurred, and the increase and decrease in splits surrounding these peaks as denoting unique regions of change along the gradient when ratios were > 1. Random permutations are used to determine the conditional importance of each predictor variable by measuring subsequent degradation in model performance.

### 2.5. Phylogenetic Analysis

Sequence data for the 429 ASVs used in the indicator analyses (see above) were aligned with Clustal W using default settings in MEGA-X [66]. To help assess the potential relatedness of ASVs marked as nutrient indicators, a maximum likelihood (ML) tree was generated in MEGA-X using the generalized time-reversible model with gamma-distributed rates and invariant sites (GTR + I + G). This more complex model of evolution was chosen for the ML analysis given that the ASVs were spread across multiple bacterial phyla. The ML tree was drawn and annotated in iTOL v5 [67].

## 3. Results

### 3.1. Descriptive Summary of Data

TP values for water collected at the stream sites ranged from 18 to 889 μg/L (N = 280, median 171 μg/L, mean ± standard deviation = 220 ± 179 μg/L). TN values for water collected at the stream sites ranged from 76 to 6560 μg/L (N = 281, median 621 μg/L, mean ± standard deviation = 778 ± 615 μg/L). The gradients of TP and TN concentrations were continuous and well represented with no gaps or overrepresentation of certain values (see [46] for further details). Historical conductivity values for water collected at the stream sites ranged from 373 to 789 μS/cm (N = 25, median 544 μS/cm, mean ± standard deviation = 561 ± 112 μS/cm).

The MiSeq run of the 342 periphyton samples generated nearly 6 million sequence reads (mean: 17,538 reads/sample; median: 17,179 reads/sample; minimum: 3258 reads; maximum: 36,102 reads) of appropriate length. Analysis of those reads found 18,406 ASVs with 2 or more reads. The top 75% of ASVs (based on reads per ASV) used for the NMDS and indicator analyses comprised 429 ASVs with approximately 4.5 million sequence reads.

**Figure 2.**
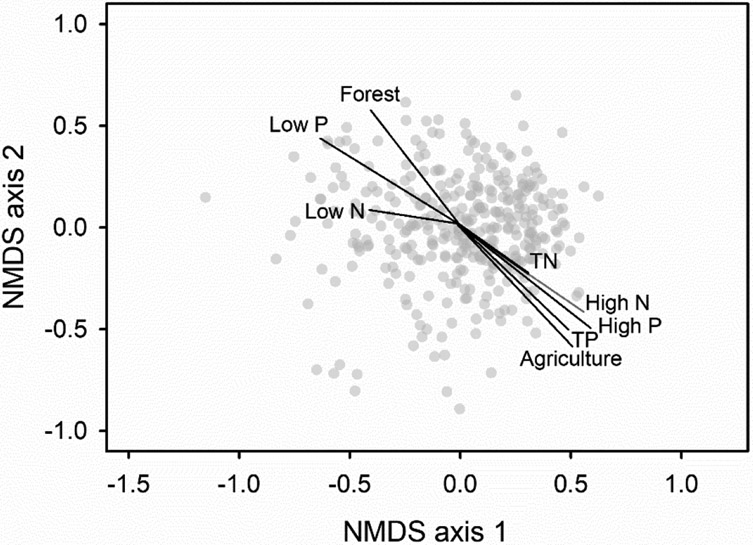
Nonmetric multidimensional scaling ordination showing Spearman correlations of axis scores with total phosphorus (TP), total nitrogen (TN), watershed percent forest, watershed percent agriculture, and relative abundances of low P, low N, high P, and high N bacterial ASVs. Vectors are scaled to span the range of possible correlation coefficients (−1 to 1) along NMDS axes.

Nonmetric multidimensional scaling showed that changes in bacterial assemblage structure among samples were associated with percent forest and percent agriculture in watersheds and with TP and TN concentrations (Figure 2). More forested land cover, less agriculture, and lower TP and TN concentrations were associated with decreasing axis 1 scores and increasing axis 2 scores. Increasing relative abundances of low P bacteria and decreasing relative abundances of high P and high N bacteria (see TITAN results below) were associated with decreasing axis 1 scores and increasing axis 2 scores. Relative abundances of low N bacteria increased with decreasing axis 1 scores.

### 3.2. Bacterial Indicators and Relationships with TP and TN

**Figure 3.**
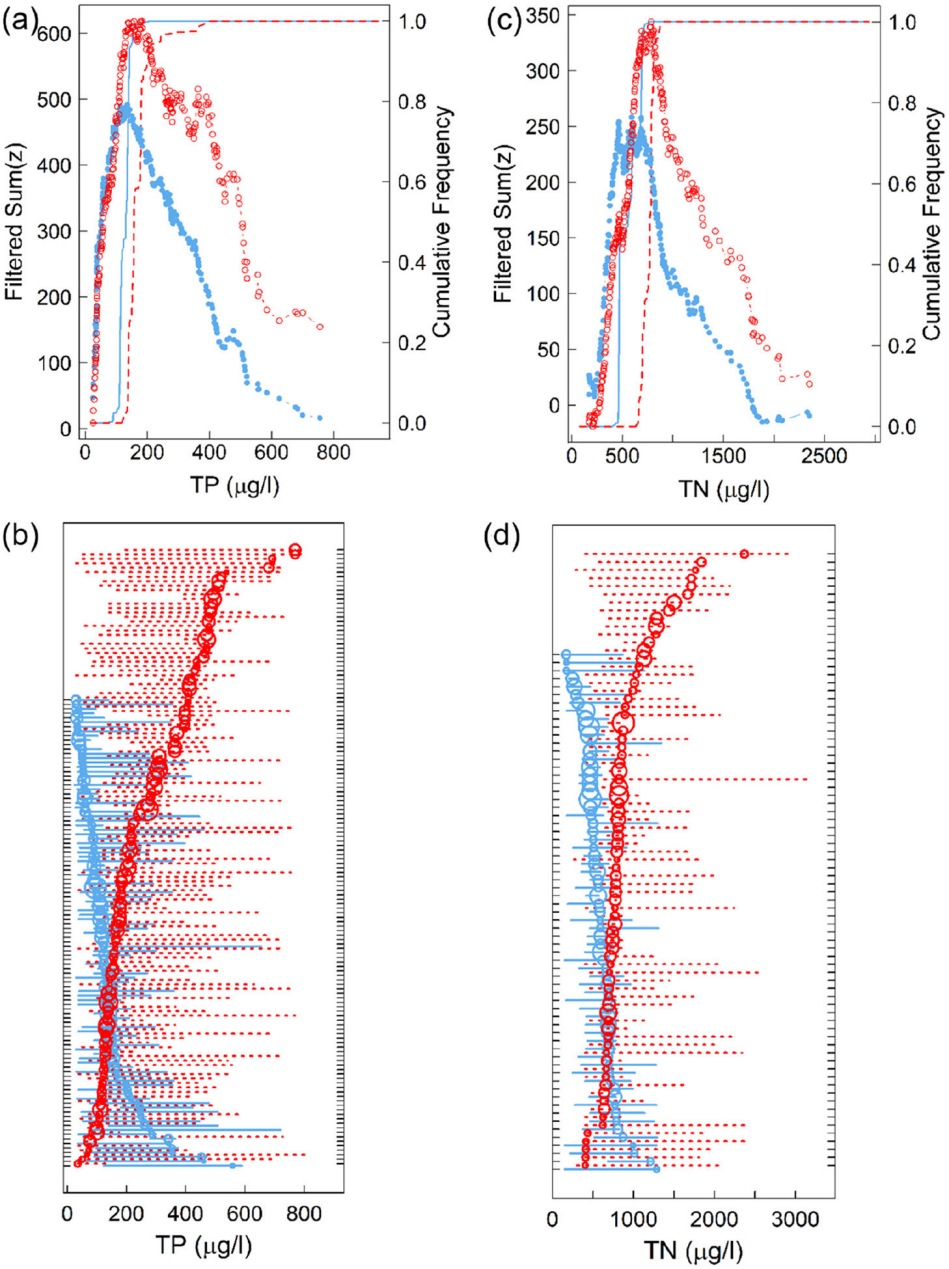
Threshold indicator taxa analysis showing sumZ scores and change points of ASVs for TP (**a**,**b**) and TN (**c**,**d**). Filtered refers to results using only ASVs identified as being pure (consistent response direction in >95% of bootstrap replicates) and reliable (consistently significant responses in >95% of bootstrap replicates). Red shows increaser sumZ scores and ASVs; blue shows decreaser sumZ scores and ASVs. For (**a**,**c**), circles; show the sumZ scoses of decreaser tend increaser ASVs at each observed nutrient concentration, and cumulative frequencies show the distribution of assemblage change points (max sumZ) based on 100 bootstraps. For (**b**,**d**), open circles show change points for each ASV (y-axis tick mares) scaled according to magnitude; of z scores, and dotted liners show the 5th and 95th quantiles based on 1000 bootstraps. Narrow peaks in sumZ scores, steep increases in the cumulative frequency curves, and multiple ASV change points occurring within a narrow range of TP or TN concentrations suggest nutrient thresholds at these concentrations. Broad peaks in sumZ scores, gradual increases in cumulative frequency curves, and gradual addition of ASV change points indicate more gradual responses to nutrient concentration and a longer gradient of community change.

Of the 429 ASVs used in TITAN, 105 were associated with lower TP concentrations (low P bacteria) and 138 were associated with higher TP concentrations (high P bacteria) among sites (Supplementary Table S2). Bootstrapped assemblage change points indicated that the greatest changes in low P bacteria occurred between 110 and 152 μg TP/L, with an overall change point at 136 μg TP/L (Figure 3a, Table 1). Of the 105 low P ASVs, most had change points within a narrow range of TP concentrations when compared to those of high P ASVs (Figure 3b, Supplementary Table S2). Bootstrapped assemblage change points indicated that the greatest changes in high P bacteria occurred between 137 and 243 μg TP/L, with a change point at 183 μg TP/L (Figure 3a, Supplementary Table S1). Of the 138 high P ASVs, most had broad distributions of bootstrapped change points when compared to those of low P ASVs, indicating that these high P ASVs had gradual changes in their relative abundances and occurrences across a wide range of TP concentrations (Figure 3b, Supplementary Table S2).

Of the same 429 ASVs used in TITAN, 63 were associated with lower TN concentrations (low N bacteria) and 74 were associated with higher TN concentrations (high N bacteria) among sites (Supplementary Table S3). Bootstrapped assemblage change points indicated that the greatest changes in low N bacteria occurred between 462 and 695 μg TN/L, with an overall change point at 467 μg TN/L (Figure 3c, Table 2). Bootstrapped assemblage change points indicated that the greatest changes in high N bacteria occurred between 665 and 812 μg TN/L, with an overall change point at 772 μg TN/L (Figure 3c, Table 2). In general, most low N and high N ASV change points occurred within narrow ranges of the total TN gradient, but a greater proportion of high N ASVs than low N ASVs had broad bootstrapped distributions of change points, indicating that these high N ASVs had more gradual changes in their relative abundances and occurrences than did low N ASVs as TN concentrations increased (Figure 3d, Supplementary Table S3).

**Figure 4.**
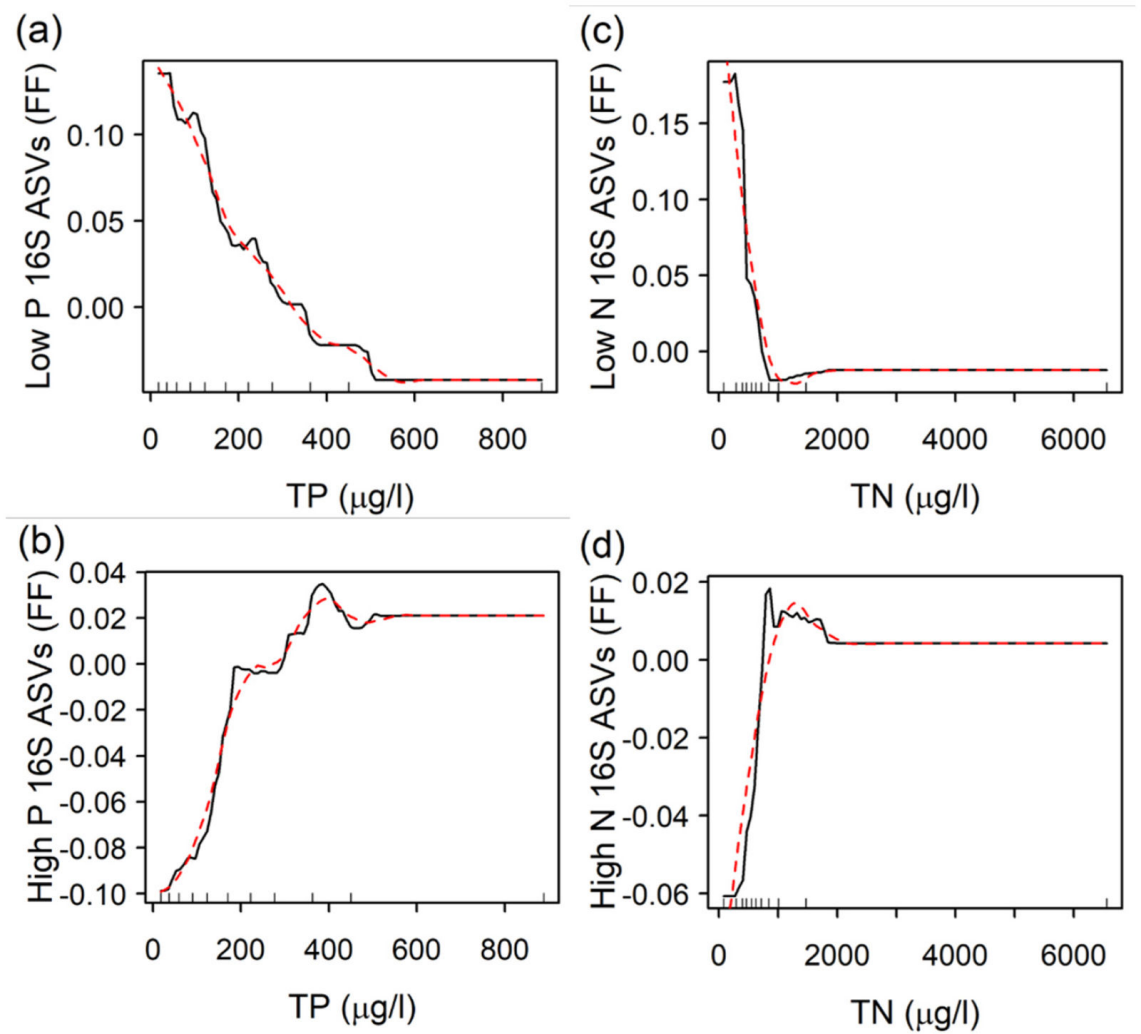
Partial dependence plots from boosted regression trees showing responses of bacterial metrics to TP(**a**,**b**) or TN (**c**,**d**) while controlling for the average effect of other variables. FF = fitted functions. Rug plots show deciles of predictor values.

Relative abundances of low P and low N ASVs decreased and those of high P and high N ASVs increased as watershed percent agriculture and nutrient concentrations increased (Table 3). Boosted regression tree models explained 54–67% of the deviance in bacterial metrics, and the cross-validated deviance explained ranged from 45 to 56% (Table 4), showing that the models performed similarly when predicting withheld data. TP was the most important predictor for low P ASVs, high P ASVs, but also high N ASVs, while TN was the most important predictor of only low N ASVs. Partial dependence plots identified ranges of TP and TN gradients within which large changes in bacterial metrics occurred (Figure 4). Relative abundances of low P bacteria had a small decrease from 42 to 61 μg TP/L followed by their largest decrease from 108 to 184 μg TP/L and smaller decreases from 241 to 311, 352 to 378, and 494 to 509 μg TP /L. High P bacteria had a small increase in relative abundance from 36 to 82 μg TP/L followed by their largest increase from 94 to 184 μg TP/L and another small increase from 287 to 356 μg TP/L. Relative abundances of low N bacteria had a large decrease from 271 to 459 μg TN/L followed by a smaller decrease from 459 to 859 μg TN/L. High N bacteria gradually increased in relative abundance from 279 to 756 μg TN/L.

**Figure 5.**
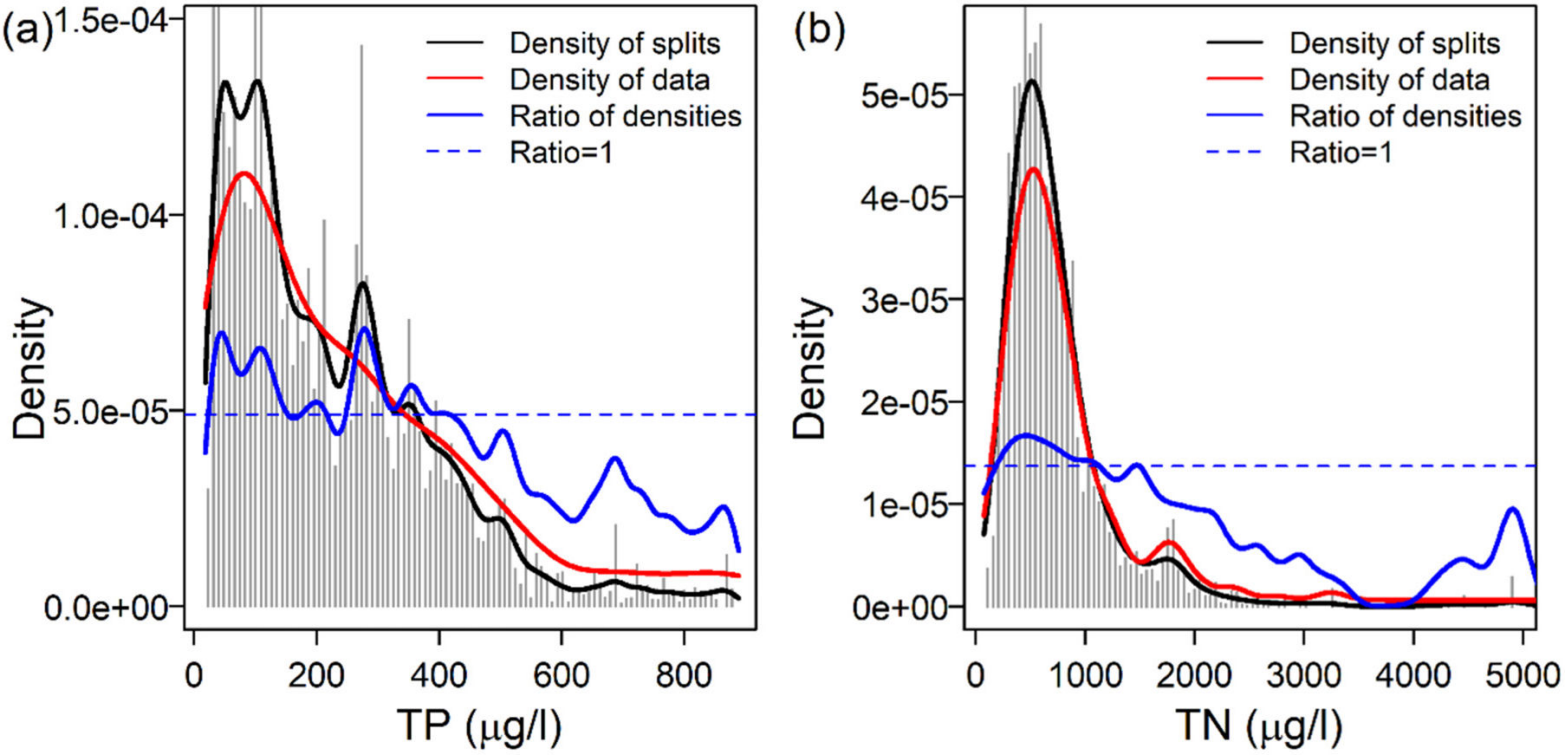
Gradient Forest analysis results showing bacterial ASV assemblage responses to TP (**a**) and TN (**b**). Compositional change based on aggregating ASV responses were determined lay split importance and values along TP and TN gradients (bars) and the ratios (blue lines) of split density (black lines) to data density (red lines). Peaks and regions of standardized split density plots with ratios above 1 (horizontal dashed blue lines) mark portions of the TP or TN gradient within which ASV compositional change is relatively greater other points along the nutrient gradient.

Gradient forest analysis included 278 ASVs with R^2^ values > 0 (maximum = 0.57, mean = 0.13, median = 0.10), including 61 with R^2^ > 0.20 (Supplementary Table S4). Peaks in the standardized split density plot showed that the greatest changes in bacterial assemblage structure occurred between 24 and 75, 75 and 152, and 250 and 325 μg TP/L, with smaller changes occurring between 174 and 217 and 325 and 386 μg TP/L (Figure 5). For TN, a gradual change in bacterial assemblage structure occurred between 193 and 928 μg/L, with a small second response around 1458 μg/L (Figure 5).

### 3.3. Summary of Changes in Bacterial Assemblages

**Figure 6.**
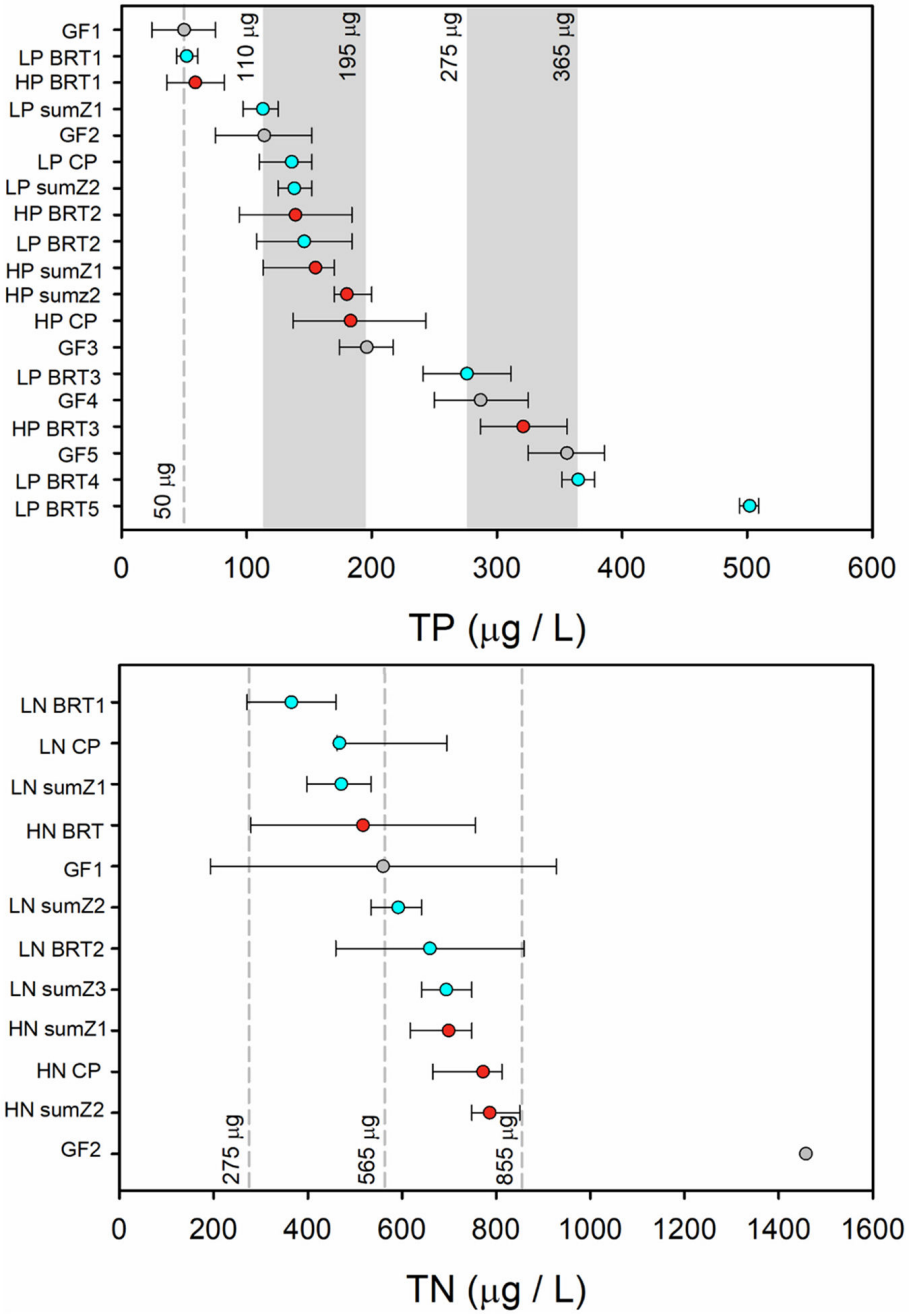
Summary of change points for TP and TN across the indicator analyses. Circles denote the mid-response value with horizontal bars show 5th to 95th quantiles for TITAN results and the beginning and end of responses for boosted regression trees and gradient forest results. Light blue circles are for low nutrient taxa, red circles are for high nutrient taxa, and gray circles are assemblage changes from gradient forest analytes. Bacterial responses occurred at multiple values along the TP or TN gradients and those are noted as sumZ1, sumZ2, BRT1, BRT2, etc. Vertical dashed lines and gray bars mark concentrations on the TP or TN gradients with substantial changes in bacterial assemblage. (LP = low phosphorus, LN = low nitrogen, HP = high phosphorus, HN = high nitrogen, CP = change point, BRT = boosted regression tree analysis, GF = gradient forest analysis). GF2 has no horizontal bars because it only briefly exceeded the density-of-splits to density-of-data ratio of 1.

Complementary results from these statistical analyses showed that bacterial assemblages were strongly associated with TP and TN gradients. Results were collated to identify TP and TN concentrations at which notable changes in bacterial assemblages occurred (Figure 6, Tables 1 and 2). In general, changes along TP and TN gradients were marked by shifts from dominance by low nutrient ASVs to high nutrient ASVs with major points of overall community change being highlighted by TITAN change points, steep portions of partial dependence plots, and peaks in gradient forest split density plots. Bacterial assemblages began having small changes from 24 to 82 μg TP/L with midpoints of multiple responses occurring around 50 μg TP/L. This initial change was followed by larger changes, including continued decreases in relative abundances of low P taxa, increases in those of high P taxa, TITAN change points, and additional large changes in gradient forest analysis between 110 and 195 μg TP/L. The final major changes in bacterial assemblages occurred between 275 and 365 μg TP/L, after which high P taxa dominated. The body of statistical evidence suggests that the bacterial assemblage structure experienced large, gradual changes with initial large decreases in relative abundances of low N ASVs from 275 to 565 μg TN/L followed by the greatest increase in relative abundances of high N ASVs and bootstrapped change points for high N taxa from 565 to 855 μg TN/L.

### 3.4. Taxonomy and Phylogenetic Analysis

**Figure 7.**
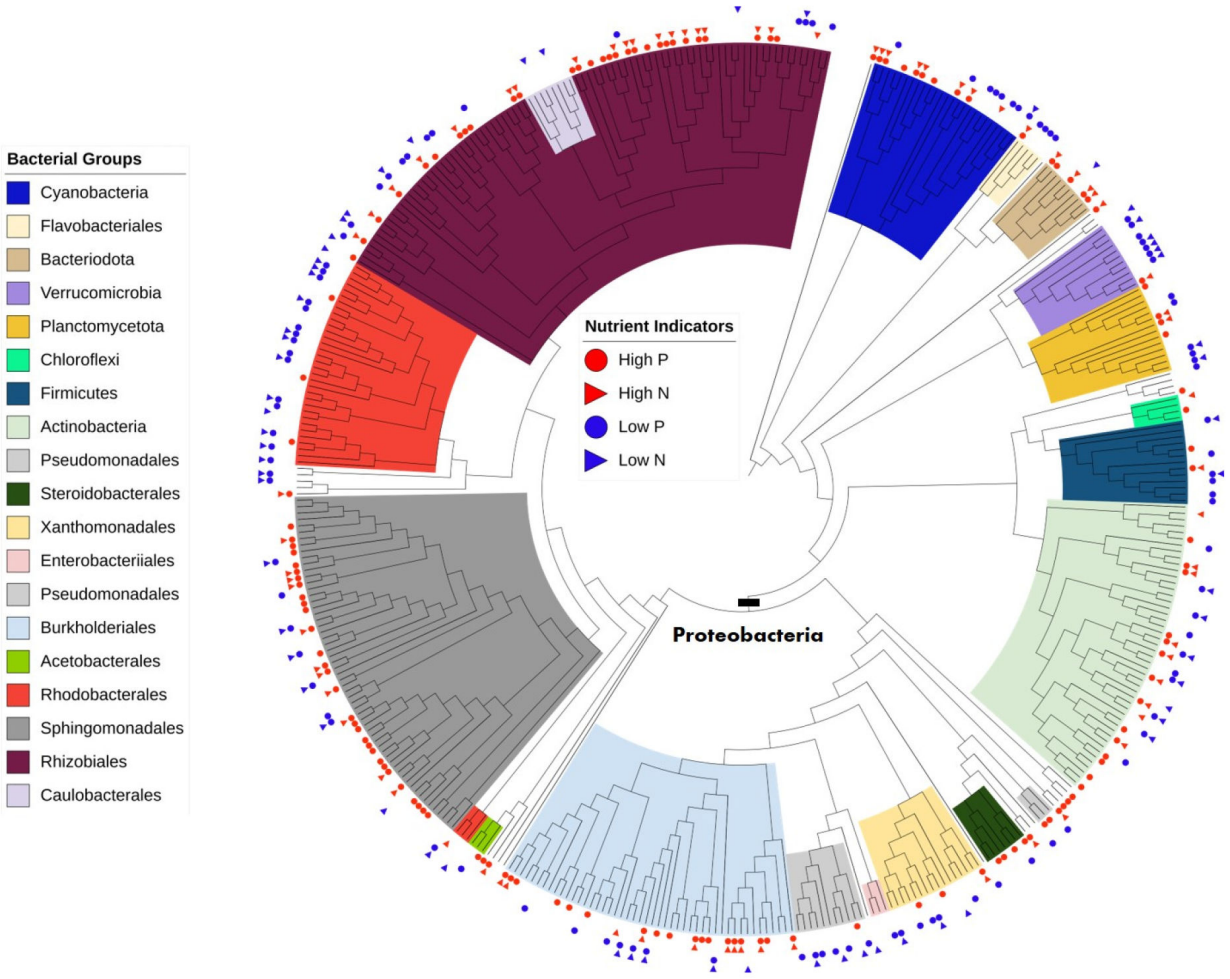
Phylogenetic tree of the 429 ASVs comprising 75% of the total relative abundance among all samples. Indicator ASVs from TITAN are marked. Major bacterial taxonomic groups are color coded in the legend in the order they appear in the tree, clockwise from the cyanobacteria near the top. Groups with only 1 or 2 members are unmarked.

Phylogenetic analysis of the 429 ASVs determined how the different types of indicators are spread amongst the various bacterial orders (Figure 7). Most bacterial phyla and classes were recovered as monophyletic with a few exceptions (e.g., Caulobacterales nested within Rhizobiales, and Rhodobacterales and Pseudomonadales are not monophyletic), though investigating the evolutionary history of bacteria was not the goal of the analysis. All clades with more than four ASVs had at least one ASV that was found to be an indicator of nutrient condition. Many ASVs responded to both TP and TN concentrations with 67 ASVs as indicators of both high P and high N and 45 ASVs as indicators of both low P and low N.

**Figure 8.**
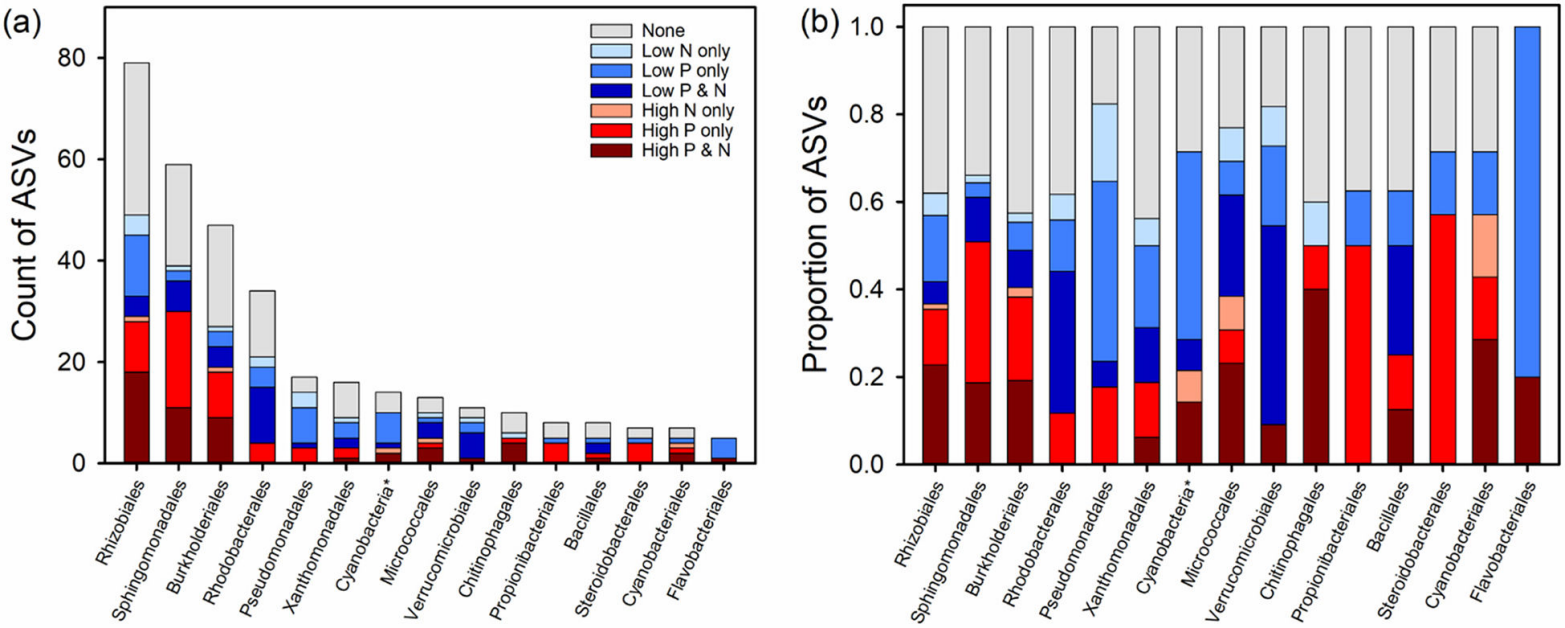
Breakdown of the indicator ASVs within the top 15 bacterial groups by count (**a**) or by proportion (**b**).

The majority of ASVs (N = 285) were members of the phylum Proteobacteria (Figure 7, Table 5). Other phyla in the top 75% with more than 10 ASVs included Actinobacteria (48 ASVs), Cyanobacteria (26 ASVs), Bacteroidota (16 ASVs), Firmicutes (14 ASVs), Planctomycetota (14 ASVs), and Verrucomicrobiota (11 ASVs) (Figures 7 and 8). Bacterial phyla with few ASVs (N < 5) included Chloroflexi, Acidobacteriota, Gemmatimonadota, Desulfobacterota, Deinococcales, Fusobacteriota, Myxococcota and Nitrospirota.

Within the Proteobacteria, the order Rhizobiales had the most ASVs (N = 79) and also the most ASVs found to be indicators in TITAN (N = 49) with 18 ASVs that were both indicators for high P and high N conditions, 10 ASVs that were indicators of high P, 1 ASV that was a high N indicator, 4 ASVs that were both indicators for low P and low N, 12 ASVs that were indicators of low P, and 4 ASVs that were indicators of low N (Table 5). Other seemingly important orders found within Proteobacteria included Sphingomonadales (59 total ASVs, 39 ASVs as indicators with 11 ASVs as indicators of both high P and high N and 19 ASVs as indicators of only high P), Burkholderiales (47 total ASVs, 27 ASVs as indicators with 9 ASVs as indicators of both high P and high N and 9 ASVs as indicators of only high P), Rhodobacterales (34 total ASVs, 21 ASVs as indicators with 4 ASVs as indicators of only high P, 11 ASVs as indicators of both low P and low N, and 4 ASVs as indicators of only low P), Pseudomonadales (16 total ASVs with 13 ASVs as indicators with 3 ASVs as indicators of just high P, 7 ASVs as indicators of only low P, and 3 ASVs as indicators only low N), and Xanthomonadales (16 total ASVs, 9 ASVs as indicators with 2 ASVs as indicators of only high P, 2 ASVs as indicators of both low P and low N, and 3 ASVs as indicators of only low P). Outside the Proteobacteria, other potentially important phyla/orders include Micrococcales (13 total ASVs, 10 ASVs as indicators) of the Actinobacteria, Chitinophagales (10 total ASVs, 6 ASVs as indicators) of the Bacteroidota, Cyanobacteria (26 total ASVs, 18 ASVs as indicators), and Verrucomicrobiota (11 total ASVs, 9 ASVs as indicators).

Indicators of high and low nutrient conditions were dispersed across all bacterial groups (Figures 7 and 8). ASVs classified under Firmicutes, Verrucomicrobiota, Flavobacteriales, and Pseudomonadales, Rhodobacterales, and Xanthomonadales of Proteobacteria were more; often correlated with low nutrient concentrations. ASVs classified under Chitinophagales of Bacteroidota, and Burkholderiales, Rhizobiales, Sphingomonadales, and Steroidobacterales of Proteobacteria, were correlated with high nutrient conditions.

## 4. Discussion

Our analyses showed distinct changes in bacterial assemblages associated with gradients of TP and TN concentrations, and considerable numbers of ASVs were significant responders to the phosphorus and nitrogen conditions (from TITAN, 243 of 429 for TP and 137 of 429 ASVs for TN, and from gradient forest analysis, 278 of 429 ASVs). Metabarcoding resulted in more than 18,000 bacterial ASVs, of which the 429 most frequent, comprising 75% of the total relative abundance represented over 15 different bacterial phyla. This work adds to the growing field of genetic characterization of bacterial communities for indicators of ecological or environmental conditions in streams and can inform the future applications of bacterial indicators in monitoring programs [26,36].

Our study identified many bacterial taxa that have significant relationships with either or both TP and TN. Most of these relationships show responses to TP over a concentration range of 25–195 μg/L and to TN over a range of 275–855 μg/L (Figure 5), which is similar to the ranges found for diatom responses in this watershed (20–150 μg TP/L and 150–850 μg “TN/L), though diatom assemblages tended to respond at much lower TP concentrations [41]. In general, TITAN results suggested that tow nutrient bacterial ASVs exhibited sharp declines in their relative abundances and occurrences as nutrient concentrations increased. This pattern could be due to greater numbers of high nutrient ASVs that, despite mostly having gradual increases across wider ranges of nutrient conditions, quickly and cumulatively outcompeted low nutrient ASVs as nutrient concentrations increased. While the bacterial indicator ASVs described herein likely respond to nutrient concentrations, they also could be responding to nutrient-related changes in other benthic community members [68-71].

Non-photosynthesizing bacterial groups fill biochemical roles that are very different from those of cyanobacterial or algal members of periphyton communities. For example, Sphingomonadales are known for secreting extracellular matrices as they proliferate, Actinobacteria often function as decomposers, and some Rhizobiales (e.g., *Rhizobium, Bradyrhizobium, Mesorhizobium, Sinorhizobium Azorhizobium,* and *Allorhizobium*) are major players in symbiotic N2-fixation in nature [72]. Bacteria also are important contributors to nutrient cycling within periphyton and produce extracellular enzymes that are important to phosphorus, nitrogen, and carbon uptake from organic sources [19], and their enzyme activity can be stimulated by algal productivity associated with greater amounts of nutrients [73-76]. More studies are necessary to uncover whether the bacterial indicators found herein are responding directly or indirectly to TP and TN conditions, but that does not change their potential importance as indicators for biomonitoring. Our results are informative for developing nutrient indicators in other regions, and although the data and analyses from this work are based on sites within a single watershed, the breadth of temporal coverage and the common watershed scale of monitoring programs increase the likelihood that these approaches are applicable to other, similar temperate watersheds.

DNA metabarcoding applied to biological monitoring is not without its limitations or biases. Complicating factors for the further development of these molecular genetic tools include concerns related to sample preservation [77], DNA extraction method [78,79], PCR bias [80,81], number of gene copies [82], uniformity across bioinformatic pipelines [83], and status of genetic databases [84]. However, research advances continue to address these concerns and minimize their impacts in molecular studies. For most taxa studied with DNA metabarcoding for environmental applications, critics often discuss comparisons of genetic data to traditional taxonomic methods, but such a concern does not apply to bacteria. Isolation and taxonomic identification of these periphyton-associated bacteria would require a myriad of culture methods. It is widely accepted that the percentage of bacteria that can be cultured is minimal relative to the estimated number of total bacterial species richness (10^7^–10^9^) [85,86]. DNA-based methods for the identification of bacterial communities still present the best opportunity to generate useful data in a timely and cost-effective manner.

Recent studies have demonstrated the potential utility of DNA metabarcoding for assessing responses of bacterial communities to anthropogenic impacts in multiple environmental contexts, and increasingly these responses are being captured in biotic indices informative to managers and decision-makers [87-89]. For instance, benthic bacterial communities have been shown to mirror responses of established macrofaunal indicators to nutrient enrichment associated with salmon farming [90], and 16S rRNA gene metabarcoding has been incorporated into a multi-trophic Metabarcoding Biotic Index found to be strongly correlated with those disturbances [91]. Biotic indices based solely on bacterial community profiles are similarly strongly correlated with impacts of salmon aquaculture, and those indices are sufficiently robust to be replicable across multiple independent laboratories [92], suggesting potential utility for routine biomonitoring. Of particular interest with respect to the current study is the fact that “de novo” methods for identifying bacterial indicators—i.e., taxonomy-free approaches based on a statistical analysis of ASVs—performed better in these contexts than a taxonomy-based approach in which 16S DNA sequence data was used to calculate a previously developed bacterial biotic index [93]. In addition, complementary analyses associated with our work also found that bacteria-nutrient relationships remained strong over time, except immediately following hydrologic disturbance, and that bacterial metrics best represent long-term nutrient concentrations, though they were still correlated with short-term concentrations as well [94]. In addition, comprehensive and complementary analyses of temporal variability among sites in our study found that bacteria-nutrient relationships remained strong over time, except immediately following hydrologic disturbance, and that bacterial metrics best represent long-term nutrient concentrations, though they also were still correlated with short-term concentrations [94]). These additional analyses also showed that indicators developed using multiple samples over time performed well despite temporal variability in both bacterial assemblages and nutrient concentrations.

Bacterial DNA metabarcoding has also proven useful to assess a variety of other anthropogenic environmental stressors. Recent studies demonstrate that indices based on bacterial community structure respond strongly to environmental impacts of oil and gas drilling and production [95], urban pollution in coastal mangrove forests [96], and metal pollution associated with cement plants [97]. Although many of these studies focus on benthic bacterial assemblages in estuarine and marine environments, similar approaches have been developed for freshwater habitats, including a recent study developing biological indicators of river health based on changes to bacterial communities in periphyton [98]. Our research thus adds to a growing literature illustrating the potential value of 16S metabarcoding for routine biomonitoring and provides further evidence of the utility of taxonomy-free approaches for developing informative indicators based on bacterial responses to environmental stress.

While our work highlights the potential utility of DNA metabarcoding applied to periphyton bacteria as nutrient indicators, these results suggest that other areas of metagenomic research would likely prove useful. Future studies could investigate not only the diversity of bacterial taxa present but also the genetic capabilities of those taxa related to processing nutrients. Studies of RNA from periphyton bacteria coupled with nutrient chemistry would likely show which nutrient processing genes are activated in response to nitrogen or phosphorus availability [99,100] (Graves et al. 2016; Dai et al. 2019) while also providing data on which bacterial taxa are functioning in these conditions [101,102] (Leff et al. 2015; Li et al. 2018). Analysis of these bacterial biochemical pathways could also be informative to how these bacteria interact with other members of the periphyton community. To date, microbial metagenomic nutrient processing studies have not been coupled with nutrient indicator analyses, but such work would be a valuable addition to environmental management tools applied to nutrient pollution.

## 5. Conclusions

Periphyton and other algal-based communities continue to be high-priority organisms collected as part of monitoring programs for their use as indicators of nutrient pollution in aquatic ecosystems [17,103]. Our analyses demonstrated that DNA metabarcoding applied to periphyton-associated bacteria provides similar results to DNA metabarcoding applied to benthic diatoms for quantifying nutrient responses in a watershed affected by agricultural and urbanization stresses. Multiple statistical methods identified assemblage changes along TP and TN gradients that could serve as possible targets for decision-makers tasked with mitigating nutrient inputs. While the results indicated that potential TP concentration targets based on bacterial indicators were higher than those found for diatom indicators in the same watershed, these responses still provide useful ecological insights into how nutrient pollution affects aquatic ecosystems. These targets may be informative for the development of nutrient criteria related to land use, best management practices for reducing nutrients in watersheds, and discharge permits. Further development of technologies, methods, databases, and analytical methods will continue to improve the capabilities of applying genetic/genomic approaches to biological monitoring.

## Supplementary Material

Supplement1

## Figures and Tables

**Table 1.** Summary of TP responses (Sorted by mid-response). CP is change point (mid-response) for TITAN results with start and end being 5th and 95th percentiles of bootstrapped change points. BRT = boosted regression trees, GF = gradient forest analysis, LP = low phosphorus ASVs, HP = high phosphorus ASVs. The sumZ responses are from TITAN distributions of bootstrapped community change points.

	Start of Response	Mid-Response	End of Response
Response	(μg/L)	(μg/L)	(μg/L)
GF TP1	24	50	75
BRT LP1	44	52	61
BRT HP1	36	59	82
LP sumz1	97	113	125
GF TP2	75	114	152
LP CP	110	136	152
LP sumz2	125	138	152
BRT HP2	94	139	184
BRT LP2	108	146	184
HP sumz1	113	155	170
HP sumz2	170	180	200
HP CP	137	183	243
GF TP3	174	196	217
BRT LP3	241	276	311
GF TP4	250	287	325
BRT HP3	287	321	356
GF TP5	325	356	386
BRT LP4	352	365	378
BRT LP5	494	502	509

**Table 2.** Summary of TN responses (Sorted by mid-response). CP is change point (mid-response) for TITAN results with start and end being 5th and 95th percentiles of bootstrapped change points. BRT = boosted regression trees, GF = gradient forest analysis, LN = low nitrogen ASVs, HN = high nitrogen ASVs. The sumZ responses are from TITAN distributions of bootstrapped community change points. GF2 had no discernable start or end of response because it only briefly exceeded the density-of-splits to density-of-data ratio of 1.

	Start of Response	Mid-Response	End of Response
Response	(μg/L)	(μg/L)	(μg/L)
BRT LN1	271	365	459
LN CP	462	467	695
LN sumz1	398	471	534
BRT HN	279	517	756
GF1	193	560	928
LN sumz2	534	592	641
BRT LN2	459	659	859
LN sumz3	641	694	748
HN sumz1	617	699	748
HN CP	665	772	812
HN sumz2	748	786	850
GF2	-	1458	-

**Table 3.** Correlations among bacteria metrics, % watershed agriculture (% ag), total nitrogen (TN), and total phosphorus (TP). Bacteria metric correlations with % agriculture used means of all samples within each site (*n* = 25), whereas TP *n* = 280, TN *n* = 281.

	% ag	TN	TP
Low P	−0.84	−0.59	−0.68
High P	0.86	0.62	0.71
Low N	−0.77	−0.64	−0.47
High N	0.88	0.64	0.71
TP	0.85	0.66	
TN	0.65		

**Table 4.** Results from boosted regression tree models for each bacterial metric showing deviance explained as a percentage of the null deviance for observed data and of deviance explained using cross-validated (CV) data. EC = electrical conductivity.

				Relative Importance (%)		
	Observed Deviance Explained (%)	CV Deviance Explained (%)	CV Correlation	TP	TN	EC
Low P ASVs	0.59	0.45 ± 0.05	0.69 ± 0.02	55.0	25.1	19.9
High P ASVs	0.67	0.56 ± 0.06	0.77 ± 0.02	46.2	28.8	25
Low N ASVs	0.54	0.46 ± 0.08	0.70 ± 0.04	13.7	76.2	10.1
High N ASVs	0.60	0.50 ± 0.10	0.79 ± 0.03	40.5	24.6	34.9

**Table 5.** Nutrient indicator ASVs by bacterial phylum/order.

Phylum	Order	Total	No. Indicators	High P and N	High P Only	High N Only	Low P and N	Low P Only	Low N Only
Proteobacteria	Rhizobiales	79	49	18	10	1	4	12	4
	Sphingomonadales	59	39	11	19		6	2	1
	Burkholderiales	47	27	9	9	1	4	3	1
	Rhodobacterales	34	21		4		11	4	2
	Pseudomonadales	16	13		3 **		1	7	3 **
	Xanthomonadales	16	9	1	2		2	3	1
	Caulobacterales	12	4				1		3
	Steroidobacterales	7	5		4			1	
	Acetobacterales	3	2		1			1	
	Enterobacterales	3	1					1	
	Gammaproteobacteria *	2	1	1					
	Azospirillales	1	1		1				
	Reyranellales	1	1	1					
	Rickettsiales	1	1	1					
	Alphaproteobacteria *	1	1	1					
	“PLTA13”	1	1					1	
	“PHOS-HD29”	1	1		1				
	Tistrellales	1	0						
Actinobacteriota	Micrococcales	13	10	3	1	1	3	1	1
	Propionibacteriales	8	5		4			1	
	Microtrichales	8	4	2			1	1	
	Corynebacteriales	5	3	1	1		1		
	“PeM15”	3	1					1	
	Gaiellales	3	1			1			
	Solirubrobacterales	3	1		1				
	Actinobacteriota *	3	0						
	Frankiales	1	1				1		
	Kineosporiales	1	1	1					
Cyanobacteria	Cyanobacteria *	14	10	2		1	1	6	
	Cyanobacteriales	7	5	2	1	1		1	
	“SepB-3”	4	2	1	1				
	Chroococcales	1	1	1					
Bacteroidota	Chitinophagales	10	6	4	1				1
	Flavobacteriales	5	5	1				4	
	Cytophagales	1	0						
*Firmicutes*	Bacillales	8	5	1	1		2	1	
	Exiguobacterales	4	3					3	
	Alicyclobacillales	1	1					1	
	Lactobacillales	1	0						
Planctomycetota	Pirellulales	7	4		1		2	1	
	Gemmatales	5	3	1				2	
	Isosphaerales	1	1		1				
	Planctomycetales	1	1	1					
Verruco-microbiota	Verrucomicrobiales	11	9	1			5	2	1
Chloroflexi	Chloroflexi *	4	2		1	1			
Acidobacteriota	Vicinamibacterales	2	2		2				
	Blastocatellales	2	0						
Gemmatimonadota	Gemmatimonadales	2	1		1				
Desulfo-bacterota	Desulfobulbales	1	1	1					
Thermi/Deinococci	Deinococcales	1	1	1					
Fuso-bacteriota	Fusobacteriales	1	0						
Myxococcota	Myxococcota *	1	0						
Nitrospirota	Nitrospirales	1	0						
		429	267	67	71	7	45	60	18

* Some ASVs could only be reliably identified to phylum/class.

** Within this order, one ASV was an indicator of high P and an indicator of low N.

## Data Availability

Bacterial 16S (and also diatom rbcL) and nutrient data are available at https://www.ncbi.nlm.nih.gov/bioproject/592969 and https://doi.org/10.23719/1504034.
