# Supplementary material for: Developing Indicators of Nutrient Pollution in Streams Using 16S rRNA Gene Metabarcoding of Periphyton-Associated Bacteria
Source: Water (Basel). Author manuscript; Available in PMC 2023 Jul 30. (PMC9534034; doi:10.3390/w14152361)
Supplement: Supplement1 [file NIHMS1831418-supplement-Supplement1.zip › water-1782772-supplementary.pdf]

Supplementary Materials: Supporting information for review and publication

Journal: Water

Title: Developing indicators of nutrient pollution in streams using 16S rRNA gene metabarcoding of periphyton-associated bacteria

Authors: Erik M. Pilgrim, Nathan J. Smucker, John Martinson, Huiyun Wu, Christopher T. Nietch, Marirosa Molina, John A. Darling, Brent R. Johnson

Table S1. Site Coordinates

Abbrev.	Site Name	Latitude/Longitude
BBC	Backbone Creek	39.0896 N 84.1690 W
BRF	Brushy Fork	39.1387 N 84.1316 W
FMC	Fourmile Creek	39.0973 N 84.0262 W
FMR	Fourmile Run	39.0547 N 84.1678 W
FVC	Fivemile Creek	39.1136 N 84.0203 W
GRR	Grassy Fork	39.1329 N 84.0152 W
HLR	Hall Run	39.1403 N 84.2592 W
HWR	Howard Run	39.1240 N 84.0073 W
KAR	Kain Run	39.0333 N 84.0819 W
LCF	Lick Fork	39.1472 N 84.1784 W
LRC	Lucy Run	39.0597 N 84.1806 W
LRN	Light Run Trib	38.9930 N 84.0590 W
MOF	Moores Fork	39.1977 N 84.0608 W
PLR	Pleasant Run	39.1125 N 84.0422 W
S1	Sugarcamp Run	39.1347 N 84.2325 W
SAR	Salt Run	39.1372 N 84.2458 W
SHO	Shayler Run at Olive Branch	39.0883 N 84.2217 W
SHR	Shayler Run at Perintown	39.1178 N 84.2164 W
SOR	Solomon Run	39.2022 N 83.9097 W
UST	Stonelick Creek	39.2564 N 84.0125 W
SYC	Sycamore Creek	39.2058 N 83.9450 W
FVM	Upper Fivemile Creek	39.1073 N 83.9325 W
890	Tribuitary To Dodson Creek	39.2043 N 83.7642 W
ULR	Ullery Run	39.0021 N 84.1515 W
USR	Upper Salt Run	39.1171 N 84.2593 W

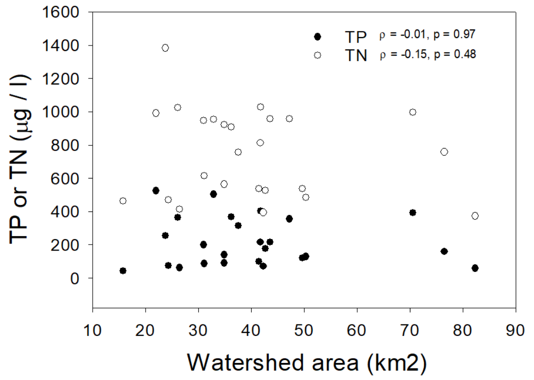

Figure S1. Comparison of TP and TN concentrations to watershed area.

Table S2. Total phosphorus TITAN results for amplicon sequence variants (ASV) with >95% purity and 95% reliability, change points (CP), sample frequencies (Freq), z-scores, and percentile distributions of bootstrapped change points. Indicator column denotes high P or low P ASVs that increased or decreased, respectively, with greater TP concentrations.

ASV	CP	Freq	% Freq	Z-Score	0.05	0.10	0.50	0.90	0.95	Indicator
ASV18	34.33	234	0.84	4.66	33.82	34.38	37.05	118.49	138.72	high
ASV39	50.79	184	0.66	4.08	39.38	41.20	51.73	460.46	691.47	high
ASV289	67.64	29	0.10	3.25	63.55	65.07	110.20	272.56	800.75	high
ASV398	72.16	70	0.25	3.53	44.86	50.70	102.47	182.20	414.97	high
ASV166	73.53	112	0.40	3.35	36.56	36.61	71.01	207.20	271.72	high
ASV5	76.71	267	0.95	8.92	60.61	67.62	76.93	100.90	102.47	high
ASV369	78.19	47	0.17	3.85	55.43	55.81	78.19	715.96	727.25	high
ASV27	99.02	111	0.40	10.85	86.49	93.81	102.13	158.41	175.23	high
ASV49	99.02	231	0.83	9.11	69.83	71.90	96.31	102.47	116.35	high
ASV156	102.13	71	0.25	3.63	74.21	75.60	221.70	428.35	449.39	high
ASV24	108.89	237	0.85	4.87	44.29	63.38	155.00	460.46	470.53	high
ASV211	108.89	45	0.16	4.80	72.16	77.71	114.21	188.10	591.65	high
ASV69	111.20	95	0.34	11.00	109.76	109.91	113.20	182.75	263.94	high
ASV55	112.61	77	0.28	6.01	101.00	111.20	177.27	490.72	490.72	high
ASV50	116.35	135	0.48	4.49	38.80	67.20	120.18	177.03	265.88	high
ASV116	116.35	183	0.65	6.09	110.77	111.49	155.60	406.17	415.48	high
ASV314	116.35	30	0.11	6.38	113.11	114.13	213.29	477.51	488.64	high
ASV397	116.35	31	0.11	4.98	113.20	114.13	216.31	490.72	508.77	high
ASV54	118.81	203	0.73	5.28	111.20	113.62	118.81	442.51	460.46	high
ASV155	118.81	115	0.41	3.73	71.01	116.35	155.34	431.05	483.40	high
ASV198	122.80	119	0.43	4.85	37.30	48.05	122.80	291.49	557.52	high
ASV388	122.80	111	0.40	6.17	119.83	121.52	123.18	243.27	274.33	high
ASV384	123.08	91	0.33	4.52	115.20	116.67	136.06	479.72	555.52	high
ASV178	123.47	44	0.16	3.72	73.27	102.13	129.78	305.73	540.60	high
ASV349	128.22	44	0.16	7.37	121.52	122.80	170.87	719.22	727.25	high
ASV4	129.57	241	0.86	7.68	123.08	125.80	143.98	210.07	223.04	high
ASV111	129.57	153	0.55	5.04	50.08	121.08	159.71	285.90	369.40	high
ASV129	129.90	101	0.36	8.98	123.08	125.39	182.75	269.73	272.33	high

ASV141	129.90	166	0.59	6.52	119.83	121.52	221.07	395.90	411.49	high
ASV324	129.90	37	0.13	5.45	118.44	122.80	162.25	537.74	683.45	high
ASV365	130.74	89	0.32	10.52	110.63	111.20	155.00	243.06	329.61	high
ASV113	133.20	102	0.36	11.51	129.69	129.90	162.25	307.92	364.06	high
ASV301	136.06	65	0.23	6.56	129.78	131.37	157.56	232.70	271.26	high
ASV330	136.06	77	0.28	5.30	122.51	127.02	139.44	732.72	768.50	high
ASV37	137.70	119	0.43	6.92	76.70	112.02	139.52	395.50	477.51	high
ASV93	137.70	118	0.42	7.84	130.73	135.23	180.33	343.67	364.75	high
ASV297	138.72	107	0.38	13.50	123.08	124.44	146.89	210.69	210.88	high
ASV78	139.29	97	0.35	5.77	100.38	102.13	210.69	313.38	557.52	high
ASV197	139.29	143	0.51	10.72	130.74	138.72	175.95	256.71	273.39	high
ASV53	139.52	163	0.58	4.87	129.90	137.70	188.63	513.59	768.50	high
ASV62	143.98	102	0.36	2.71	43.73	72.39	173.22	604.35	630.30	high
ASV209	143.98	131	0.47	4.13	138.95	142.18	182.14	324.21	423.41	high
ASV109	147.68	136	0.49	7.33	124.23	129.90	149.88	180.94	205.57	high
ASV114	153.97	67	0.24	8.67	119.13	123.85	155.34	440.83	505.93	high
ASV77	155.34	159	0.57	6.14	78.19	81.60	155.86	402.12	424.85	high
ASV428	155.34	51	0.18	4.00	67.64	79.73	163.94	281.92	290.12	high
ASV148	156.20	99	0.35	5.78	154.32	156.66	180.33	434.33	490.72	high
ASV32	157.17	167	0.60	5.58	138.72	155.26	208.30	435.68	439.80	high
ASV43	162.25	190	0.68	3.24	36.30	119.51	159.38	719.22	727.25	high
ASV181	162.25	105	0.38	3.44	86.49	98.58	162.25	431.05	478.68	high
ASV335	163.94	33	0.12	6.09	149.88	152.95	205.70	680.18	727.25	high
ASV300	166.36	92	0.33	4.33	137.67	139.11	180.33	335.14	679.82	high
ASV176	170.35	90	0.32	9.54	111.45	116.35	151.77	177.29	206.88	high
ASV343	170.35	30	0.11	9.05	141.39	163.94	171.19	486.99	513.59	high
ASV194	176.88	132	0.47	8.84	119.51	120.53	176.80	362.60	402.15	high
ASV212	176.88	80	0.29	9.12	123.47	128.22	178.53	208.36	210.69	high
ASV305	176.88	16	0.06	4.07	168.21	171.23	177.00	494.12	653.87	high
ASV353	177.27	67	0.24	8.84	148.86	156.66	214.45	334.97	349.55	high
ASV219	180.33	117	0.42	8.26	146.20	148.07	177.03	271.29	308.69	high
ASV363	180.33	25	0.09	4.63	156.66	176.69	208.30	477.51	494.12	high
ASV407	180.33	71	0.25	6.70	110.32	111.20	182.14	483.40	485.48	high
ASV40	182.75	194	0.69	4.77	36.91	42.53	157.17	424.12	464.20	high
ASV108	182.75	205	0.73	6.06	76.70	130.10	185.96	517.61	571.70	high
ASV208	182.75	70	0.25	8.10	161.06	162.81	286.35	397.01	510.84	high
ASV310	194.69	44	0.16	11.13	131.37	137.08	196.21	361.86	557.52	high
ASV377	194.69	49	0.18	4.64	50.13	116.34	189.75	392.20	755.00	high
ASV140	205.70	82	0.29	11.34	133.20	138.88	188.63	226.80	306.78	high
ASV404	206.21	74	0.26	7.22	198.06	205.82	271.41	431.05	500.58	high
ASV75	208.89	152	0.54	4.37	92.26	122.48	210.85	679.82	679.82	high
ASV215	210.69	67	0.24	6.66	119.83	206.80	216.01	247.51	274.85	high
ASV416	211.55	44	0.16	10.83	121.52	139.29	251.66	467.45	485.02	high
ASV74	214.82	159	0.57	6.97	211.70	213.29	216.57	271.29	276.82	high
ASV71	214.82	157	0.56	5.40	85.80	98.02	214.78	310.00	364.14	high
ASV291	214.82	20	0.07	6.99	138.11	158.92	243.50	369.40	427.67	high
ASV395	214.82	49	0.18	4.76	205.69	212.98	272.32	727.25	727.25	high
ASV193	216.01	66	0.24	5.46	133.20	153.97	256.71	679.82	768.50	high

ASV306	222.34	29	0.10	6.95	207.79	210.00	239.07	362.49	483.40	high
ASV303	236.25	103	0.37	3.17	50.71	60.74	236.25	346.76	380.89	high
ASV168	243.27	104	0.37	8.29	195.01	236.25	247.49	354.41	364.03	high
ASV402	269.71	43	0.15	15.87	267.21	269.71	275.39	293.88	298.70	high
ASV295	271.43	83	0.30	9.68	160.60	163.94	271.43	315.44	364.43	high
ASV408	278.75	22	0.08	5.89	101.00	119.13	285.85	537.79	719.22	high
ASV94	281.07	94	0.34	6.33	48.37	270.77	290.12	516.42	517.35	high
ASV432	285.85	28	0.10	5.37	99.39	101.33	285.35	335.14	347.11	high
ASV12	294.26	81	0.29	11.56	171.18	176.11	269.87	301.27	305.73	high
ASV200	294.26	84	0.30	5.79	123.47	214.82	332.37	377.63	650.25	high
ASV302	294.26	49	0.18	11.00	153.97	212.90	294.63	380.89	395.90	high
ASV451	305.71	47	0.17	9.24	163.94	227.51	273.81	359.23	373.55	high
ASV124	307.92	67	0.24	12.55	214.85	222.34	301.27	320.83	407.41	high
ASV146	307.92	101	0.36	10.26	146.95	160.50	298.70	392.20	393.31	high
ASV391	307.92	27	0.10	5.84	241.02	245.21	313.37	727.25	727.25	high
ASV97	310.00	101	0.36	9.67	138.11	139.59	209.59	335.14	335.14	high
ASV128	362.60	114	0.41	8.69	177.09	182.75	365.84	471.18	477.51	high
ASV143	362.60	54	0.19	9.72	255.84	318.82	363.32	453.06	470.53	high
ASV224	362.60	60	0.21	6.39	216.27	221.64	362.49	422.36	477.51	high
ASV61	365.84	118	0.42	4.26	142.11	147.95	392.20	520.19	569.70	high
ASV151	369.40	127	0.45	9.84	111.74	114.21	355.78	369.13	377.59	high
ASV142	396.61	84	0.30	3.59	57.61	62.85	272.47	421.28	460.46	high
ASV351	396.61	26	0.09	6.76	138.11	161.06	411.22	495.45	500.58	high
ASV431	396.61	18	0.06	6.28	349.89	353.96	395.50	485.48	490.72	high
ASV160	397.01	75	0.27	8.44	274.72	364.14	397.01	488.98	494.12	high
ASV237	397.01	21	0.08	7.85	156.20	157.17	380.89	755.00	768.50	high
ASV231	402.13	30	0.11	4.05	67.37	71.01	369.40	470.53	483.51	high
ASV192	408.53	69	0.25	4.26	56.12	177.90	417.21	497.38	500.58	high
ASV316	408.53	140	0.50	3.87	36.61	43.34	324.00	445.27	476.89	high
ASV385	408.53	21	0.08	5.33	110.93	196.06	405.46	412.56	412.57	high
ASV202	411.87	28	0.10	10.18	258.90	261.98	396.05	466.79	470.53	high
ASV277	411.87	37	0.13	9.92	213.87	226.80	354.80	422.36	466.98	high
ASV345	411.87	23	0.08	7.57	315.44	380.83	415.45	486.15	494.12	high
ASV19	423.41	228	0.81	2.29	29.19	29.25	128.22	431.05	563.45	high
ASV131	431.05	65	0.23	5.07	179.45	210.85	381.74	442.51	460.81	high
ASV177	436.52	87	0.31	4.65	83.20	99.26	392.20	463.12	466.79	high
ASV267	436.52	71	0.25	4.52	87.24	97.87	364.08	691.47	691.47	high
ASV145	460.46	96	0.34	7.67	112.61	116.64	449.39	470.53	488.64	high
ASV276	460.46	40	0.14	4.86	69.97	194.82	477.18	490.72	492.04	high
ASV125	470.53	82	0.29	4.90	60.61	143.98	412.56	471.18	476.42	high
ASV169	470.53	84	0.30	3.45	47.80	70.10	256.71	485.48	505.93	high
ASV180	470.53	40	0.14	12.78	396.61	442.51	466.79	474.76	477.51	high
ASV268	470.53	64	0.23	8.58	108.81	109.76	195.48	470.53	490.72	high
ASV59	477.51	72	0.26	4.76	136.06	359.72	477.51	499.26	559.54	high
ASV261	477.51	69	0.25	3.31	58.98	63.38	242.28	477.51	488.74	high
ASV319	477.51	45	0.16	9.10	216.31	396.96	483.40	523.10	571.70	high
ASV321	477.51	29	0.10	5.92	196.08	247.49	426.82	505.93	729.28	high
ASV352	483.40	19	0.07	7.75	141.16	156.30	477.84	510.84	630.30	high

ASV429	483.40	39	0.14	9.18	141.39	182.02	467.45	490.72	492.08	high
ASV183	490.72	88	0.31	6.76	140.86	143.19	439.12	490.72	492.80	high
ASV247	490.72	40	0.14	12.37	392.90	396.21	485.48	505.74	506.20	high
ASV199	494.12	56	0.20	8.43	393.05	395.90	483.40	507.44	591.65	high
ASV48	500.58	94	0.34	5.67	167.21	356.62	470.53	500.80	538.25	high
ASV299	510.84	53	0.19	8.24	109.76	120.53	359.68	511.48	520.19	high
ASV392	510.84	88	0.31	9.79	123.08	128.54	510.84	691.47	691.47	high
ASV250	520.19	57	0.20	6.62	208.28	271.43	510.84	590.16	699.50	high
ASV68	537.74	106	0.38	3.04	65.07	123.08	396.61	719.22	727.25	high
ASV171	679.82	44	0.16	7.28	155.34	206.06	510.84	727.25	727.25	high
ASV11	691.47	176	0.63	3.20	29.25	29.25	352.36	691.47	691.47	high
ASV331	691.47	77	0.28	4.88	50.79	67.78	511.25	691.47	715.60	high
ASV284	768.50	45	0.16	6.25	51.73	193.90	699.50	768.50	787.25	high
ASV364	768.50	33	0.12	8.28	195.88	217.83	307.92	768.50	787.25	high
ASV84	29.04	107	0.38	7.67	27.48	27.63	44.96	108.02	139.52	low
ASV102	29.04	72	0.26	5.76	21.99	23.78	29.04	261.97	354.03	low
ASV164	31.41	88	0.31	7.79	30.60	30.75	39.90	77.19	95.73	low
ASV285	31.41	82	0.29	6.01	29.10	29.19	31.41	75.81	87.77	low
ASV467	31.41	35	0.13	10.17	29.19	29.25	31.41	60.97	123.47	low
ASV57	32.65	165	0.59	5.16	32.15	32.30	49.23	130.18	349.92	low
ASV230	33.83	36	0.13	5.30	27.09	32.15	45.45	177.11	202.95	low
ASV355	33.83	57	0.20	4.46	29.25	31.65	34.10	98.90	237.50	low
ASV290	38.18	27	0.10	9.84	31.41	32.30	43.73	76.52	102.25	low
ASV463	38.18	7	0.03	11.48	31.47	35.85	38.18	75.83	76.45	low
ASV442	42.68	30	0.11	9.45	30.73	34.33	37.15	43.83	44.76	low
ASV82	50.08	93	0.33	6.82	32.15	32.19	50.31	110.10	113.63	low
ASV204	50.08	85	0.30	5.57	40.73	41.20	47.53	58.85	276.48	low
ASV123	50.70	62	0.22	5.77	43.71	50.00	86.54	195.82	290.12	low
ASV23	51.74	183	0.65	5.43	44.98	48.07	55.81	396.21	407.31	low
ASV334	53.44	39	0.14	5.29	33.23	33.25	53.44	271.27	326.89	low
ASV344	53.44	62	0.22	5.68	33.83	40.58	54.14	218.09	278.80	low
ASV36	55.81	169	0.60	5.42	47.92	48.15	69.97	417.23	418.33	low
ASV161	56.37	70	0.25	10.05	34.65	36.56	50.80	57.45	61.27	low
ASV245	56.37	37	0.13	5.35	24.45	32.30	50.16	105.16	265.35	low
ASV441	56.37	19	0.07	4.88	32.25	32.30	56.01	104.94	109.76	low
ASV234	56.80	59	0.21	10.69	37.25	42.47	56.80	97.42	102.59	low
ASV421	58.85	50	0.18	12.47	45.34	50.78	58.01	91.94	107.91	low
ASV240	60.74	77	0.28	4.53	29.04	33.00	57.87	76.53	286.62	low
ASV286	60.74	85	0.30	5.91	27.42	34.70	40.58	60.74	69.97	low
ASV362	60.74	38	0.14	8.56	43.32	53.15	62.50	100.90	216.02	low
ASV92	67.64	198	0.71	6.43	55.79	57.50	90.22	397.01	445.27	low
ASV218	78.19	52	0.19	5.17	29.09	76.45	80.55	265.88	271.27	low
ASV294	82.42	33	0.12	6.17	30.07	42.53	80.88	101.00	118.56	low
ASV185	84.47	59	0.21	3.89	56.80	62.85	83.24	424.80	460.46	low
ASV260	86.54	53	0.19	4.14	29.04	33.45	86.54	315.44	369.13	low
ASV122	87.52	32	0.11	7.57	73.53	80.88	88.40	130.93	170.35	low
ASV332	88.63	118	0.42	3.56	34.94	35.15	85.26	395.50	395.90	low
ASV73	89.74	190	0.68	5.20	42.53	43.34	88.52	92.26	248.18	low

ASV83	89.74	111	0.40	4.81	35.10	35.15	87.52	99.05	272.47	low
ASV137	89.74	83	0.30	8.99	42.37	55.78	84.47	129.78	137.70	low
ASV172	89.74	57	0.20	5.58	36.45	38.07	88.40	352.33	354.99	low
ASV239	89.74	59	0.21	11.06	80.88	82.42	89.74	98.17	123.92	low
ASV265	89.74	131	0.47	6.36	53.44	55.43	76.68	93.59	212.92	low
ASV382	89.74	26	0.09	6.60	36.56	37.10	89.74	97.87	139.44	low
ASV251	90.06	39	0.14	9.94	73.27	75.60	98.90	114.21	118.49	low
ASV52	95.69	117	0.42	11.00	60.74	75.60	95.69	216.27	290.12	low
ASV246	95.69	92	0.33	18.18	76.71	87.77	101.00	113.62	129.78	low
ASV30	99.02	230	0.82	6.05	44.76	44.96	100.90	352.36	354.41	low
ASV259	102.57	69	0.25	7.05	86.54	89.74	102.57	272.50	285.85	low
ASV127	109.91	80	0.29	10.45	64.62	89.74	135.55	266.75	271.43	low
ASV86	111.20	88	0.31	12.93	82.42	89.63	109.36	162.27	177.27	low
ASV287	111.75	17	0.06	7.34	36.91	37.05	111.20	133.39	137.08	low
ASV252	112.61	55	0.20	10.85	43.34	96.25	114.21	152.95	155.00	low
ASV311	112.61	64	0.23	12.82	36.73	36.82	123.08	163.94	169.18	low
ASV401	112.61	39	0.14	6.61	89.74	105.38	112.02	227.51	294.26	low
ASV152	114.13	82	0.29	8.74	89.74	93.50	122.51	266.75	269.23	low
ASV356	114.13	42	0.15	9.47	57.87	75.60	111.20	144.77	176.11	low
ASV91	116.35	161	0.58	11.67	111.18	111.49	118.97	157.17	180.33	low
ASV325	121.52	81	0.29	8.41	111.20	113.03	120.82	139.52	141.32	low
ASV13	122.80	264	0.94	6.04	118.81	122.51	152.95	307.92	653.87	low
ASV154	123.08	124	0.44	3.42	89.74	110.04	173.22	281.07	298.72	low
ASV281	123.08	49	0.18	8.55	87.64	89.63	110.77	171.43	210.85	low
ASV149	123.47	33	0.12	6.35	110.48	112.61	121.81	131.37	135.04	low
ASV322	123.47	99	0.35	7.88	63.38	80.88	122.80	156.72	164.50	low
ASV98	124.23	83	0.30	5.20	102.89	111.20	124.05	138.72	177.27	low
ASV271	129.90	56	0.20	6.74	89.74	105.38	128.56	268.17	269.71	low
ASV414	129.90	27	0.10	3.82	29.25	37.15	98.13	133.20	227.51	low
ASV440	129.90	22	0.08	4.83	110.48	118.81	131.37	202.80	206.61	low
ASV15	133.20	155	0.55	12.53	110.91	113.03	139.52	176.34	177.51	low
ASV3	136.06	223	0.80	5.21	39.38	48.07	107.94	240.58	359.62	low
ASV130	136.06	84	0.30	4.42	36.65	44.37	112.34	162.25	281.07	low
ASV368	136.06	43	0.15	10.60	37.05	107.94	135.73	170.59	178.58	low
ASV434	136.06	18	0.06	5.86	36.50	36.96	112.34	130.74	137.70	low
ASV20	137.70	173	0.62	9.94	113.62	130.12	143.19	207.91	213.29	low
ASV80	138.72	80	0.29	13.80	90.06	100.52	136.68	147.68	155.02	low
ASV107	139.29	101	0.36	6.88	116.35	123.81	139.29	194.69	208.89	low
ASV42	139.52	170	0.61	12.37	110.77	111.20	124.23	152.95	156.20	low
ASV203	139.52	85	0.30	4.27	50.47	112.61	139.48	264.62	294.63	low
ASV307	141.39	73	0.26	4.88	37.10	37.15	142.18	200.92	206.09	low
ASV99	143.98	134	0.48	8.88	112.45	141.39	154.83	210.00	232.74	low
ASV191	143.98	41	0.15	10.20	89.63	94.07	110.84	176.73	180.45	low
ASV233	147.68	84	0.30	5.07	118.81	126.02	163.94	305.71	307.78	low
ASV374	151.77	46	0.16	8.04	29.25	30.07	142.69	163.94	178.53	low
ASV87	155.34	175	0.63	6.84	89.62	98.90	144.37	161.07	170.91	low
ASV162	155.34	113	0.40	10.58	42.55	98.90	151.77	156.32	158.06	low
ASV213	155.34	85	0.30	8.14	36.71	41.33	141.39	170.15	171.19	low

ASV9	157.17	231	0.83	9.20	113.62	136.98	168.23	198.34	217.83	low
ASV147	163.94	52	0.19	3.78	30.07	45.92	163.94	271.27	335.14	low
ASV266	169.18	104	0.37	3.99	130.48	156.17	202.80	267.69	281.11	low
ASV1	177.27	280	1.00	6.77	133.20	137.70	180.38	354.99	359.73	low
ASV117	198.15	116	0.41	4.74	121.43	137.70	202.80	347.32	354.99	low
ASV47	202.80	108	0.39	6.10	36.30	36.56	171.23	212.17	214.45	low
ASV272	206.21	35	0.13	5.64	118.81	130.53	170.87	207.91	209.60	low
ASV38	214.82	131	0.47	4.92	32.15	48.16	182.14	223.04	276.44	low
ASV81	236.25	125	0.45	4.05	76.45	89.73	272.47	395.93	477.51	low
ASV51	245.44	161	0.58	5.56	180.33	182.75	247.49	395.50	423.41	low
ASV64	245.44	143	0.51	4.50	48.07	72.17	247.37	503.31	507.44	low
ASV396	247.49	35	0.13	4.18	50.08	73.53	216.31	264.14	266.27	low
ASV70	260.62	147	0.53	3.22	52.50	98.83	260.62	420.23	446.63	low
ASV2	261.18	264	0.94	6.05	139.29	170.35	261.43	483.74	507.44	low
ASV31	280.26	207	0.74	3.42	85.72	195.88	281.02	417.23	719.22	low
ASV273	285.85	40	0.14	4.98	37.15	50.70	269.71	289.99	299.27	low
ASV21	341.20	218	0.78	7.02	280.17	282.73	299.07	363.32	365.56	low
ASV360	341.20	54	0.19	3.37	35.10	35.15	317.11	346.76	349.59	low
ASV7	354.41	255	0.91	4.55	97.21	99.02	247.49	365.08	373.55	low
ASV22	354.41	223	0.80	3.73	55.23	102.91	263.39	355.39	364.77	low
ASV29	453.06	194	0.69	6.21	89.74	247.05	450.30	470.53	471.18	low
ASV67	460.46	95	0.34	2.80	30.75	55.51	290.49	464.20	467.45	low
ASV16	557.52	214	0.76	3.74	121.86	147.68	286.35	557.52	590.24	low

Table S3. Total nitrogen TITAN results for amplicon sequence variants (ASV) with >95% purity and 95% reliability, change points (CP), sample frequencies (Freq), z-scores, and percentile distributions of bootstrapped change points. Indicator column denotes high N or low N ASVs that increased or decreased, respectively, with greater TN concentrations.

ASV	CP	Freq	% Freq	Z-Score	0.05	0.1	0.5	0.9	0.95	Indicator
ASV50	404	134	0.48	3.55	289	306	465	1908	2045	high
ASV113	405.5	102	0.36	4.38	405	406	646	1205	2060	high
ASV146	405.5	101	0.36	3.46	358	403	642	825	886	high
ASV324	405.5	37	0.13	3.70	405	406	423	2360	2370	high
ASV248	409.5	44	0.16	3.65	402	404	610	809	836	high
ASV310	413.5	44	0.16	4.01	396	405	426	864	1820	high
ASV373	432	34	0.12	3.85	426	430	574	1885	1908	high
ASV402	560	43	0.15	4.53	519	533	680	1012	1145	high
ASV4	629.5	241	0.86	6.72	455	519	744	809	852	high
ASV75	629.5	152	0.54	3.90	352	617	805	1800	2370	high
ASV219	629.5	117	0.42	4.41	458	625	730	878	1800	high
ASV295	635.5	81	0.29	4.84	553	560	778	1052	1178	high
ASV212	639	81	0.29	3.76	402	410	757	825	902	high
ASV451	639	46	0.16	6.43	479	508	641	851	860	high
ASV109	665	137	0.49	5.61	418	606	665	884	1739	high
ASV365	679.5	90	0.32	10.61	664	667	682	812	851	high
ASV69	685	97	0.35	8.58	665	682	698	861	948	high
ASV145	685	97	0.35	4.68	465	639	713	1868	2045	high
ASV24	689.5	238	0.85	4.36	504	668	716	1290	1330	high
ASV183	696.5	90	0.32	5.69	465	683	698	900	1285	high
ASV305	696.5	17	0.06	3.69	543	685	705	743	809	high
ASV407	696.5	74	0.26	7.15	681	686	697	1291	1440	high
ASV416	696.5	44	0.16	8.54	667	669	787	1315	1440	high
ASV32	704.5	168	0.60	8.18	669	687	698	806	1502	high
ASV237	713	21	0.07	6.89	674	677	785	1135	1290	high
ASV148	721	101	0.36	4.64	576	647	812	1184	1670	high
ASV255	721	66	0.23	3.97	399	402	721	1201	2051	high
ASV72	727.5	103	0.37	4.78	660	669	795	2065	2065	high
ASV353	759	65	0.23	8.34	637	647	772	798	855	high
ASV377	759	49	0.17	7.02	737	754	775	825	841	high
ASV5	771.5	267	0.95	7.50	630	684	771	824	836	high
ASV49	771.5	231	0.82	7.81	626	634	759	823	915	high
ASV200	771.5	83	0.30	4.79	466	519	776	872	879	high
ASV302	771.5	48	0.17	5.60	625	631	774	873	876	high
ASV300	771.5	91	0.32	5.14	504	736	775	886	907	high
ASV335	771.5	32	0.11	3.78	408	410	772	930	2361	high
ASV12	780.5	82	0.29	7.08	558	563	658	781	809	high
ASV124	782.5	66	0.23	6.55	667	672	796	943	1285	high
ASV151	784.5	126	0.45	4.74	432	465	696	1210	1220	high
ASV297	784.5	106	0.38	9.99	688	715	785	823	828	high
ASV388	784.5	112	0.40	6.12	519	617	805	1575	1785	high

ASV397	784.5	31	0.11	7.22	715	760	806	1440	1730	high
ASV54	796.5	204	0.73	6.05	660	665	794	895	1715	high
ASV155	796.5	116	0.41	7.08	595	605	723	972	1061	high
ASV176	796.5	90	0.32	13.24	688	698	798	886	902	high
ASV314	804.5	29	0.10	7.60	694	796	825	1500	1715	high
ASV129	807.5	102	0.36	3.64	365	404	785	2045	2340	high
ASV140	811.5	82	0.29	6.39	620	646	809	1205	1683	high
ASV141	811.5	165	0.59	6.84	674	681	806	1012	1145	high
ASV298	811.5	55	0.20	7.51	705	783	812	1108	1215	high
ASV27	822.5	112	0.40	9.40	687	729	823	855	872	high
ASV432	822.5	28	0.10	5.74	606	776	876	2747	3019	high
ASV197	824.5	144	0.51	11.20	700	780	821	1060	1130	high
ASV111	825	154	0.55	6.11	621	636	823	1195	1211	high
ASV349	829.5	44	0.16	6.48	405	416	830	903	915	high
ASV304	861	42	0.15	4.46	452	458	869	1210	1800	high
ASV71	871.5	156	0.56	5.22	509	647	869	1035	1158	high
ASV198	1010	119	0.42	4.63	615	619	1011	1738	1795	high
ASV55	1025	78	0.28	5.48	464	594	1015	1440	1480	high
ASV319	1025	47	0.17	4.54	432	690	1025	1575	1575	high
ASV392	1035	87	0.31	3.92	410	641	1030	1690	1765	high
ASV114	1130	66	0.23	9.85	710	734	1110	1280	1285	high
ASV268	1215	65	0.23	8.30	598	686	958	1255	1280	high
ASV108	1230	206	0.73	6.13	896	915	1195	1250	1280	high
ASV429	1285	40	0.14	7.28	690	698	1280	1420	1451	high
ASV277	1440	37	0.13	7.33	601	759	1390	1620	1985	high
ASV156	1500	71	0.25	9.09	687	974	1460	1560	1765	high
ASV363	1670	26	0.09	4.96	637	669	1615	1738	2095	high
ASV125	1690	82	0.29	5.26	467	1372	1715	2065	2190	high
ASV231	1715	30	0.11	5.26	464	465	1705	1820	1985	high
ASV238	1765	87	0.31	4.01	282	283	1555	1795	1800	high
ASV289	1820	29	0.10	5.07	633	688	1045	2190	2205	high
ASV215	1840	66	0.23	5.55	573	633	1480	1908	1963	high
ASV364	2370	33	0.12	5.43	403	409	911	2370	2974	high
ASV279	168	34	0.12	4.25	148	168	658	1002	1025	low
ASV421	168	50	0.18	3.95	149	168	374	1130	1135	low
ASV161	178.5	70	0.25	4.63	127	169	263	623	682	low
ASV48	247.5	94	0.33	6.96	202	202	248	315	318	low
ASV308	287	91	0.32	5.71	272	285	313	716	946	low
ASV281	299	50	0.18	8.34	176	179	276	453	465	low
ASV112	315	52	0.19	6.04	214	217	315	480	876	low
ASV259	394	69	0.25	4.26	253	387	504	668	915	low
ASV91	403	160	0.57	5.40	372	402	574	808	836	low
ASV368	405.5	43	0.15	8.47	387	398	467	647	665	low
ASV251	413.5	39	0.14	9.89	173	173	412	422	455	low
ASV325	450.5	81	0.29	6.73	369	371	462	730	772	low
ASV207	459.5	57	0.20	2.99	447	454	663	1100	1390	low
ASV172	462	58	0.21	8.46	355	406	463	594	598	low
ASV239	462	60	0.21	8.28	323	368	457	467	519	low

ASV98	463	83	0.30	9.30	444	450	463	525	589	low
ASV1	464	281	1.00	7.27	451	459	465	655	690	low
ASV15	464	154	0.55	12.15	463	464	467	690	696	low
ASV191	464	41	0.15	8.45	331	378	463	595	602	low
ASV7	464.5	256	0.91	5.17	462	465	510	1290	1300	low
ASV99	464.5	136	0.48	8.76	394	451	465	498	595	low
ASV26	467	182	0.65	5.08	432	462	534	598	826	low
ASV80	467	80	0.28	12.07	432	432	465	647	677	low
ASV152	471	82	0.29	5.35	173	176	463	618	629	low
ASV122	471	32	0.11	7.35	440	457	467	628	680	low
ASV338	492.5	36	0.13	3.87	394	410	496	823	864	low
ASV322	503.5	97	0.35	4.52	464	467	721	1130	1135	low
ASV127	507.5	80	0.28	9.74	365	447	504	626	636	low
ASV293	541.5	37	0.13	4.97	465	518	564	730	790	low
ASV107	545	102	0.36	6.88	432	462	542	698	713	low
ASV287	560	17	0.06	4.18	331	354	558	688	690	low
ASV434	561.5	18	0.06	6.22	355	406	464	573	688	low
ASV355	565	56	0.20	3.13	168	169	565	1145	1280	low
ASV201	568.5	63	0.22	4.24	217	378	519	721	729	low
ASV272	568.5	35	0.12	8.93	403	416	549	571	574	low
ASV132	571	135	0.48	5.53	190	194	461	577	581	low
ASV356	578	41	0.15	9.59	401	406	558	598	608	low
ASV46	584.5	189	0.67	6.42	398	402	519	589	601	low
ASV260	588.5	52	0.19	3.36	305	409	590	906	983	low
ASV406	588.5	36	0.13	5.28	462	519	555	628	637	low
ASV30	591	230	0.82	4.15	462	465	591	1256	1295	low
ASV358	593.5	100	0.36	5.21	434	458	559	728	733	low
ASV273	616.5	41	0.15	5.02	168	168	607	743	956	low
ASV234	629.5	59	0.21	5.71	335	362	621	682	686	low
ASV86	654.5	87	0.31	6.35	414	420	655	690	772	low
ASV87	668	174	0.62	4.82	464	465	583	730	875	low
ASV82	669	94	0.33	5.62	335	406	673	776	957	low
ASV20	677	172	0.61	7.68	418	432	569	696	705	low
ASV213	677	83	0.30	4.32	385	402	677	690	721	low
ASV290	681.5	27	0.10	5.10	168	169	676	781	783	low
ASV246	687.5	93	0.33	8.30	413	426	618	760	773	low
ASV382	687.5	26	0.09	4.55	319	319	585	703	825	low
ASV311	689.5	64	0.23	7.27	402	467	688	706	742	low
ASV9	696.5	231	0.82	5.13	456	466	690	772	780	low
ASV14	696.5	236	0.84	3.77	353	389	505	1187	1280	low
ASV115	715	41	0.15	4.57	399	612	715	946	958	low
ASV150	721	86	0.31	4.05	462	465	725	806	1230	low
ASV3	759	224	0.80	6.35	688	705	743	797	992	low
ASV42	771.5	171	0.61	7.69	451	457	591	773	788	low
ASV387	771.5	82	0.29	3.45	272	462	772	1230	1285	low
ASV52	991.5	118	0.42	4.36	463	640	991	1285	1290	low
ASV185	991.5	59	0.21	3.79	217	466	939	1025	1040	low

ASV10	1195	119	0.42	4.04	689	742	896	1215	1225	low

Table S4. Amplicon sequence variants identified as having random forest models with R^2 values > 0 in gradient forest analysis.

ASV	R2
ASV20	0.57
ASV12	0.55
ASV353	0.50
ASV203	0.43
ASV15	0.42
ASV146	0.40
ASV354	0.37
ASV39	0.36
ASV4	0.35
ASV373	0.34
ASV80	0.34
ASV297	0.34
ASV310	0.33
ASV42	0.33
ASV22	0.32
ASV323	0.32
ASV287	0.31
ASV140	0.31
ASV26	0.30
ASV6	0.30
ASV437	0.29
ASV213	0.28
ASV85	0.28
ASV75	0.28
ASV110	0.27
ASV402	0.27
ASV97	0.27
ASV208	0.26
ASV1	0.26
ASV127	0.26
ASV374	0.26
ASV201	0.25
ASV371	0.25
ASV9	0.25
ASV7	0.25
ASV11	0.25
ASV301	0.25
ASV113	0.24
ASV161	0.24
ASV418	0.24
ASV49	0.24
ASV293	0.24

ASV212	0.23
ASV107	0.23
ASV13	0.23
ASV74	0.23
ASV200	0.22
ASV94	0.22
ASV25	0.22
ASV88	0.22
ASV5	0.22
ASV324	0.22
ASV68	0.21
ASV145	0.21
ASV266	0.21
ASV50	0.21
ASV32	0.21
ASV325	0.21
ASV246	0.21
ASV184	0.20
ASV60	0.20
ASV41	0.20
ASV364	0.20
ASV416	0.20
ASV98	0.19
ASV129	0.19
ASV142	0.19
ASV209	0.18
ASV185	0.18
ASV360	0.18
ASV228	0.18
ASV295	0.18
ASV99	0.17
ASV37	0.17
ASV290	0.17
ASV309	0.17
ASV69	0.17
ASV27	0.16
ASV95	0.16
ASV451	0.16
ASV304	0.16
ASV90	0.16
ASV150	0.16
ASV335	0.15
ASV24	0.15
ASV176	0.15
ASV92	0.15
ASV272	0.15
ASV178	0.15
ASV332	0.15

ASV87	0.14
ASV191	0.14
ASV172	0.14
ASV316	0.14
ASV219	0.14
ASV162	0.14
ASV368	0.14
ASV311	0.14
ASV165	0.14
ASV248	0.14
ASV302	0.14
ASV314	0.14
ASV151	0.14
ASV197	0.13
ASV174	0.13
ASV23	0.13
ASV124	0.13
ASV58	0.13
ASV72	0.13
ASV53	0.13
ASV183	0.13
ASV194	0.13
ASV78	0.13
ASV86	0.12
ASV30	0.12
ASV247	0.12
ASV168	0.12
ASV328	0.12
ASV351	0.12
ASV291	0.12
ASV149	0.12
ASV83	0.12
ASV89	0.12
ASV237	0.12
ASV51	0.11
ASV265	0.11
ASV463	0.11
ASV33	0.11
ASV128	0.11
ASV133	0.11
ASV71	0.11
ASV365	0.11
ASV340	0.11
ASV442	0.11
ASV277	0.11
ASV376	0.10
ASV91	0.10
ASV408	0.10

ASV109	0.10
ASV144	0.10
ASV299	0.10
ASV62	0.10
ASV137	0.10
ASV234	0.10
ASV220	0.10
ASV119	0.10
ASV211	0.10
ASV180	0.10
ASV66	0.10
ASV108	0.10
ASV271	0.10
ASV76	0.10
ASV17	0.09
ASV55	0.09
ASV101	0.09
ASV61	0.09
ASV34	0.09
ASV14	0.09
ASV253	0.09
ASV81	0.09
ASV357	0.09
ASV300	0.09
ASV281	0.09
ASV377	0.08
ASV54	0.08
ASV189	0.08
ASV303	0.08
ASV31	0.08
ASV238	0.08
ASV259	0.08
ASV156	0.08
ASV43	0.08
ASV116	0.08
ASV198	0.07
ASV434	0.07
ASV245	0.07
ASV65	0.07
ASV274	0.07
ASV132	0.07
ASV404	0.07
ASV131	0.07
ASV170	0.07
ASV29	0.07
ASV70	0.07
ASV193	0.06
ASV46	0.06

ASV225	0.06
ASV285	0.06
ASV152	0.06
ASV391	0.06
ASV398	0.06
ASV18	0.06
ASV153	0.06
ASV121	0.06
ASV282	0.06
ASV341	0.06
ASV139	0.06
ASV284	0.06
ASV298	0.06
ASV250	0.06
ASV322	0.06
ASV349	0.06
ASV154	0.05
ASV28	0.05
ASV361	0.05
ASV370	0.05
ASV222	0.05
ASV317	0.05
ASV215	0.05
ASV96	0.05
ASV73	0.05
ASV401	0.05
ASV268	0.05
ASV230	0.05
ASV294	0.05
ASV407	0.04
ASV362	0.04
ASV321	0.04
ASV235	0.04
ASV2	0.04
ASV67	0.04
ASV138	0.04
ASV397	0.04
ASV338	0.04
ASV432	0.04
ASV336	0.03
ASV343	0.03
ASV231	0.03
ASV141	0.03
ASV177	0.03
ASV167	0.03
ASV262	0.03
ASV485	0.03
ASV35	0.03

ASV125	0.03
ASV379	0.03
ASV175	0.03
ASV395	0.02
ASV190	0.02
ASV19	0.02
ASV367	0.02
ASV334	0.02
ASV405	0.02
ASV93	0.02
ASV440	0.02
ASV206	0.02
ASV148	0.02
ASV204	0.02
ASV40	0.02
ASV240	0.02
ASV166	0.02
ASV412	0.02
ASV378	0.02
ASV242	0.02
ASV358	0.02
ASV112	0.02
ASV429	0.01
ASV136	0.01
ASV387	0.01
ASV21	0.01
ASV164	0.01
ASV239	0.01
ASV296	0.01
ASV267	0.01
ASV102	0.01
ASV44	0.01
ASV330	0.01
ASV171	0.01
ASV350	0.01
ASV218	0.01
ASV252	0.00*
ASV117	0.00*
ASV115	0.00*
ASV64	0.00*
ASV352	0.00*
ASV337	0.00*
ASV275	0.00*
ASV169	0.00*

* Zero values are due to rounding.

sequence_identifier	sequence_score	identity	quality	lca_tax_rdp	lca_tax_slv
ASV001	0	100	100	Bacteria;"Proteobacteria";Alphaproteobacteria;Rhodobacterales;Rhodobacteraceae;	Bacteria;Proteobacteria;Alphaproteobacteria;Rhodobacterales;Rhodobacteraceae;
ASV002	0	100	100	Bacteria;Firmicutes;Bacilli;Bacillales;Bacillales_Incertae Sedis XII;Exiguobacterium;	Bacteria;Firmicutes;Bacilli;Exiguobacterales;Exiguobacteraceae;Exiguobacterium;
ASV003	0	100	100	Bacteria;"Proteobacteria";Gammaproteobacteria;Pseudomonadales;Moraxellaceae;Acinetobacter;	Bacteria;Proteobacteria;Gammaproteobacteria;Pseudomonadales;Moraxellaceae;Acinetobacter;
ASV004	0.994276	99.5868	99	Bacteria;"Proteobacteria";Alphaproteobacteria;Rhizobiales;unclassified_Rhizobiales;	Bacteria;Proteobacteria;Alphaproteobacteria;Rhizobiales;Rhizobiales Incertae Sedis;uncultured;
ASV005	0.978057	91.7355	97	Bacteria;Cyanobacteria/Chloroplast;Chloroplast;Chloroplast;Bacillariophyta;	Bacteria;Cyanobacteria;Cyanobacteriia;Chloroplast;
ASV006	0	100	100	Bacteria;"Proteobacteria";Alphaproteobacteria;Rhizobiales;unclassified_Rhizobiales;	Bacteria;Proteobacteria;Alphaproteobacteria;Rhizobiales;Rhizobiales Incertae Sedis;uncultured;
ASV007	0.994294	98.7603	99	Bacteria;"Proteobacteria";Alphaproteobacteria;Rhizobiales;unclassified_Rhizobiales;	Bacteria;Proteobacteria;Alphaproteobacteria;Rhizobiales;Rhizobiales Incertae Sedis;uncultured;
ASV008	0.994429	99.5868	99	Bacteria;"Proteobacteria";Alphaproteobacteria;Rhodobacterales;Rhodobacteraceae;	Bacteria;Proteobacteria;Alphaproteobacteria;Rhodobacterales;Rhodobacteraceae;Rhodobacter;
ASV009	0	100	100	Bacteria;"Proteobacteria";Alphaproteobacteria;Rhodobacterales;Rhodobacteraceae;	Bacteria;Proteobacteria;Alphaproteobacteria;Rhodobacterales;Rhodobacteraceae;Rhodobacter;
ASV010	0.994277	95.8848	99	Bacteria;"Proteobacteria";Gammaproteobacteria;Pseudomonadales;Moraxellaceae;	Bacteria;Proteobacteria;Gammaproteobacteria;Pseudomonadales;Moraxellaceae;
ASV011	0.994426	99.5868	99	Bacteria;"Proteobacteria";Alphaproteobacteria;Rhodobacterales;Rhodobacteraceae;	Bacteria;Proteobacteria;Alphaproteobacteria;Rhodobacterales;Rhodobacteraceae;
ASV012	0.977152	93.3884	97	Bacteria;Cyanobacteria/Chloroplast;Chloroplast;Chloroplast;Bacillariophyta;	Bacteria;Cyanobacteria;Cyanobacteriia;Chloroplast;
ASV013	0	100	100	Bacteria;"Proteobacteria";Alphaproteobacteria;Sphingomonadales;Erythrobacteraceae;Porphyrobacter;	Bacteria;Proteobacteria;Alphaproteobacteria;Sphingomonadales;Sphingomonadaceae;Porphyrobacter;
ASV014	0	100	100	Bacteria;"Proteobacteria";Alphaproteobacteria;Rhodobacterales;Rhodobacteraceae;	Bacteria;Proteobacteria;Alphaproteobacteria;Rhodobacterales;Rhodobacteraceae;
ASV015	0	100	100	Bacteria;"Proteobacteria";Alphaproteobacteria;Rhodobacterales;Rhodobacteraceae;	Bacteria;Proteobacteria;Alphaproteobacteria;Rhodobacterales;Rhodobacteraceae;
ASV016	0	100	100	Bacteria;Firmicutes;Bacilli;Bacillales;Bacillales_Incertae Sedis XII;Exiguobacterium;	Bacteria;Firmicutes;Bacilli;Exiguobacterales;Exiguobacteraceae;Exiguobacterium;
ASV017	0.981446	95.4545	98	Bacteria;"Proteobacteria";Alphaproteobacteria;	Bacteria;Proteobacteria;Alphaproteobacteria;Rhizobiales;Rhizobiales Incertae Sedis;uncultured;
ASV018	0	100	100	Bacteria;"Proteobacteria";Alphaproteobacteria;Sphingomonadales;Sphingomonadaceae;Sphingorhabdus;	Bacteria;Proteobacteria;Alphaproteobacteria;Sphingomonadales;Sphingomonadaceae;Sphingorhabdus;
ASV019	0	100	100	Bacteria;"Proteobacteria";Gammaproteobacteria;Xanthomonadales;Sinobacteraceae;Povalibacter;	Bacteria;Proteobacteria;Gammaproteobacteria;Steroidobacterales;Steroidobacteraceae;uncultured;
ASV020	0.96929	97.5309	96	Bacteria;"Proteobacteria";Alphaproteobacteria;Rhodobacterales;Rhodobacteraceae;	Bacteria;Proteobacteria;Alphaproteobacteria;Rhodobacterales;Rhodobacteraceae;Rhodobacter;

ASV021	0.988724	98.3471	98	Bacteria;Cyanobacteria/Chloroplast;Chloroplast;Chloroplast;Bacillariophyta;	Bacteria;Cyanobacteria;Cyanobacteriia;Chloroplast;
ASV022	0.994232	98.7603	99	Bacteria;"Proteobacteria";Alphaproteobacteria;Rhizobiales;Rhizobiales_incertae_sedis;Alsobacter;	Bacteria;Proteobacteria;Alphaproteobacteria;Rhizobiales;
ASV023	0.994369	99.5868	99	Bacteria;"Proteobacteria";Alphaproteobacteria;Rhizobiales;Rhodobiaceae;	Bacteria;Proteobacteria;Alphaproteobacteria;Rhizobiales;Methyloligellaceae;uncultured;
ASV024	0	100	100	Bacteria;"Proteobacteria";Alphaproteobacteria;Sphingomonadales;Erythrobacteraceae;Altererythrobacter;	Bacteria;Proteobacteria;Alphaproteobacteria;Sphingomonadales;Sphingomonadaceae;
ASV025	0.994375	99.5868	99	Bacteria;"Proteobacteria";Alphaproteobacteria;Rhodobacterales;Rhodobacteraceae;unclassified_Rhodobacteraceae;	Bacteria;Proteobacteria;Alphaproteobacteria;Rhodobacterales;Rhodobacteraceae;
ASV026	0.991336	99.5868	99	Bacteria;"Proteobacteria";Alphaproteobacteria;Rhizobiales;Hyphomicrobiaceae;Hyphomicrobium;	Bacteria;Proteobacteria;Alphaproteobacteria;Rhizobiales;Hyphomicrobiaceae;Hyphomicrobium;
ASV027	0.994437	98.7654	99	Bacteria;"Proteobacteria";	Bacteria;Proteobacteria;Gammaproteobacteria;Burkholderiales;Comamonadaceae;
ASV028	0	100	100	Bacteria;"Proteobacteria";Alphaproteobacteria;Rhizobiales;	Bacteria;Proteobacteria;Alphaproteobacteria;Rhizobiales;Beijerinckiaceae;
ASV029	0.994281	99.5868	99	Bacteria;Cyanobacteria/Chloroplast;Chloroplast;Chloroplast;Bacillariophyta;	Bacteria;Cyanobacteria;Cyanobacteriia;Chloroplast;
ASV030	0.994301	98.7603	99	Bacteria;"Actinobacteria";Actinobacteria;Actinobacteridae;Actinomycetales;Frankineae;Sporichthyaceae;Sporichthya;	Bacteria;Actinobacteriota;Actinobacteria;Frankiales;Sporichthyaceae;
ASV031	0	100	100	Bacteria;"Proteobacteria";Gammaproteobacteria;Pseudomonadales;Moraxellaceae;Acinetobacter;	Bacteria;Proteobacteria;Gammaproteobacteria;Pseudomonadales;Moraxellaceae;Acinetobacter;
ASV032	0	100	100	Bacteria;"Proteobacteria";Alphaproteobacteria;Sphingomonadales;Sphingomonadaceae;Sphingomonas;	Bacteria;Proteobacteria;Alphaproteobacteria;Sphingomonadales;Sphingomonadaceae;Sphingomonas;
ASV033	0	100	100	Bacteria;"Proteobacteria";Alphaproteobacteria;Rhizobiales;Bradyrhizobiaceae;Bradyrhizobium;	Bacteria;Proteobacteria;Alphaproteobacteria;Rhizobiales;Xanthobacteraceae;Bradyrhizobium;
ASV034	0	100	100	Bacteria;"Proteobacteria";Gammaproteobacteria;Xanthomonadales;Xanthomonadaceae;Thermomonas;	Bacteria;Proteobacteria;Gammaproteobacteria;Xanthomonadales;Xanthomonadaceae;Thermomonas;
ASV035	0	100	100	Bacteria;"Proteobacteria";Alphaproteobacteria;Rhizobiales;Hyphomicrobiaceae;Devosia;	Bacteria;Proteobacteria;Alphaproteobacteria;Rhizobiales;Devosiaceae;Devosia;
ASV036	0	100	100	Bacteria;"Proteobacteria";Gammaproteobacteria;Pseudomonadales;Moraxellaceae;Acinetobacter;	Bacteria;Proteobacteria;Gammaproteobacteria;Pseudomonadales;Moraxellaceae;Acinetobacter;
ASV037	0	100	100	Bacteria;"Proteobacteria";Betaproteobacteria;Burkholderiales;	Bacteria;Proteobacteria;Gammaproteobacteria;Burkholderiales;Comamonadaceae;
ASV038	0.994349	99.5868	99	Bacteria;"Proteobacteria";Alphaproteobacteria;Rhizobiales;Xanthobacteraceae;	Bacteria;Proteobacteria;Alphaproteobacteria;Rhizobiales;Xanthobacteraceae;
ASV039	0.994319	97.1193	99	Bacteria;"Proteobacteria";Alphaproteobacteria;Rhodobacterales;Rhodobacteraceae;	Bacteria;Proteobacteria;Alphaproteobacteria;Rhodobacterales;Rhodobacteraceae;
ASV040	0	100	100	Bacteria;"Proteobacteria";Gammaproteobacteria;Xanthomonadales;Xanthomonadaceae;Lysobacter;	Bacteria;Proteobacteria;Gammaproteobacteria;Xanthomonadales;Xanthomonadaceae;Lysobacter;
ASV041	0.994347	97.9424	99	Bacteria;"Proteobacteria";Alphaproteobacteria;Rhizobiales;	Bacteria;Proteobacteria;Alphaproteobacteria;Rhizobiales;Beijerinckiaceae;
ASV042	0	100	100	Bacteria;"Proteobacteria";Alphaproteobacteria;Sphingomonadales;Sphingomonadaceae;	Bacteria;Proteobacteria;Alphaproteobacteria;Sphingomonadales;Sphingomonadaceae;

ASV043	0.987799	95.0413	98	Bacteria;"Proteobacteria";Betaproteobacteria;Burkholderiales;unclassified_Burkholderiales;	Bacteria;Proteobacteria;Gammaproteobacteria;Burkholderiales;Sutterellaceae;uncultured;
ASV044	0	100	100	Bacteria;"Proteobacteria";Alphaproteobacteria;Sphingomonadales;Sphingomonadaceae;Novosphingobium;	Bacteria;Proteobacteria;Alphaproteobacteria;Sphingomonadales;Sphingomonadaceae;Novosphingobium;
ASV045	0.994221	99.1736	99	Bacteria;"Proteobacteria";Gammaproteobacteria;Xanthomonadales;Sinobacteraceae;Povalibacter;	Bacteria;Proteobacteria;Gammaproteobacteria;Steroidobacterales;Steroidobacteraceae;uncultured;
ASV046	0	100	100	Bacteria;"Proteobacteria";Gammaproteobacteria;Xanthomonadales;Xanthomonadaceae;Arenimonas;	Bacteria;Proteobacteria;Gammaproteobacteria;Xanthomonadales;Xanthomonadaceae;Arenimonas;
ASV047	0.994432	99.5868	99	Bacteria;"Proteobacteria";Gammaproteobacteria;Pseudomonadales;Pseudomonadaceae;Rhizobacter;	Bacteria;Proteobacteria;Gammaproteobacteria;Burkholderiales;Comamonadaceae;
ASV048	0	100	100	Bacteria;"Proteobacteria";Gammaproteobacteria;Pseudomonadales;Moraxellaceae;Acinetobacter;	Bacteria;Proteobacteria;Gammaproteobacteria;Pseudomonadales;Moraxellaceae;Acinetobacter;
ASV049	0.835037	72.7273	83	Unclassified;	Bacteria; Proteobacteria; Alphaproteobacteria; Rickettsiales;
ASV050	0.994371	99.5868	99	Bacteria;"Proteobacteria";Alphaproteobacteria;Rhizobiales;unclassified_Rhizobiales;	Bacteria;Proteobacteria;Alphaproteobacteria;Rhizobiales;Xanthobacteraceae;
ASV051	0	100	100	Bacteria;"Proteobacteria";Alphaproteobacteria;Rhizobiales;Xanthobacteraceae;Xanthobacter;	Bacteria;Proteobacteria;Alphaproteobacteria;Rhizobiales;Xanthobacteraceae;Xanthobacter;
ASV052	0	100	100	Bacteria;Cyanobacteria/Chloroplast;Chloroplast;Chloroplast;Bacillariophyta;	Bacteria;Cyanobacteria;Cyanobacteriia;Chloroplast;
ASV053	0	100	100	Bacteria;"Proteobacteria";Alphaproteobacteria;Sphingomonadales;Sphingomonadaceae;	Bacteria;Proteobacteria;Alphaproteobacteria;Sphingomonadales;Sphingomonadaceae;uncultured;
ASV054	0	100	100	Bacteria;"Actinobacteria";Actinobacteria;Actinobacteridae;Actinomycetales;Micrococcineae;Micrococcaceae;Arthrobacter;	Bacteria;Actinobacteriota;Actinobacteria;Micrococcales;Micrococcaceae;
ASV055	0.994401	99.5868	99	Bacteria;"Proteobacteria";Alphaproteobacteria;Sphingomonadales;Erythrobacteraceae;	Bacteria;Proteobacteria;Alphaproteobacteria;Sphingomonadales;Sphingomonadaceae;
ASV056	0	100	100	Bacteria;"Proteobacteria";Alphaproteobacteria;Sphingomonadales;Sphingomonadaceae;	Bacteria;Proteobacteria;Alphaproteobacteria;Sphingomonadales;Sphingomonadaceae;Sphingomonas;
ASV057	0.994475	99.5868	99	Bacteria;"Bacteroidetes";Flavobacteriia;"Flavobacteriales";Flavobacteriaceae;Chryseobacterium;	Bacteria;Bacteroidota;Bacteroidia;Flavobacteriales;Weeksellaceae;Chryseobacterium;
ASV058	0.994415	99.5868	99	Bacteria;"Proteobacteria";Betaproteobacteria;Burkholderiales;Burkholderiales_incertae_sedis;	Bacteria;Proteobacteria;Gammaproteobacteria;Burkholderiales;Comamonadaceae;
ASV059	0.994443	99.5868	99	Bacteria;Cyanobacteria/Chloroplast;Cyanobacteria;	Bacteria;Cyanobacteria;Cyanobacteriia;Cyanobacteriales;Phormidiaceae;Tychonema CCAP 1459-11B;
ASV060	0.981966	98.7603	98	Bacteria;"Proteobacteria";Alphaproteobacteria;Rhodobacterales;Rhodobacteraceae;	Bacteria;Proteobacteria;Alphaproteobacteria;Rhodobacterales;Rhodobacteraceae;
ASV061	0	100	100	Bacteria;"Proteobacteria";Betaproteobacteria;Burkholderiales;Burkholderiales_incertae_sedis;unclassified_Burkholderiales_incertae_sedis;	Bacteria;Proteobacteria;Gammaproteobacteria;Burkholderiales;Comamonadaceae;
ASV062	0	100	100	Bacteria;"Proteobacteria";Alphaproteobacteria;Sphingomonadales;Sphingomonadaceae;Novosphingobium;	Bacteria;Proteobacteria;Alphaproteobacteria;Sphingomonadales;Sphingomonadaceae;Novosphingobium;
ASV063	0.994379	98.7603	99	Bacteria;"Proteobacteria";Alphaproteobacteria;Caulobacterales;Caulobacteraceae;Brevundimonas;	Bacteria;Proteobacteria;Alphaproteobacteria;Caulobacterales;Caulobacteraceae;Brevundimonas;
ASV064	0	100	100	Bacteria;Firmicutes;Bacilli;Bacillales;	Bacteria;Firmicutes;Bacilli;Bacillales;

ASV065	0	100	100	Bacteria;"Proteobacteria";Alphaproteobacteria;Caulobacterales;Caulobacteraceae;Brevundimonas;	Bacteria;Proteobacteria;Alphaproteobacteria;Caulobacterales;Caulobacteraceae;Brevundimonas;
ASV066	0.994304	99.5868	99	Bacteria;"Proteobacteria";Alphaproteobacteria;Caulobacterales;Caulobacteraceae;Phenylobacterium;	Bacteria;Proteobacteria;Alphaproteobacteria;Caulobacterales;Caulobacteraceae;Phenylobacterium;
ASV067	0	100	100	Bacteria;"Proteobacteria";Betaproteobacteria;Burkholderiales;	Bacteria;Proteobacteria;Gammaproteobacteria;Burkholderiales;Comamonadaceae;
ASV068	0.994561	98.3539	99	Bacteria;"Proteobacteria";Gammaproteobacteria;Xanthomonadales;Sinobacteraceae;Povalibacter;	Bacteria;Proteobacteria;Gammaproteobacteria;Steroidobacterales;Steroidobacteraceae;uncultured;
ASV069	0.994355	99.1736	99	Bacteria;"Proteobacteria";Alphaproteobacteria;Sphingomonadales;Sphingomonadaceae;	Bacteria;Proteobacteria;Alphaproteobacteria;Sphingomonadales;Sphingomonadaceae;
ASV070	0.994413	99.177	99	Bacteria;"Proteobacteria";Gammaproteobacteria;Xanthomonadales;Xanthomonadaceae;Lysobacter;	Bacteria;Proteobacteria;Gammaproteobacteria;Xanthomonadales;Xanthomonadaceae;Lysobacter;
ASV071	0.990174	91.358	99	Unclassified;	Bacteria; Cyanobacteria; Oscillatoriophycideae; Chroococcales; Xenococcaceae;
ASV072	0	100	100	Bacteria;"Actinobacteria";Actinobacteria;Actinobacteridae;Actinomycetales;Micrococcineae;Intrasporangiaceae;	Bacteria;Actinobacteriota;Actinobacteria;Micrococcales;Intrasporangiaceae;
ASV073	0.994226	98.3471	99	Bacteria;"Actinobacteria";Actinobacteria;Actinobacteridae;Actinomycetales;	Bacteria;Actinobacteriota;Actinobacteria;PeM15;
ASV074	0.994362	98.3471	99	Bacteria;"Proteobacteria";Alphaproteobacteria;Sphingomonadales;Sphingomonadaceae;	Bacteria;Proteobacteria;Alphaproteobacteria;Sphingomonadales;Sphingomonadaceae;
ASV075	0.994306	98.3471	99	Bacteria;"Proteobacteria";Alphaproteobacteria;Rhizobiales;Rhizobiales_incertae_sedis;Alsobacter;	Bacteria;Proteobacteria;Alphaproteobacteria;Rhizobiales;
ASV076	0.994391	99.5868	99	Bacteria;"Proteobacteria";Alphaproteobacteria;Rhizobiales;	Bacteria;Proteobacteria;Alphaproteobacteria;Rhizobiales;Xanthobacteraceae;
ASV077	0	100	100	Bacteria;"Proteobacteria";Gammaproteobacteria;Pseudomonadales;Pseudomonadaceae;Pseudomonas;	Bacteria;Proteobacteria;Gammaproteobacteria;Pseudomonadales;Pseudomonadaceae;Pseudomonas;
ASV078	0.994432	99.5868	99	Bacteria;"Proteobacteria";Alphaproteobacteria;Rhodobacterales;Rhodobacteraceae;	Bacteria;Proteobacteria;Alphaproteobacteria;Rhodobacterales;Rhodobacteraceae;
ASV079	0	100	100	Bacteria;Firmicutes;Bacilli;Bacillales;Bacillaceae 1;	Bacteria;Firmicutes;Bacilli;Bacillales;Bacillaceae;Bacillus;
ASV080	0.981893	99.1736	98	Bacteria;"Proteobacteria";Alphaproteobacteria;Rhodobacterales;Rhodobacteraceae;	Bacteria;Proteobacteria;Alphaproteobacteria;Rhodobacterales;Rhodobacteraceae;
ASV081	0	100	100	Bacteria;"Proteobacteria";Gammaproteobacteria;	Bacteria;Proteobacteria;Gammaproteobacteria;Pseudomonadales;Halieaceae;OM60(NOR5) clade;
ASV082	0	100	100	Bacteria;"Proteobacteria";Alphaproteobacteria;Sphingomonadales;Sphingomonadaceae;Novosphingobium;	Bacteria;Proteobacteria;Alphaproteobacteria;Sphingomonadales;Sphingomonadaceae;Novosphingobium;
ASV083	0	100	100	Bacteria;"Proteobacteria";Gammaproteobacteria;Xanthomonadales;Sinobacteraceae;Povalibacter;	Bacteria;Proteobacteria;Gammaproteobacteria;Steroidobacterales;Steroidobacteraceae;uncultured;
ASV084	0.98339	99.5885	98	Bacteria;Cyanobacteria/Chloroplast;Chloroplast;Chloroplast;Bacillariophyta;	Bacteria;Cyanobacteria;Cyanobacteriia;Chloroplast;
ASV085	0	100	100	Bacteria;"Actinobacteria";Actinobacteria;Actinobacteridae;Actinomycetales;	Bacteria;Actinobacteriota;Actinobacteria;PeM15;
ASV086	0.994456	99.5868	99	Bacteria;"Actinobacteria";Actinobacteria;Actinobacteridae;Actinomycetales;Micrococcineae;Microbacteriaceae;	Bacteria;Actinobacteriota;Actinobacteria;Micrococcales;Microbacteriaceae;

ASV087	0.981885	96.6942	98	Bacteria;"Proteobacteria";Alphaproteobacteria;Rhizobiales;	Bacteria;Proteobacteria;Alphaproteobacteria;Rhizobiales;Rhizobiaceae;
ASV088	0.988007	99.1736	98	Bacteria;"Proteobacteria";Alphaproteobacteria;Sphingomonadales;Sphingomonadaceae;	Bacteria;Proteobacteria;Alphaproteobacteria;Sphingomonadales;Sphingomonadaceae;Sandaracinobacter;
ASV089	0.981363	96.6942	98	Bacteria;"Bacteroidetes";Sphingobacteriia;"Sphingobacteriales";Chitinophagaceae;	Bacteria;Bacteroidota;Bacteroidia;Chitinophagales;Chitinophagaceae;
ASV090	0	100	100	Bacteria;"Proteobacteria";Betaproteobacteria;Burkholderiales;Comamonadaceae;	Bacteria;Proteobacteria;Gammaproteobacteria;Burkholderiales;Comamonadaceae;Ramlibacter;
ASV091	0.988063	95.8678	98	Bacteria;"Proteobacteria";Betaproteobacteria;	Bacteria;Proteobacteria;Gammaproteobacteria;Burkholderiales;
ASV092	0	100	100	Bacteria;"Proteobacteria";Alphaproteobacteria;Rhizobiales;Rhodobiaceae;	Bacteria;Proteobacteria;Alphaproteobacteria;Rhizobiales;Methyloligellaceae;uncultured;
ASV093	0.994379	99.1736	99	Bacteria;"Proteobacteria";Alphaproteobacteria;Sphingomonadales;Sphingomonadaceae;	Bacteria;Proteobacteria;Alphaproteobacteria;Sphingomonadales;Sphingomonadaceae;
ASV094	0.988056	99.5868	98	Bacteria;"Proteobacteria";Alphaproteobacteria;Rhizobiales;Hyphomicrobiaceae;Hyphomicrobium;	Bacteria;Proteobacteria;Alphaproteobacteria;Rhizobiales;Hyphomicrobiaceae;Hyphomicrobium;
ASV095	0.989238	95.0413	98	Bacteria;"Proteobacteria";Alphaproteobacteria;Rhizobiales;unclassified_Rhizobiales;	Bacteria;Proteobacteria;Alphaproteobacteria;Rhizobiales;Rhizobiales Incertae Sedis;uncultured;
ASV096	0.9944	99.5868	99	Bacteria;"Proteobacteria";Alphaproteobacteria;Sphingomonadales;Sphingomonadaceae;	Bacteria;Proteobacteria;Alphaproteobacteria;Sphingomonadales;Sphingomonadaceae;
ASV097	0	100	100	Bacteria;"Actinobacteria";Actinobacteria;Actinobacteridae;Actinomycetales;Micrococcineae;Microbacteriaceae;unclassified_Microbacteriaceae;	Bacteria;Actinobacteriota;Actinobacteria;Micrococcales;Microbacteriaceae;Aurantimicrobium;
ASV098	0.994136	98.3607	99	Bacteria;"Verrucomicrobia";Verrucomicrobiae;Verrucomicrobiales;Verrucomicrobiaceae;Luteolibacter;	Bacteria;Verrucomicrobiota;Verrucomicrobiae;Verrucomicrobiales;Rubritaleaceae;Luteolibacter;
ASV099	0.994153	98.7654	99	Bacteria;"Verrucomicrobia";Verrucomicrobiae;Verrucomicrobiales;Verrucomicrobiaceae;Luteolibacter;	Bacteria;Verrucomicrobiota;Verrucomicrobiae;Verrucomicrobiales;Rubritaleaceae;Luteolibacter;
ASV100	0	100	100	Bacteria;Firmicutes;Bacilli;Bacillales;Bacillaceae 1;Bacillus;	Bacteria;Firmicutes;Bacilli;Bacillales;Bacillaceae;Bacillus;
ASV101	0.994235	97.5207	99	Bacteria;"Proteobacteria";Alphaproteobacteria;Rhizobiales;unclassified_Rhizobiales;	Bacteria;Proteobacteria;Alphaproteobacteria;Rhizobiales;
ASV102	0.994406	98.3539	99	Bacteria;Firmicutes;Bacilli;Bacillales;Bacillales_Incertae Sedis XII;Exiguobacterium;	Bacteria;Firmicutes;Bacilli;Exiguobacterales;Exiguobacteraceae;Exiguobacterium;
ASV103	0.975895	98.7603	97	Bacteria;"Proteobacteria";Alphaproteobacteria;Rhizobiales;Brucellaceae;Brucella;	Bacteria;Proteobacteria;Alphaproteobacteria;Rhizobiales;Rhizobiaceae;
ASV104	0	100	100	Bacteria;"Proteobacteria";Gammaproteobacteria;"Enterobacteriales";Enterobacteriaceae;	Bacteria;Proteobacteria;Gammaproteobacteria;Enterobacterales;Enterobacteriaceae;
ASV105	0	100	100	Bacteria;"Proteobacteria";Alphaproteobacteria;Rhizobiales;Rhizobiaceae;Rhizobium;	Bacteria;Proteobacteria;Alphaproteobacteria;Rhizobiales;Rhizobiaceae;Allorhizobium-Neorhizobium-Pararhizobium-Rhizobium;
ASV106	0	100	100	Bacteria;"Bacteroidetes";Sphingobacteriia;"Sphingobacteriales";Chitinophagaceae;	Bacteria;Bacteroidota;Bacteroidia;Chitinophagales;Chitinophagaceae;
ASV107	0	100	100	Bacteria;"Proteobacteria";Gammaproteobacteria;Xanthomonadales;Xanthomonadaceae;Arenimonas;	Bacteria;Proteobacteria;Gammaproteobacteria;Xanthomonadales;Xanthomonadaceae;Arenimonas;
ASV108	0.972711	92.9752	97	Bacteria;"Proteobacteria";Gammaproteobacteria;Xanthomonadales;Sinobacteraceae;Povalibacter;	Bacteria;Proteobacteria;Gammaproteobacteria;

ASV109	0.987991	98.3471	98	Bacteria;"Proteobacteria";Alphaproteobacteria;Rhizobiales;Phyllobacteriaceae;	Bacteria;Proteobacteria;Alphaproteobacteria;
ASV110	0.98782	96.6942	98	Bacteria;"Proteobacteria";Gammaproteobacteria;Xanthomonadales;Xanthomonadaceae;	Bacteria;Proteobacteria;Gammaproteobacteria;Xanthomonadales;Rhodanobacteraceae;
ASV111	0.979283	97.5207	97	Bacteria;"Proteobacteria";Alphaproteobacteria;Sphingomonadales;	Bacteria;Proteobacteria;Alphaproteobacteria;Sphingomonadales;Sphingomonadaceae;
ASV112	0	100	100	Bacteria;"Proteobacteria";Gammaproteobacteria;Pseudomonadales;Moraxellaceae;Acinetobacter;	Bacteria;Proteobacteria;Gammaproteobacteria;Pseudomonadales;Moraxellaceae;Acinetobacter;
ASV113	0.99423	97.5207	99	Bacteria;"Proteobacteria";Betaproteobacteria;unclassified_Betaproteobacteria;	Bacteria;Proteobacteria;Gammaproteobacteria;Burkholderiales;SC-I-84;
ASV114	0.994394	99.5868	99	Bacteria;"Proteobacteria";Alphaproteobacteria;Sphingomonadales;Sphingomonadaceae;Novosphingobium;	Bacteria;Proteobacteria;Alphaproteobacteria;Sphingomonadales;Sphingomonadaceae;Novosphingobium;
ASV115	0	100	100	Bacteria;"Proteobacteria";Alphaproteobacteria;Rhizobiales;Bradyrhizobiaceae;	Bacteria;Proteobacteria;Alphaproteobacteria;Rhizobiales;Xanthobacteraceae;
ASV116	0	100	100	Bacteria;"Proteobacteria";Alphaproteobacteria;Rhizobiales;Methylobacteriaceae;Methylobacterium;	Bacteria;Proteobacteria;Alphaproteobacteria;Rhizobiales;Beijerinckiaceae;Methylobacterium-Methylorubrum;
ASV117	0	100	100	Bacteria;"Proteobacteria";Alphaproteobacteria;Rhizobiales;Bradyrhizobiaceae;Bosea;	Bacteria;Proteobacteria;Alphaproteobacteria;Rhizobiales;Beijerinckiaceae;Bosea;
ASV118	0	100	100	Bacteria;"Proteobacteria";Alphaproteobacteria;Caulobacterales;Caulobacteraceae;Brevundimonas;	Bacteria;Proteobacteria;Alphaproteobacteria;Caulobacterales;Caulobacteraceae;Brevundimonas;
ASV119	0.994162	97.1311	99	Bacteria;"Proteobacteria";Alphaproteobacteria;Rhodospirillales;Acetobacteraceae;Roseomonas;	Bacteria;Proteobacteria;Alphaproteobacteria;Acetobacterales;Acetobacteraceae;Roseomonas;
ASV120	0	100	100	Bacteria;"Proteobacteria";Gammaproteobacteria;Xanthomonadales;Xanthomonadaceae;Pseudoxanthomonas;	Bacteria;Proteobacteria;Gammaproteobacteria;Xanthomonadales;Xanthomonadaceae;Pseudoxanthomonas;
ASV121	0.981367	95.4545	98	Bacteria;"Actinobacteria";Actinobacteria;Actinobacteridae;Actinomycetales;	Bacteria;Actinobacteriota;Actinobacteria;
ASV122	0	100	100	Bacteria;"Actinobacteria";Actinobacteria;Actinobacteridae;Actinomycetales;Micrococcineae;Intrasporangiaceae;	Bacteria;Actinobacteriota;Actinobacteria;Micrococcales;Intrasporangiaceae;
ASV123	0.991529	99.1736	99	Bacteria;Cyanobacteria/Chloroplast;Chloroplast;Chloroplast;Bacillariophyta;	Bacteria;Cyanobacteria;Cyanobacteriia;Chloroplast;
ASV124	0.994373	98.3471	99	Bacteria;"Proteobacteria";Alphaproteobacteria;Rhizobiales;	Bacteria;Proteobacteria;Alphaproteobacteria;Rhizobiales;Beijerinckiaceae;
ASV125	0	100	100	Bacteria;"Proteobacteria";Alphaproteobacteria;Rhizobiales;Rhizobiaceae;	Bacteria;Proteobacteria;Alphaproteobacteria;Rhizobiales;Rhizobiaceae;
ASV126	0	100	100	Bacteria;"Proteobacteria";Gammaproteobacteria;Pseudomonadales;Moraxellaceae;Acinetobacter;	Bacteria;Proteobacteria;Gammaproteobacteria;Pseudomonadales;Moraxellaceae;Acinetobacter;
ASV127	0.981891	97.9424	98	Bacteria;"Proteobacteria";Alphaproteobacteria;Rhodobacterales;Rhodobacteraceae;	Bacteria;Proteobacteria;Alphaproteobacteria;Rhodobacterales;Rhodobacteraceae;
ASV128	0.994293	96.6942	99	Bacteria;"Proteobacteria";Alphaproteobacteria;Rhodobacterales;Rhodobacteraceae;Rubellimicrobium;	Bacteria;Proteobacteria;Alphaproteobacteria;Rhodobacterales;Rhodobacteraceae;Rubellimicrobium;
ASV129	0.994223	98.3471	99	Bacteria;"Proteobacteria";Alphaproteobacteria;Rhizobiales;Rhizobiales_incertae_sedis;Alsobacter;	Bacteria;Proteobacteria;Alphaproteobacteria;Rhizobiales;
ASV130	0	100	100	Bacteria;"Proteobacteria";Gammaproteobacteria;Pseudomonadales;Moraxellaceae;Acinetobacter;	Bacteria;Proteobacteria;Gammaproteobacteria;Pseudomonadales;Moraxellaceae;Acinetobacter;
ASV131	0.987738	95.0413	98	Bacteria;"Proteobacteria";Betaproteobacteria;	Bacteria;Proteobacteria;Gammaproteobacteria;Burkholderiales;Sutterellaceae;uncultured;

ASV132	0.994217	95.4545	99	Bacteria;"Bacteroidetes";Sphingobacteriia;"Sphingobacteriales";Chitinophagaceae;unclassified_Chitinophagaceae;	Bacteria;Bacteroidota;Bacteroidia;Chitinophagales;Chitinophagaceae;Dinghuibacter;
ASV133	0.994341	98.3471	99	Bacteria;"Proteobacteria";Alphaproteobacteria;Sphingomonadales;Erythrobacteraceae;	Bacteria;Proteobacteria;Alphaproteobacteria;Sphingomonadales;Sphingomonadaceae;Altererythrobacter;
ASV134	0.994336	97.9339	99	Bacteria;"Proteobacteria";Alphaproteobacteria;Sphingomonadales;Sphingomonadaceae; Sphingorhabdus;	Bacteria;Proteobacteria;Alphaproteobacteria;Sphingomonadales;Sphingomonadaceae;Sphingorhabdus;
ASV135	0.99425	94.2149	99	Bacteria;Cyanobacteria/Chloroplast;Cyanobacteria;Family IV;GpIV;	Bacteria;Cyanobacteria;Cyanobacteriia;SepB-3;
ASV136	0.985699	97.1074	98	Bacteria;Cyanobacteria/Chloroplast;Chloroplast;Chloroplast;Bacillariophyta;	Bacteria;Cyanobacteria;Cyanobacteriia;Chloroplast;
ASV137	0.994343	99.5868	99	Bacteria;"Proteobacteria";Alphaproteobacteria;Rhodobacterales;Rhodobacteraceae;Amaricoccus;	Bacteria;Proteobacteria;Alphaproteobacteria;Rhodobacterales;Rhodobacteraceae;
ASV138	0.994439	99.5868	99	Bacteria;"Proteobacteria";Alphaproteobacteria;Sphingomonadales;Sphingomonadaceae; Novosphingobium;	Bacteria;Proteobacteria;Alphaproteobacteria;Sphingomonadales;Sphingomonadaceae;Novosphingobium;
ASV139	0.992071	99.5868	99	Bacteria;Cyanobacteria/Chloroplast;Chloroplast;Chloroplast;Bacillariophyta;	Bacteria;Cyanobacteria;Cyanobacteriia;Chloroplast;
ASV140	0.994226	98.3539	99	Bacteria;"Proteobacteria";Betaproteobacteria;	Bacteria;Proteobacteria;Gammaproteobacteria;Burkholderiales;SC-I-84;
ASV141	0.99214	98.7603	99	Bacteria;"Proteobacteria";Deltaproteobacteria;Desulfobacterales;Desulfobulbaceae;	Bacteria;Desulfobacterota;Desulfobulbia;Desulfobulbales;Desulfocapsaceae;
ASV142	0	100	100	Bacteria;"Proteobacteria";Alphaproteobacteria;Sphingomonadales;Sphingomonadaceae; Polymorphobacter;	Bacteria;Proteobacteria;Alphaproteobacteria;Sphingomonadales;Sphingomonadaceae;Polymorphobacter;
ASV143	0	100	100	Bacteria;"Proteobacteria";Alphaproteobacteria;Sphingomonadales;Sphingomonadaceae; Novosphingobium;	Bacteria;Proteobacteria;Alphaproteobacteria;Sphingomonadales;Sphingomonadaceae;Novosphingobium;
ASV144	0	100	100	Bacteria;"Proteobacteria";Alphaproteobacteria;Sphingomonadales;Sphingomonadaceae; Novosphingobium;	Bacteria;Proteobacteria;Alphaproteobacteria;Sphingomonadales;Sphingomonadaceae;Novosphingobium;
ASV145	0	100	100	Bacteria;"Proteobacteria";Alphaproteobacteria;Rhizobiales;Bradyrhizobiaceae;Bosea;	Bacteria;Proteobacteria;Alphaproteobacteria;Rhizobiales;Beijerinckiaceae;Bosea;
ASV146	0.987966	96.6942	98	Bacteria;"Proteobacteria";Betaproteobacteria;	Bacteria;Proteobacteria;Gammaproteobacteria;Burkholderiales;SC-I-84;
ASV147	0.994317	99.1736	99	Bacteria;Cyanobacteria/Chloroplast;Chloroplast;Chloroplast;Bacillariophyta;	Bacteria;Cyanobacteria;Cyanobacteriia;Chloroplast;
ASV148	0.994233	95.8678	99	Bacteria;"Bacteroidetes";Sphingobacteriia;"Sphingobacteriales";Chitinophagaceae;unclassified_Chitinophagaceae;	Bacteria;Bacteroidota;Bacteroidia;Chitinophagales;Chitinophagaceae;Dinghuibacter;
ASV149	0.9388	96.7078	93	Bacteria;"Proteobacteria";Alphaproteobacteria;Rhodobacterales;Rhodobacteraceae;	Bacteria;Proteobacteria;Alphaproteobacteria;Rhodobacterales;Rhodobacteraceae;
ASV150	0.994366	97.9339	99	Bacteria;"Proteobacteria";Alphaproteobacteria;Rhodobacterales;Rhodobacteraceae;	Bacteria;Proteobacteria;Alphaproteobacteria;Rhodobacterales;Rhodobacteraceae;
ASV151	0	100	100	Bacteria;"Proteobacteria";Alphaproteobacteria;Rhizobiales;Methylobacteriaceae;Methylobacterium;	Bacteria;Proteobacteria;Alphaproteobacteria;Rhizobiales;Beijerinckiaceae;Methylobacterium-Methylorubrum;
ASV152	0.976728	96.281	97	Bacteria;"Proteobacteria";Betaproteobacteria;unclassified_Betaproteobacteria;	Bacteria;Proteobacteria;Gammaproteobacteria;Burkholderiales;Sutterellaceae;AAP99;
ASV153	0	100	100	Bacteria;"Planctomycetes";Planctomycetia;Planctomycetales;Planctomycetaceae;unclassified_Planctomycetaceae;	Bacteria;Planctomycetota;Planctomycetes;Pirellulales;Pirellulaceae;Pir4 lineage;

ASV154	0.977705	92.9752	97	Bacteria;Cyanobacteria/Chloroplast;Chloroplast;Chloroplast;Bacillariophyta;	Bacteria;Cyanobacteria;Cyanobacteriia;Chloroplast;
ASV155	0	100	100	Bacteria;"Proteobacteria";Alphaproteobacteria;Rhizobiales;Xanthobacteraceae;Pseudolabrys;	Bacteria;Proteobacteria;Alphaproteobacteria;Rhizobiales;Xanthobacteraceae;
ASV156	0	100	100	Bacteria;"Proteobacteria";Alphaproteobacteria;Rhizobiales;Rhizobiaceae;Rhizobium;	Bacteria;Proteobacteria;Alphaproteobacteria;Rhizobiales;Rhizobiaceae;Allorhizobium-Neorhizobium-Pararhizobium-Rhizobium;
ASV157	0	100	100	Bacteria;Firmicutes;Bacilli;Lactobacillales;Carnobacteriaceae;Trichococcus;	Bacteria;Firmicutes;Bacilli;Lactobacillales;Carnobacteriaceae;Trichococcus;
ASV158	0	100	100	Bacteria;"Proteobacteria";Gammaproteobacteria;Xanthomonadales;Xanthomonadaceae;Stenotrophomonas;	Bacteria;Proteobacteria;Gammaproteobacteria;Xanthomonadales;Xanthomonadaceae;Stenotrophomonas;
ASV159	0	100	100	Bacteria;"Proteobacteria";Betaproteobacteria;Burkholderiales;Comamonadaceae;Delftia;	Bacteria;Proteobacteria;Gammaproteobacteria;Burkholderiales;Comamonadaceae;Delftia;
ASV160	0.994383	99.1736	99	Bacteria;"Proteobacteria";Alphaproteobacteria;Sphingomonadales;Sphingomonadaceae;Sphingomonas;	Bacteria;Proteobacteria;Alphaproteobacteria;Sphingomonadales;Sphingomonadaceae;Sphingomonas;
ASV161	0	100	100	Bacteria;"Proteobacteria";Alphaproteobacteria;Caulobacterales;Caulobacteraceae;Phenylobacterium;	Bacteria;Proteobacteria;Alphaproteobacteria;Caulobacterales;Caulobacteraceae;Phenylobacterium;
ASV162	0.991679	96.2963	99	Bacteria;"Proteobacteria";Alphaproteobacteria;Rhizobiales;	Bacteria;Proteobacteria;Alphaproteobacteria;Rhizobiales;Rhizobiales Incertae Sedis;Phreatobacter;
ASV164	0.99442	98.7603	99	Bacteria;"Bacteroidetes";Flavobacteriia;"Flavobacteriales";Flavobacteriaceae;Chryseobacterium;	Bacteria;Bacteroidota;Bacteroidia;Flavobacteriales;Weeksellaceae;Chryseobacterium;
ASV165	0	100	100	Bacteria;"Proteobacteria";Alphaproteobacteria;Rhizobiales;Bradyrhizobiaceae;	Bacteria;Proteobacteria;Alphaproteobacteria;Rhizobiales;Xanthobacteraceae;
ASV166	0.994355	98.7603	99	Bacteria;"Proteobacteria";Alphaproteobacteria;Sphingomonadales;	Bacteria;Proteobacteria;Alphaproteobacteria;Sphingomonadales;Sphingomonadaceae;
ASV167	0	100	100	Bacteria;"Proteobacteria";Alphaproteobacteria;Rhodobacterales;Rhodobacteraceae;Gemmobacter;	Bacteria;Proteobacteria;Alphaproteobacteria;Rhodobacterales;Rhodobacteraceae;
ASV168	0	100	100	Bacteria;"Proteobacteria";Alphaproteobacteria;Rhizobiales;Methylobacteriaceae;Microvirga;	Bacteria;Proteobacteria;Alphaproteobacteria;Rhizobiales;Beijerinckiaceae;Microvirga;
ASV169	0	100	100	Bacteria;"Proteobacteria";Alphaproteobacteria;Rhizobiales;Hyphomicrobiaceae;Devosia;	Bacteria;Proteobacteria;Alphaproteobacteria;Rhizobiales;Devosiaceae;Devosia;
ASV170	0.991809	97.1193	99	Bacteria;"Actinobacteria";Actinobacteria;Rubrobacteridae;Solirubrobacterales;	Bacteria;Actinobacteriota;Thermoleophilia;Solirubrobacterales;Solirubrobacteraceae;Conexibacter;
ASV171	0.994231	99.5868	99	Bacteria;"Proteobacteria";Gammaproteobacteria;Xanthomonadales;Sinobacteraceae;Povalibacter;	Bacteria;Proteobacteria;Gammaproteobacteria;Steroidobacterales;Steroidobacteraceae;uncultured;
ASV172	0.982317	98.3471	98	Bacteria;"Proteobacteria";Alphaproteobacteria;Rhodobacterales;Rhodobacteraceae;unclassified_Rhodobacteraceae;	Bacteria;Proteobacteria;Alphaproteobacteria;Rhodobacterales;Rhodobacteraceae;
ASV173	0	100	100	Bacteria;Firmicutes;Bacilli;Bacillales;Bacillales_Incertae Sedis XII;Exiguobacterium;	Bacteria;Firmicutes;Bacilli;Exiguobacterales;Exiguobacteraceae;Exiguobacterium;
ASV174	0.987712	99.1736	98	Bacteria;"Proteobacteria";Gammaproteobacteria;Xanthomonadales;Xanthomonadaceae;Silanimonas;	Bacteria;Proteobacteria;Gammaproteobacteria;Xanthomonadales;Xanthomonadaceae;Silanimonas;
ASV175	0.994079	97.5207	99	Bacteria;"Planctomycetes";Planctomycetia;Planctomycetales;Planctomycetaceae;	Bacteria;Planctomycetota;Planctomycetes;Gemmatales;Gemmataceae;Fimbriiglobus;
ASV176	0.994122	98.3539	99	Bacteria;"Verrucomicrobia";Verrucomicrobiae;Verrucomicrobiales;Verrucomicrobiaceae;	Bacteria;Verrucomicrobiota;Verrucomicrobiae;Verrucomicrobiales;Rubritaleaceae;Luteolibacter;
ASV177	0	100	100	Bacteria;"Proteobacteria";Alphaproteobacteria;Rhizobiales;"Aurantimonadaceae";Aureimonas;	Bacteria;Proteobacteria;Alphaproteobacteria;Rhizobiales;Rhizobiaceae;Aureimonas;

ASV178	0	100	100	Bacteria;"Proteobacteria";Alphaproteobacteria;Rhizobiales;Methylocystaceae;Methylocystis;	Bacteria;Proteobacteria;Alphaproteobacteria;Rhizobiales;Beijerinckiaceae;Methylocystis;
ASV179	0.993926	98.3539	99	Bacteria;"Chloroflexi";unclassified_"Chloroflexi";	Bacteria;Chloroflexi;KD4-96;
ASV180	0.994361	99.5868	99	Bacteria;"Proteobacteria";Alphaproteobacteria;Sphingomonadales;Sphingomonadaceae;Sphingomonas;	Bacteria;Proteobacteria;Alphaproteobacteria;Sphingomonadales;Sphingomonadaceae;Sphingomonas;
ASV181	0.978406	90.535	97	Bacteria;"Gemmatimonadetes";Gemmatimonadetes;Gemmatimonadales;Gemmatimonadaceae;Gemmatimonas;	Bacteria;Gemmatimonadota;Gemmatimonadetes;Gemmatimonadales;Gemmatimonadaceae;Gemmatimonas;
ASV182	0	100	100	Bacteria;"Proteobacteria";Alphaproteobacteria;Sphingomonadales;Sphingomonadaceae;Sphingomonas;	Bacteria;Proteobacteria;Alphaproteobacteria;Sphingomonadales;Sphingomonadaceae;Sphingomonas;
ASV183	0.994233	98.3539	99	Bacteria;"Bacteroidetes";Sphingobacteriia;"Sphingobacteriales";Chitinophagaceae;Flavisolibacter;	Bacteria;Bacteroidota;Bacteroidia;Chitinophagales;Chitinophagaceae;Flavisolibacter;
ASV184	0.990843	92.9752	99	Bacteria;Cyanobacteria/Chloroplast;Cyanobacteria;Family IV;GpIV;	Bacteria;Cyanobacteria;Cyanobacteriia;SepB-3;
ASV185	0	100	100	Bacteria;"Proteobacteria";Alphaproteobacteria;Rhodobacterales;Rhodobacteraceae;Paracoccus;	Bacteria;Proteobacteria;Alphaproteobacteria;Rhodobacterales;Rhodobacteraceae;Paracoccus;
ASV186	0.994255	99.5868	99	Bacteria;"Proteobacteria";Alphaproteobacteria;Sphingomonadales;Sphingomonadaceae;	Bacteria;Proteobacteria;Alphaproteobacteria;Sphingomonadales;Sphingomonadaceae;Sandaracinobacter;
ASV187	0.994427	99.5868	99	Bacteria;"Proteobacteria";Betaproteobacteria;Burkholderiales;Alcaligenaceae;Azohydromonas;	Bacteria;Proteobacteria;Gammaproteobacteria;Burkholderiales;Comamonadaceae;Azohydromonas;
ASV188	0	100	100	Bacteria;"Actinobacteria";Actinobacteria;Actinobacteridae;Actinomycetales;Propionibacterineae;Nocardioidaceae;Nocardioides;	Bacteria;Actinobacteriota;Actinobacteria;Propionibacteriales;Nocardioidaceae;Nocardioides;
ASV189	0	100	100	Bacteria;"Proteobacteria";Alphaproteobacteria;Sphingomonadales;Sphingomonadaceae;	Bacteria;Proteobacteria;Alphaproteobacteria;Sphingomonadales;Sphingomonadaceae;
ASV190	0.991691	97.1074	99	Bacteria;"Proteobacteria";Betaproteobacteria;unclassified_Betaproteobacteria;	Bacteria;Proteobacteria;Gammaproteobacteria;Burkholderiales;SC-I-84;
ASV191	0	100	100	Bacteria;"Proteobacteria";Alphaproteobacteria;Rhodobacterales;Rhodobacteraceae;	Bacteria;Proteobacteria;Alphaproteobacteria;Rhodobacterales;Rhodobacteraceae;
ASV192	0	100	100	Bacteria;"Proteobacteria";Betaproteobacteria;Burkholderiales;Oxalobacteraceae;Massilia;	Bacteria;Proteobacteria;Gammaproteobacteria;Burkholderiales;Oxalobacteraceae;Massilia;
ASV193	0.994397	99.5868	99	Bacteria;"Proteobacteria";Betaproteobacteria;Rhodocyclales;Rhodocyclaceae;Dechloromonas;	Bacteria;Proteobacteria;Gammaproteobacteria;Burkholderiales;Rhodocyclaceae;
ASV194	0.994332	97.9339	99	Bacteria;"Bacteroidetes";Sphingobacteriia;"Sphingobacteriales";Chitinophagaceae;Flavihumibacter;	Bacteria;Bacteroidota;Bacteroidia;Chitinophagales;Chitinophagaceae;Flavihumibacter;
ASV196	0	100	100	Bacteria;"Proteobacteria";Alphaproteobacteria;Sphingomonadales;Sphingomonadaceae;Sphingomonas;	Bacteria;Proteobacteria;Alphaproteobacteria;Sphingomonadales;Sphingomonadaceae;Sphingomonas;
ASV197	0.97496	88.0165	97	Unclassified;	Bacteria; Thermi; Deinococci; Deinococcales; Trueperaceae;
ASV198	0.985964	91.3223	98	Bacteria;"Planctomycetes";Planctomycetia;Planctomycetales;Planctomycetaceae;unclassified_Planctomycetaceae;	Bacteria;Planctomycetota;Planctomycetes;Gemmatales;Gemmataceae;uncultured;
ASV199	0.994384	99.5868	99	Bacteria;"Proteobacteria";Alphaproteobacteria;Sphingomonadales;Sphingomonadaceae;Sphingomonas;	Bacteria;Proteobacteria;Alphaproteobacteria;Sphingomonadales;Sphingomonadaceae;Sphingomonas;

ASV200	0.956356	94.6281	95	Bacteria;"Proteobacteria";Alphaproteobacteria;Rhizobiales;unclassified_Rhizobiales;	Bacteria;Proteobacteria;Alphaproteobacteria;Rhizobiales;Rhizobiales Incertae Sedis;Phreatobacter;
ASV201	0.989317	99.1736	98	Bacteria;"Proteobacteria";Alphaproteobacteria;	Bacteria;Proteobacteria;Alphaproteobacteria;Rhizobiales;Rhizobiales Incertae Sedis;uncultured;
ASV202	0	100	100	Bacteria;"Proteobacteria";Alphaproteobacteria;Sphingomonadales;Sphingomonadaceae; Sphingomonas;	Bacteria;Proteobacteria;Alphaproteobacteria;Sphingomonadales;Sphingomonadaceae;Sphingomonas;
ASV203	0.991428	96.281	99	Bacteria;"Planctomycetes";Planctomycetia;Planctomycetales;Planctomycetaceae;	Bacteria;Planctomycetota;Planctomycetes;Gemmatales;Gemmataceae;Fimbriiglobus;
ASV204	0	100	100	Bacteria;"Proteobacteria";Gammaproteobacteria;Pseudomonadales;Moraxellaceae;Acinetobacter;	Bacteria;Proteobacteria;Gammaproteobacteria;Pseudomonadales;Moraxellaceae;Acinetobacter;
ASV205	0	100	100	Bacteria;"Proteobacteria";Gammaproteobacteria;"Enterobacteriales";Enterobacteriaceae; Pantoea;	Bacteria;Proteobacteria;Gammaproteobacteria;Enterobacterales;Erwiniaceae;
ASV206	0.988113	97.1074	98	Bacteria;"Proteobacteria";Betaproteobacteria;	Bacteria;Proteobacteria;Gammaproteobacteria;Burkholderiales;SC-I-84;
ASV207	0.994419	99.1736	99	Bacteria;"Proteobacteria";Alphaproteobacteria;Caulobacterales;Caulobacteraceae;Brevundimonas;	Bacteria;Proteobacteria;Alphaproteobacteria;Caulobacterales;Caulobacteraceae; Brevundimonas;
ASV208	0.994199	97.1193	99	Bacteria;"Proteobacteria";Betaproteobacteria;unclassified_Betaproteobacteria;	Bacteria;Proteobacteria;Gammaproteobacteria;Burkholderiales;SC-I-84;
ASV209	0.984075	96.281	98	Bacteria;"Actinobacteria";	Bacteria;Actinobacteriota;Thermoleophilia;Solirubrobacterales;67-14;
ASV210	0.994269	97.5207	99	Bacteria;"Proteobacteria";Alphaproteobacteria;Rhizobiales;	Bacteria;Proteobacteria;Alphaproteobacteria;Rhizobiales;Rhizobiales Incertae Sedis;Nordella;
ASV211	0	100	100	Bacteria;"Proteobacteria";Betaproteobacteria;Burkholderiales;Comamonadaceae;Polaromonas;	Bacteria;Proteobacteria;Gammaproteobacteria;Burkholderiales;Comamonadaceae;Polaromonas;
ASV212	0.97552	97.1074	97	Bacteria;"Proteobacteria";Alphaproteobacteria;Rhizobiales;Hyphomicrobiaceae;Hyphomicrobium;	Bacteria;Proteobacteria;Alphaproteobacteria;Rhizobiales;Hyphomicrobiaceae;
ASV213	0.944197	94.6281	94	Bacteria;Firmicutes;Bacilli;Bacillales;	Bacteria;Firmicutes;Bacilli;Bacillales;Bacillaceae;Bacillus;
ASV214	0.994268	96.281	99	Bacteria;"Actinobacteria";Actinobacteria;Actinobacteridae;Actinomycetales;	Bacteria;Actinobacteriota;Actinobacteria;
ASV215	0.994369	98.7603	99	Bacteria;"Proteobacteria";Alphaproteobacteria;	Bacteria;Proteobacteria;Alphaproteobacteria;Rhizobiales;
ASV216	0	100	100	Bacteria;"Proteobacteria";Gammaproteobacteria;Pseudomonadales;Moraxellaceae;Acinetobacter;	Bacteria;Proteobacteria;Gammaproteobacteria;Pseudomonadales;Moraxellaceae;Acinetobacter;
ASV217	0.994349	99.5868	99	Bacteria;"Proteobacteria";Alphaproteobacteria;Sphingomonadales;Sphingomonadaceae;	Bacteria;Proteobacteria;Alphaproteobacteria;Sphingomonadales;Sphingomonadaceae;Sphingomonas;
ASV218	0	100	100	Bacteria;Cyanobacteria/Chloroplast;Cyanobacteria;	Bacteria;Cyanobacteria;Cyanobacteriia;Cyanobacteriales;Phormidiaceae;Tychonema CCAP 1459-11B;
ASV219	0.985725	92.6531	98	Bacteria;"Actinobacteria";Actinobacteria;Acidimicrobidae;Acidimicrobiales;"Acidimicrobineae";	Bacteria;Actinobacteriota;Acidimicrobiia;Microtrichales;uncultured;
ASV220	0.947229	91.7355	94	Bacteria;"Proteobacteria";Alphaproteobacteria;Rhizobiales;	Bacteria;Proteobacteria;Alphaproteobacteria;Rhizobiales;Beijerinckiaceae;Fuku N57;

ASV221	0	100	100	Bacteria;"Proteobacteria";Alphaproteobacteria;Rhodobacterales;Rhodobacteraceae;	Bacteria;Proteobacteria;Alphaproteobacteria;Rhodobacterales;Rhodobacteraceae;Paracoccus;
ASV222	0	100	100	Bacteria;"Actinobacteria";Actinobacteria;Actinobacteridae;Actinomycetales;Corynebacterineae;Mycobacteriaceae;Mycobacterium;	Bacteria;Actinobacteriota;Actinobacteria;Corynebacteriales;Mycobacteriaceae;Mycobacterium;
ASV223	0	100	100	Bacteria;"Proteobacteria";Alphaproteobacteria;Caulobacterales;Caulobacteraceae;Brevundimonas;	Bacteria;Proteobacteria;Alphaproteobacteria;Caulobacterales;Caulobacteraceae;Brevundimonas;
ASV224	0	100	100	Bacteria;"Proteobacteria";Betaproteobacteria;Burkholderiales;Comamonadaceae;Comamonas;	Bacteria;Proteobacteria;Gammaproteobacteria;Burkholderiales;Comamonadaceae;Comamonas;
ASV225	0.994148	99.5868	99	Bacteria;"Proteobacteria";Alphaproteobacteria;Sphingomonadales;Sphingomonadaceae;Polymorphobacter;	Bacteria;Proteobacteria;Alphaproteobacteria;Sphingomonadales;Sphingomonadaceae;Polymorphobacter;
ASV226	0.994272	99.1736	99	Bacteria;"Proteobacteria";Alphaproteobacteria;Rhizobiales;Beijerinckiaceae;Camelimonas;	Bacteria;Proteobacteria;Alphaproteobacteria;Rhizobiales;Beijerinckiaceae;Camelimonas;
ASV227	0.938817	96.7078	93	Bacteria;"Proteobacteria";Alphaproteobacteria;Rhodobacterales;Rhodobacteraceae;	Bacteria;Proteobacteria;Alphaproteobacteria;Rhodobacterales;Rhodobacteraceae;
ASV228	0.991155	98.3471	99	Bacteria;"Proteobacteria";Alphaproteobacteria;Rhizobiales;unclassified_Rhizobiales;	Bacteria;Proteobacteria;Alphaproteobacteria;Rhizobiales;Rhizobiales Incertae Sedis;uncultured;
ASV230	0	100	100	Bacteria;"Proteobacteria";Alphaproteobacteria;Rhodobacterales;Rhodobacteraceae;Paracoccus;	Bacteria;Proteobacteria;Alphaproteobacteria;Rhodobacterales;Rhodobacteraceae;Paracoccus;
ASV231	0	100	100	Bacteria;"Actinobacteria";Actinobacteria;Actinobacteridae;Actinomycetales;Micrococcineae;Microbacteriaceae;Curtobacterium;	Bacteria;Actinobacteriota;Actinobacteria;Micrococcales;Microbacteriaceae;Curtobacterium;
ASV232	0	100	100	Bacteria;"Proteobacteria";Betaproteobacteria;Burkholderiales;Alcaligenaceae;Achromobacter;	Bacteria;Proteobacteria;Gammaproteobacteria;Burkholderiales;Alcaligenaceae;
ASV233	0.987484	98.7603	98	Bacteria;"Proteobacteria";Gammaproteobacteria;Xanthomonadales;Xanthomonadaceae;Silanimonas;	Bacteria;Proteobacteria;Gammaproteobacteria;Xanthomonadales;Xanthomonadaceae;Silanimonas;
ASV234	0.994349	98.3471	99	Bacteria;"Proteobacteria";Gammaproteobacteria;Xanthomonadales;Xanthomonadaceae;Lysobacter;	Bacteria;Proteobacteria;Gammaproteobacteria;Xanthomonadales;Xanthomonadaceae;Lysobacter;
ASV235	0	100	100	Bacteria;Firmicutes;Bacilli;Bacillales;Bacillaceae 1;Bacillus;	Bacteria;Firmicutes;Bacilli;Bacillales;Bacillaceae;Bacillus;
ASV236	0.994423	99.5868	99	Bacteria;"Proteobacteria";Alphaproteobacteria;Sphingomonadales;Sphingomonadaceae;Novosphingobium;	Bacteria;Proteobacteria;Alphaproteobacteria;Sphingomonadales;Sphingomonadaceae;Novosphingobium;
ASV237	0.99444	99.5868	99	Bacteria;"Proteobacteria";Betaproteobacteria;Burkholderiales;Comamonadaceae;	Bacteria;Proteobacteria;Gammaproteobacteria;Burkholderiales;Comamonadaceae;
ASV238	0.994257	96.2963	99	Bacteria;"Proteobacteria";Alphaproteobacteria;Rhizobiales;	Bacteria;Proteobacteria;Alphaproteobacteria;Rhizobiales;
ASV239	0.994337	98.3471	99	Bacteria;"Proteobacteria";Alphaproteobacteria;Sphingomonadales;Sphingomonadaceae;Sphingorhabdus;	Bacteria;Proteobacteria;Alphaproteobacteria;Sphingomonadales;Sphingomonadaceae;Sphingorhabdus;
ASV240	0	100	100	Bacteria;"Actinobacteria";Actinobacteria;Actinobacteridae;Actinomycetales;Micrococcineae;Promicromonosporaceae;Cellulosimicrobium;	Bacteria;Actinobacteriota;Actinobacteria;Micrococcales;Promicromonosporaceae;Cellulosimicrobium;
ASV241	0.938774	96.7078	93	Bacteria;"Proteobacteria";Alphaproteobacteria;Rhodobacterales;Rhodobacteraceae;	Bacteria;Proteobacteria;Alphaproteobacteria;Rhodobacterales;Rhodobacteraceae;
ASV242	0	100	100	Bacteria;"Proteobacteria";Betaproteobacteria;Burkholderiales;Comamonadaceae;Pseudorhodoferax;	Bacteria;Proteobacteria;Gammaproteobacteria;Burkholderiales;Comamonadaceae;Pseudorhodoferax;
ASV243	0	100	100	Bacteria;"Proteobacteria";Alphaproteobacteria;Rhizobiales;Methylobacteriaceae;Microvirga;	Bacteria;Proteobacteria;Alphaproteobacteria;Rhizobiales;Beijerinckiaceae;Microvirga;

ASV244	0	100	100	Bacteria;"Proteobacteria";Alphaproteobacteria;Rhizobiales;Rhizobiaceae;Rhizobium;	Bacteria;Proteobacteria;Alphaproteobacteria;Rhizobiales;Rhizobiaceae;Allorhizobium-Neorhizobium-Pararhizobium-Rhizobium;
ASV245	0	100	100	Bacteria;"Actinobacteria";Actinobacteria;Actinobacteridae;Actinomycetales;Propionibacterineae;Nocardioidaceae;Aeromicrobium;	Bacteria;Actinobacteriota;Actinobacteria;Propionibacteriales;Nocardioidaceae;Aeromicrobium;
ASV246	0.994	98.3607	99	Bacteria;"Verrucomicrobia";Verrucomicrobiae;Verrucomicrobiales;Verrucomicrobiaceae;unclassified_Verrucomicrobiaceae;	Bacteria;Verrucomicrobiota;Verrucomicrobiae;Verrucomicrobiales;Verrucomicrobiaceae;uncultured;
ASV247	0.994384	99.1736	99	Bacteria;"Proteobacteria";Alphaproteobacteria;Sphingomonadales;Sphingomonadaceae;Sphingomonas;	Bacteria;Proteobacteria;Alphaproteobacteria;Sphingomonadales;Sphingomonadaceae;Sphingomonas;
ASV248	0.988223	98.3471	98	Bacteria;Cyanobacteria/Chloroplast;Chloroplast;Chloroplast;Bacillariophyta;	Bacteria;Cyanobacteria;Cyanobacteriia;Chloroplast;
ASV249	0	100	100	Bacteria;Cyanobacteria/Chloroplast;Chloroplast;Chloroplast;Streptophyta;	Bacteria;Cyanobacteria;Cyanobacteriia;Chloroplast;
ASV250	0	100	100	Bacteria;"Proteobacteria";Gammaproteobacteria;Xanthomonadales;Sinobacteraceae;	Bacteria;Proteobacteria;Gammaproteobacteria;Steroidobacterales;Steroidobacteraceae;uncultured;
ASV251	0.994281	96.6942	99	Bacteria;"Proteobacteria";Betaproteobacteria;Burkholderiales;Oxalobacteraceae;	Bacteria;Proteobacteria;Gammaproteobacteria;Burkholderiales;
ASV252	0.994389	99.1736	99	Bacteria;"Proteobacteria";Alphaproteobacteria;Sphingomonadales;Erythrobacteraceae;Altererythrobacter;	Bacteria;Proteobacteria;Alphaproteobacteria;Sphingomonadales;Sphingomonadaceae;
ASV253	0.97986	96.6942	97	Bacteria;"Proteobacteria";Betaproteobacteria;unclassified_Betaproteobacteria;	Bacteria;Proteobacteria;Gammaproteobacteria;Burkholderiales;SC-I-84;
ASV254	0.983752	98.3471	98	Bacteria;"Proteobacteria";Alphaproteobacteria;Rhodobacterales;Rhodobacteraceae;	Bacteria;Proteobacteria;Alphaproteobacteria;Rhodobacterales;Rhodobacteraceae;
ASV255	0.985064	95.8678	98	Bacteria;	Bacteria;Chloroflexi;KD4-96;
ASV256	0	100	100	Bacteria;"Actinobacteria";Actinobacteria;Actinobacteridae;Actinomycetales;Propionibacterineae;Nocardioidaceae;Nocardioides;	Bacteria;Actinobacteriota;Actinobacteria;Propionibacteriales;Nocardioidaceae;Nocardioides;
ASV257	0	100	100	Bacteria;"Proteobacteria";Betaproteobacteria;Burkholderiales;Comamonadaceae;Hydrogenophaga;	Bacteria;Proteobacteria;Gammaproteobacteria;Burkholderiales;Comamonadaceae;Hydrogenophaga;
ASV258	0.963499	97.9339	96	Bacteria;"Proteobacteria";Alphaproteobacteria;Rhizobiales;	Bacteria;Proteobacteria;Alphaproteobacteria;Rhizobiales;
ASV259	0.967794	96.281	96	Bacteria;"Proteobacteria";Alphaproteobacteria;Rhizobiales;unclassified_Rhizobiales;	Bacteria;Proteobacteria;Alphaproteobacteria;Rhizobiales;Rhizobiales Incertae Sedis;uncultured;
ASV260	0.994422	100	99	Bacteria;Firmicutes;Bacilli;Bacillales;Bacillaceae 1;Bacillus;	Bacteria;Firmicutes;Bacilli;Bacillales;Bacillaceae;Bacillus;
ASV261	0	100	100	Bacteria;"Actinobacteria";Actinobacteria;Actinobacteridae;Actinomycetales;Propionibacterineae;Nocardioidaceae;Nocardioides;	Bacteria;Actinobacteriota;Actinobacteria;Propionibacteriales;Nocardioidaceae;Nocardioides;
ASV262	0.994311	99.1736	99	Bacteria;"Bacteroidetes";Sphingobacteriia;"Sphingobacteriales";Chitinophagaceae;Terrimonas;	Bacteria;Bacteroidota;Bacteroidia;Chitinophagales;Chitinophagaceae;Terrimonas;
ASV263	0.981214	97.9508	98	Bacteria;"Actinobacteria";Actinobacteria;Acidimicrobidae;Acidimicrobiales;"Acidimicrobineae";Acidimicrobiaceae;Ilumatobacter;	Bacteria;Actinobacteriota;Acidimicrobiia;Microtrichales;Ilumatobacteraceae;Ilumatobacter;
ASV264	0.987813	97.1193	98	Bacteria;"Proteobacteria";Alphaproteobacteria;Rhodobacterales;Rhodobacteraceae;Rubellimicrobium;	Bacteria;Proteobacteria;Alphaproteobacteria;Rhodobacterales;Rhodobacteraceae;Rubellimicrobium;
ASV265	0.994018	93.9024	99	Bacteria;"Proteobacteria";Gammaproteobacteria;	Bacteria;Proteobacteria;Gammaproteobacteria;PLTA13;

ASV266	0.991434	96.7213	99	Bacteria;"Verrucomicrobia";Verrucomicrobiae;Verrucomicrobiales;Verrucomicrobiaceae;unclassified_Verrucomicrobiaceae;	Bacteria;Verrucomicrobiota;Verrucomicrobiae;Verrucomicrobiales;Verrucomicrobiaceae;uncultured;
ASV267	0	100	100	Bacteria;"Planctomycetes";Planctomycetia;Planctomycetales;Planctomycetaceae;unclassified_Planctomycetaceae;	Bacteria;Planctomycetota;Planctomycetes;Pirellulales;Pirellulaceae;uncultured;
ASV268	0	100	100	Bacteria;"Bacteroidetes";Sphingobacteriia;"Sphingobacteriales";Chitinophagaceae;Cnuella;	Bacteria;Bacteroidota;Bacteroidia;Chitinophagales;Chitinophagaceae;Cnuella;
ASV269	0	100	100	Bacteria;"Proteobacteria";Betaproteobacteria;Burkholderiales;Oxalobacteraceae;Massilia;	Bacteria;Proteobacteria;Gammaproteobacteria;Burkholderiales;Oxalobacteraceae;Massilia;
ASV270	0.958963	86.4198	95	Bacteria;"Planctomycetes";Planctomycetia;Planctomycetales;Planctomycetaceae;Pirellula;	Bacteria;Planctomycetota;Planctomycetes;Pirellulales;Pirellulaceae;uncultured;
ASV271	0.972673	96.6942	97	Bacteria;"Proteobacteria";Alphaproteobacteria;Rhizobiales;Hyphomicrobiaceae;	Bacteria;Proteobacteria;Alphaproteobacteria;Rhizobiales;Hyphomicrobiaceae;Pedomicrobium;
ASV272	0.991765	97.1074	99	Bacteria;"Proteobacteria";Alphaproteobacteria;Sphingomonadales;Sphingomonadaceae;	Bacteria;Proteobacteria;Alphaproteobacteria;Sphingomonadales;Sphingomonadaceae;Sandaracinobacter;
ASV273	0.994436	97.9424	99	Bacteria;"Proteobacteria";Alphaproteobacteria;Rhizobiales;Xanthobacteraceae;	Bacteria;Proteobacteria;Alphaproteobacteria;Rhizobiales;Xanthobacteraceae;
ASV274	0	100	100	Bacteria;"Proteobacteria";Alphaproteobacteria;Rhizobiales;Rhizobiaceae;	Bacteria;Proteobacteria;Alphaproteobacteria;Rhizobiales;Rhizobiaceae;
ASV275	0.987847	96.7078	98	Bacteria;"Actinobacteria";Actinobacteria;Rubrobacteridae;Gaiellales;Gaiellaceae;Gaiella;	Bacteria;Actinobacteriota;Thermoleophilia;Gaiellales;Gaiellaceae;Gaiella;
ASV276	0.988109	99.5868	98	Bacteria;"Actinobacteria";Actinobacteria;Actinobacteridae;Actinomycetales;Propionibacterineae;Nocardioidaceae;	Bacteria;Actinobacteriota;Actinobacteria;Propionibacteriales;Nocardioidaceae;
ASV277	0.994329	99.5868	99	Bacteria;"Proteobacteria";Alphaproteobacteria;Sphingomonadales;Sphingomonadaceae;Sphingomonas;	Bacteria;Proteobacteria;Alphaproteobacteria;Sphingomonadales;Sphingomonadaceae;Sphingomonas;
ASV278	0.981074	95.4918	98	Bacteria;"Actinobacteria";Actinobacteria;Acidimicrobidae;Acidimicrobiales;"Acidimicrobineae";Acidimicrobiaceae;Ilumatobacter;	Bacteria;Actinobacteriota;Acidimicrobiia;Microtrichales;Ilumatobacteraceae;CL500-29 marine group;
ASV279	0	100	100	Bacteria;"Actinobacteria";Actinobacteria;Actinobacteridae;Actinomycetales;Micrococcineae;Intrasporangiaceae;	Bacteria;Actinobacteriota;Actinobacteria;Micrococcales;Intrasporangiaceae;
ASV280	0	100	100	Bacteria;"Actinobacteria";Actinobacteria;Acidimicrobidae;Acidimicrobiales;"Acidimicrobineae";Iamiaceae;Aquihabitans;	Bacteria;Actinobacteriota;Acidimicrobiia;Microtrichales;Iamiaceae;Iamia;
ASV281	0.981819	98.7603	98	Bacteria;"Proteobacteria";Alphaproteobacteria;Sphingomonadales;Sphingomonadaceae;Sphingopyxis;	Bacteria;Proteobacteria;Alphaproteobacteria;Sphingomonadales;Sphingomonadaceae;Sphingopyxis;
ASV282	0.994096	97.9424	99	Bacteria;"Verrucomicrobia";Verrucomicrobiae;Verrucomicrobiales;Verrucomicrobiaceae;	Bacteria;Verrucomicrobiota;Verrucomicrobiae;Verrucomicrobiales;Rubritaleaceae;Luteolibacter;
ASV283	0	100	100	Bacteria;"Proteobacteria";Alphaproteobacteria;Rhizobiales;Xanthobacteraceae;	Bacteria;Proteobacteria;Alphaproteobacteria;Rhizobiales;Xanthobacteraceae;
ASV284	0.994304	99.1736	99	Bacteria;"Acidobacteria";Acidobacteria_Gp6;Gp6;	Bacteria;Acidobacteriota;Vicinamibacteria;Vicinamibacterales;Vicinamibacteraceae;Luteitalea;
ASV285	0	100	100	Bacteria;"Proteobacteria";Gammaproteobacteria;Pseudomonadales;Pseudomonadaceae;Pseudomonas;	Bacteria;Proteobacteria;Gammaproteobacteria;Pseudomonadales;Pseudomonadaceae;Pseudomonas;
ASV286	0	100	100	Bacteria;"Bacteroidetes";Flavobacteriia;"Flavobacteriales";Flavobacteriaceae;Cloacibacterium;	Bacteria;Bacteroidota;Bacteroidia;Flavobacteriales;Weeksellaceae;Cloacibacterium;
ASV287	0.986477	93.8776	98	Bacteria;"Verrucomicrobia";Verrucomicrobiae;Verrucomicrobiales;Verrucomicrobiaceae;Luteolibacter;	Bacteria;Verrucomicrobiota;Verrucomicrobiae;Verrucomicrobiales;Rubritaleaceae;Luteolibacter;

ASV288	0.988206	99.1736	98	Bacteria;"Proteobacteria";Betaproteobacteria;Burkholderiales;Oxalobacteraceae;	Bacteria;Proteobacteria;Gammaproteobacteria;Burkholderiales;Oxalobacteraceae;
ASV289	0	100	100	Bacteria;"Actinobacteria";Actinobacteria;Actinobacteridae;Actinomycetales;Corynebacterineae;Mycobacteriaceae;Mycobacterium;	Bacteria;Actinobacteriota;Actinobacteria;Corynebacteriales;Mycobacteriaceae;Mycobacterium;
ASV290	0.994401	99.5868	99	Bacteria;"Proteobacteria";Betaproteobacteria;Burkholderiales;Burkholderiaceae;Cupriavidus;	Bacteria;Proteobacteria;Gammaproteobacteria;Burkholderiales;Burkholderiaceae;Cupriavidus;
ASV291	0	100	100	Bacteria;"Actinobacteria";Actinobacteria;Actinobacteridae;Actinomycetales;Propionibacterineae;Nocardioidaceae;	Bacteria;Actinobacteriota;Actinobacteria;Propionibacteriales;Nocardioidaceae;Nocardioides;
ASV292	0	100	100	Bacteria;"Actinobacteria";Actinobacteria;Actinobacteridae;Actinomycetales;Micrococcineae;Micrococcaceae;Arthrobacter;	Bacteria;Actinobacteriota;Actinobacteria;Micrococcales;Micrococcaceae;Glutamicibacter;
ASV293	0.994351	97.5207	99	Bacteria;"Proteobacteria";Alphaproteobacteria;Rhizobiales;	Bacteria;Proteobacteria;Alphaproteobacteria;Rhizobiales;
ASV294	0.994413	98.3471	99	Bacteria;"Proteobacteria";Alphaproteobacteria;Rhizobiales;Hyphomicrobiaceae;Hyphomicrobium;	Bacteria;Proteobacteria;Alphaproteobacteria;Rhizobiales;Hyphomicrobiaceae;Hyphomicrobium;
ASV295	0.975036	94.2149	97	Bacteria;"Proteobacteria";Gammaproteobacteria;Xanthomonadales;Xanthomonadaceae;	Bacteria;Proteobacteria;Gammaproteobacteria;Xanthomonadales;Rhodanobacteraceae;
ASV296	0	100	100	Bacteria;Nitrospirae;"Nitrospira";"Nitrospirales";"Nitrospiraceae";Nitrospira;	Bacteria;Nitrospirota;Nitrospiria;Nitrospirales;Nitrospiraceae;Nitrospira;
ASV297	0.966885	96.6942	96	Bacteria;"Proteobacteria";Alphaproteobacteria;Rhizobiales;Hyphomicrobiaceae;	Bacteria;Proteobacteria;Alphaproteobacteria;Rhizobiales;Hyphomicrobiaceae;
ASV298	0.980426	95.4733	98	Bacteria;"Actinobacteria";Actinobacteria;Rubrobacteridae;Gaiellales;Gaiellaceae;Gaiella;	Bacteria;Actinobacteriota;Thermoleophilia;Gaiellales;
ASV299	0.994378	98.3471	99	Bacteria;"Proteobacteria";Alphaproteobacteria;Sphingomonadales;Sphingomonadaceae;Novosphingobium;	Bacteria;Proteobacteria;Alphaproteobacteria;Sphingomonadales;Sphingomonadaceae;Novosphingobium;
ASV300	0.994027	93.3884	99	Bacteria;Cyanobacteria/Chloroplast;Cyanobacteria;Family IV;GpIV;	Bacteria;Cyanobacteria;Cyanobacteriia;SepB-3;
ASV301	0	100	100	Bacteria;"Proteobacteria";Alphaproteobacteria;Rhodospirillales;Acetobacteraceae;Roseomonas;	Bacteria;Proteobacteria;Alphaproteobacteria;Acetobacterales;Acetobacteraceae;Roseomonas;
ASV302	0.994371	98.7603	99	Bacteria;"Actinobacteria";Actinobacteria;Actinobacteridae;Actinomycetales;Kineosporiineae;Kineosporiaceae;	Bacteria;Actinobacteriota;Actinobacteria;Kineosporiales;Kineosporiaceae;
ASV303	0.960513	93.4156	96	Bacteria;"Proteobacteria";Alphaproteobacteria;Rhizobiales;unclassified_Rhizobiales;	Bacteria;Proteobacteria;Alphaproteobacteria;Rhizobiales;Rhizobiales Incertae Sedis;Phreatobacter;
ASV304	0.94054	91.3223	94	Bacteria;"Proteobacteria";Alphaproteobacteria;Caulobacterales;Hyphomonadaceae;unclassified_Hyphomonadaceae;	Bacteria;Proteobacteria;Alphaproteobacteria;Caulobacterales;Hyphomonadaceae;Hirschia;
ASV305	0.940708	90.4959	94	Bacteria;Cyanobacteria/Chloroplast;Cyanobacteria;unclassified_Cyanobacteria;	Bacteria;Cyanobacteria;Cyanobacteriia;Cyanobacteriales;Coleofasciculaceae;
ASV306	0	100	100	Bacteria;Firmicutes;Bacilli;Bacillales;Bacillaceae 1;Bacillus;	Bacteria;Firmicutes;Bacilli;Bacillales;Bacillaceae;Bacillus;
ASV307	0.985962	91.7355	98	Bacteria;"Planctomycetes";Planctomycetia;Planctomycetales;Planctomycetaceae;unclassified_Planctomycetaceae;	Bacteria;Planctomycetota;Planctomycetes;Gemmatales;Gemmataceae;uncultured;
ASV308	0	100	100	Bacteria;"Proteobacteria";Alphaproteobacteria;Sphingomonadales;Sphingomonadaceae;Sphingomonas;	Bacteria;Proteobacteria;Alphaproteobacteria;Sphingomonadales;Sphingomonadaceae;Sphingomonas;
ASV309	0	100	100	Bacteria;"Planctomycetes";Planctomycetia;Planctomycetales;Planctomycetaceae;unclassified_Planctomycetaceae;	Bacteria;Planctomycetota;Planctomycetes;Gemmatales;Gemmataceae;uncultured;

ASV310	0.994242	97.1193	99	Bacteria;"Proteobacteria";Betaproteobacteria;	Bacteria;Proteobacteria;Gammaproteobacteria;Burkholderiales;SC-I-84;
ASV311	0.994114	98.3539	99	Bacteria;"Verrucomicrobia";Verrucomicrobiae;Verrucomicrobiales;Verrucomicrobiaceae;	Bacteria;Verrucomicrobiota;Verrucomicrobiae;Verrucomicrobiales;Rubritaleaceae;Luteolibacter;
ASV312	0.990338	99.1736	99	Bacteria;"Proteobacteria";Alphaproteobacteria;Rhizobiales;	Bacteria;Proteobacteria;Alphaproteobacteria;Rhizobiales;Xanthobacteraceae;uncultured;
ASV314	0	100	100	Bacteria;"Actinobacteria";Actinobacteria;Actinobacteridae;Actinomycetales;Micrococcineae;Intrasporangiaceae;	Bacteria;Actinobacteriota;Actinobacteria;Micrococcales;Intrasporangiaceae;
ASV315	0	100	100	Bacteria;"Proteobacteria";Alphaproteobacteria;Caulobacterales;Caulobacteraceae;Brevundimonas;	Bacteria;Proteobacteria;Alphaproteobacteria;Caulobacterales;Caulobacteraceae; Brevundimonas;
ASV316	0	100	100	Bacteria;"Proteobacteria";Alphaproteobacteria;Rhodospirillales;Rhodospirillaceae;Skermanella;	Bacteria;Proteobacteria;Alphaproteobacteria;Azospirillales;Azospirillaceae;Skermanella;
ASV317	0	100	100	Bacteria;"Proteobacteria";Alphaproteobacteria;Sphingomonadales;Sphingomonadaceae; Novosphingobium;	Bacteria;Proteobacteria;Alphaproteobacteria;Sphingomonadales;Sphingomonadaceae;Novosphingobium;
ASV318	0.98742	93.4694	98	Bacteria;"Actinobacteria";Actinobacteria;Acidimicrobidae;Acidimicrobiales;"Acidimicrobineae";Iamiaceae;Aquihabitans;	Bacteria;Actinobacteriota;Acidimicrobiia;Microtrichales;Microtrichaceae;IMCC26207;
ASV319	0.994325	98.3471	99	Bacteria;"Proteobacteria";Alphaproteobacteria;Sphingomonadales;Sphingomonadaceae; Sphingomonas;	Bacteria;Proteobacteria;Alphaproteobacteria;Sphingomonadales;Sphingomonadaceae;Sphingomonas;
ASV320	0.991396	97.1193	99	Bacteria;"Chloroflexi";unclassified_"Chloroflexi";	Bacteria;Chloroflexi;KD4-96;
ASV321	0.994346	99.1736	99	Bacteria;"Proteobacteria";Alphaproteobacteria;Sphingomonadales;Sphingomonadaceae; Sphingomonas;	Bacteria;Proteobacteria;Alphaproteobacteria;Sphingomonadales;Sphingomonadaceae;Sphingomonas;
ASV322	0.991321	94.2857	99	Bacteria;"Actinobacteria";Actinobacteria;Acidimicrobidae;Acidimicrobiales;"Acidimicrobineae";Acidimicrobiaceae;Ilumatobacter;	Bacteria;Actinobacteriota;Acidimicrobiia;Microtrichales;Ilumatobacteraceae;CL500-29 marine group;
ASV323	0.98774	96.6942	98	Bacteria;"Proteobacteria";Betaproteobacteria;	Bacteria;Proteobacteria;Gammaproteobacteria;Burkholderiales;
ASV324	0.994418	99.1736	99	Bacteria;"Proteobacteria";Alphaproteobacteria;Rhizobiales;	Bacteria;Proteobacteria;Alphaproteobacteria;Rhizobiales;Beijerinckiaceae;
ASV325	0.994144	97.5207	99	Bacteria;"Planctomycetes";Planctomycetia;Planctomycetales;Planctomycetaceae;	Bacteria;Planctomycetota;Planctomycetes;Pirellulales;Pirellulaceae;Pirellula;
ASV326	0.98831	97.1074	98	Bacteria;"Bacteroidetes";Sphingobacteriia;"Sphingobacteriales";Chitinophagaceae;	Bacteria;Bacteroidota;Bacteroidia;Chitinophagales;Chitinophagaceae;uncultured;
ASV327	0.994434	98.7654	99	Bacteria;"Proteobacteria";Betaproteobacteria;Burkholderiales;	Bacteria;Proteobacteria;Gammaproteobacteria;Burkholderiales;Comamonadaceae;
ASV328	0.994413	99.5868	99	Bacteria;"Proteobacteria";Alphaproteobacteria;Caulobacterales;Caulobacteraceae;Phenylobacterium;	Bacteria;Proteobacteria;Alphaproteobacteria;Caulobacterales;Caulobacteraceae; Phenylobacterium;
ASV330	0.965264	88.843	96	Unclassified;	Bacteria; Proteobacteria;
ASV331	0.993865	98.7705	99	Bacteria;"Planctomycetes";Planctomycetia;Planctomycetales;Planctomycetaceae;Aquisphaera;	Bacteria;Planctomycetota;Planctomycetes;Isosphaerales;Isosphaeraceae;uncultured;
ASV332	0.994165	95.0617	99	Bacteria;"Actinobacteria";Actinobacteria;Acidimicrobidae;Acidimicrobiales;"Acidimicrobineae";	Bacteria;Actinobacteriota;Acidimicrobiia;Microtrichales;uncultured;

ASV333	0.99485	98.7603	99	Bacteria;"Proteobacteria";Gammaproteobacteria;Xanthomonadales;Sinobacteraceae;Povalibacter;	Bacteria;Proteobacteria;Gammaproteobacteria;Steroidobacterales;Steroidobacteraceae;uncultured;
ASV334	0.994469	99.5868	99	Bacteria;"Proteobacteria";Alphaproteobacteria;Rhizobiales;	Bacteria;Proteobacteria;Alphaproteobacteria;Rhizobiales;Rhizobiaceae;
ASV335	0	100	100	Bacteria;"Proteobacteria";Alphaproteobacteria;Rhizobiales;Methylocystaceae;Methylocystis;	Bacteria;Proteobacteria;Alphaproteobacteria;Rhizobiales;Beijerinckiaceae;Methylocystis;
ASV336	0.980468	92.1811	98	Bacteria;"Proteobacteria";Alphaproteobacteria;unclassified_Alphaproteobacteria;	Bacteria;Proteobacteria;Alphaproteobacteria;Tistrellales;Geminicoccaceae;Candidatus Alysiosphaera;
ASV337	0.990094	96.6942	99	Bacteria;Cyanobacteria/Chloroplast;Cyanobacteria;Family VIII;GpVIII;	Bacteria;Cyanobacteria;Cyanobacteriia;Cyanobacteriales;Xenococcaceae;Pleurocapsa PCC-7319;
ASV338	0	100	100	Bacteria;"Proteobacteria";Alphaproteobacteria;Caulobacterales;Caulobacteraceae;Brevundimonas;	Bacteria;Proteobacteria;Alphaproteobacteria;Caulobacterales;Caulobacteraceae;Brevundimonas;
ASV339	0.945606	91.7355	94	Bacteria;"Proteobacteria";Alphaproteobacteria;Caulobacterales;Hyphomonadaceae;	Bacteria;Proteobacteria;Alphaproteobacteria;Caulobacterales;Hyphomonadaceae;Hirschia;
ASV340	0.982025	98.3471	98	Bacteria;"Proteobacteria";Alphaproteobacteria;Rhodobacterales;Rhodobacteraceae;	Bacteria;Proteobacteria;Alphaproteobacteria;Rhodobacterales;Rhodobacteraceae;
ASV341	0.98011	93.8272	98	Bacteria;"Proteobacteria";Gammaproteobacteria;	Bacteria;Proteobacteria;Gammaproteobacteria;
ASV342	0	100	100	Bacteria;"Proteobacteria";Betaproteobacteria;Burkholderiales;Oxalobacteraceae;Massilia;	Bacteria;Proteobacteria;Gammaproteobacteria;Burkholderiales;Oxalobacteraceae;Massilia;
ASV343	0.99441	99.5868	99	Bacteria;"Actinobacteria";Actinobacteria;Actinobacteridae;Actinomycetales;Propionibacterineae;Nocardioidaceae;Nocardioides;	Bacteria;Actinobacteriota;Actinobacteria;Propionibacteriales;Nocardioidaceae;Nocardioides;
ASV344	0	100	100	Bacteria;"Proteobacteria";Gammaproteobacteria;"Enterobacteriales";Enterobacteriaceae;Plesiomonas;	Bacteria;Proteobacteria;Gammaproteobacteria;Enterobacterales;Enterobacteriaceae;Plesiomonas;
ASV345	0	100	100	Bacteria;"Proteobacteria";Alphaproteobacteria;Sphingomonadales;Sphingomonadaceae;Sphingomonas;	Bacteria;Proteobacteria;Alphaproteobacteria;Sphingomonadales;Sphingomonadaceae;Sphingomonas;
ASV346	0	100	100	Bacteria;"Actinobacteria";Actinobacteria;Actinobacteridae;Actinomycetales;Corynebacterineae;Mycobacteriaceae;Mycobacterium;	Bacteria;Actinobacteriota;Actinobacteria;Corynebacteriales;Mycobacteriaceae;Mycobacterium;
ASV347	0.975099	87.2428	97	Unclassified;	Bacteria; Bacteroidetes; Cytophagia; Cytophagales; Cytophagaceae; Rudanella;
ASV348	0.983379	95.8678	98	Bacteria;"Proteobacteria";Betaproteobacteria;unclassified_Betaproteobacteria;	Bacteria;Proteobacteria;Gammaproteobacteria;Burkholderiales;
ASV349	0.994362	97.9339	99	Bacteria;"Proteobacteria";Alphaproteobacteria;Rhizobiales;Hyphomicrobiaceae;	Bacteria;Proteobacteria;Alphaproteobacteria;Rhizobiales;Hyphomicrobiaceae;Hyphomicrobium;
ASV350	0.982072	98.3471	98	Bacteria;Cyanobacteria/Chloroplast;Cyanobacteria;	Bacteria;Cyanobacteria;Cyanobacteriia;Cyanobacteriales;Phormidiaceae;Tychonema CCAP 1459-11B;
ASV351	0	100	100	Bacteria;"Proteobacteria";Gammaproteobacteria;Pseudomonadales;Pseudomonadaceae;Azotobacter;	Bacteria;Proteobacteria;Gammaproteobacteria;Pseudomonadales;Pseudomonadaceae;Azotobacter;
ASV352	0	100	100	Bacteria;"Proteobacteria";Alphaproteobacteria;Sphingomonadales;Sphingomonadaceae;Sphingomonas;	Bacteria;Proteobacteria;Alphaproteobacteria;Sphingomonadales;Sphingomonadaceae;Sphingomonas;
ASV353	0.983319	90.5738	98	Bacteria;"Actinobacteria";Actinobacteria;Acidimicrobidae;Acidimicrobiales;"Acidimicrobineae";Iamiaceae;unclassified_Iamiaceae;	Bacteria;Actinobacteriota;Acidimicrobiia;Microtrichales;Ilumatobacteraceae;uncultured;

ASV354	0.989397	97.9424	98	Bacteria;"Proteobacteria";Alphaproteobacteria;Rhodobacterales;Rhodobacteraceae;unclassified_Rhodobacteraceae;	Bacteria;Proteobacteria;Alphaproteobacteria;Rhodobacterales;Rhodobacteraceae;
ASV355	0	100	100	Bacteria;"Actinobacteria";Actinobacteria;Actinobacteridae;Actinomycetales;Micrococcineae;Micrococcaceae;Micrococcus;	Bacteria;Actinobacteriota;Actinobacteria;Micrococcales;Micrococcaceae;Micrococcus;
ASV356	0.988083	98.7603	98	Bacteria;"Proteobacteria";Alphaproteobacteria;Rhodobacterales;Rhodobacteraceae;	Bacteria;Proteobacteria;Alphaproteobacteria;Rhodobacterales;Rhodobacteraceae;
ASV357	0.978281	90.535	97	Bacteria;"Gemmatimonadetes";Gemmatimonadetes;Gemmatimonadales;Gemmatimonadaceae;Gemmatimonas;	Bacteria;Gemmatimonadota;Gemmatimonadetes;Gemmatimonadales;Gemmatimonadaceae;Gemmatimonas;
ASV358	0	100	100	Bacteria;"Verrucomicrobia";Verrucomicrobiae;Verrucomicrobiales;Verrucomicrobiaceae;unclassified_Verrucomicrobiaceae;	Bacteria;Verrucomicrobiota;Verrucomicrobiae;Verrucomicrobiales;Verrucomicrobiaceae;uncultured;
ASV359	0	100	100	Bacteria;"Actinobacteria";Actinobacteria;Actinobacteridae;Actinomycetales;Propionibacterineae;Nocardioidaceae;Nocardioides;	Bacteria;Actinobacteriota;Actinobacteria;Propionibacteriales;Nocardioidaceae;Nocardioides;
ASV360	0.994148	95.0413	99	Bacteria;"Verrucomicrobia";Verrucomicrobiae;Verrucomicrobiales;Verrucomicrobiaceae;Luteolibacter;	Bacteria;Verrucomicrobiota;Verrucomicrobiae;Verrucomicrobiales;Rubritaleaceae;Luteolibacter;
ASV361	0	100	100	Bacteria;"Proteobacteria";Alphaproteobacteria;Sphingomonadales;Sphingomonadaceae;Sphingobium;	Bacteria;Proteobacteria;Alphaproteobacteria;Sphingomonadales;Sphingomonadaceae;Sphingobium;
ASV362	0.994354	99.1736	99	Bacteria;"Proteobacteria";Alphaproteobacteria;	Bacteria;Proteobacteria;Alphaproteobacteria;Rhizobiales;Pleomorphomonadaceae;Chthonobacter;
ASV363	0.994125	93.3884	99	Bacteria;Cyanobacteria/Chloroplast;Cyanobacteria;unclassified_Cyanobacteria;	Bacteria;Cyanobacteria;Cyanobacteriia;Cyanobacteriales;Coleofasciculaceae;Wilmottia Ant-Ph58;
ASV364	0.994247	97.1074	99	Bacteria;"Proteobacteria";Betaproteobacteria;Rhodocyclales;Rhodocyclaceae;	Bacteria;Proteobacteria;Gammaproteobacteria;Burkholderiales;SC-I-84;
ASV365	0.994247	97.5207	99	Bacteria;"Proteobacteria";Alphaproteobacteria;Rhizobiales;unclassified_Rhizobiales;	Bacteria;Proteobacteria;Alphaproteobacteria;Rhizobiales;Beijerinckiaceae;uncultured;
ASV366	0.983962	94.6502	98	Bacteria;"Proteobacteria";Alphaproteobacteria;Rhizobiales;	Bacteria;Proteobacteria;Alphaproteobacteria;Rhizobiales;
ASV367	0.994358	98.3471	99	Bacteria;"Proteobacteria";Alphaproteobacteria;Rhizobiales;Hyphomicrobiaceae;	Bacteria;Proteobacteria;Alphaproteobacteria;Rhizobiales;Hyphomicrobiaceae;
ASV368	0.979932	93.8017	97	Bacteria;"Planctomycetes";Planctomycetia;Planctomycetales;Planctomycetaceae;Pirellula;	Bacteria;Planctomycetota;Planctomycetes;Pirellulales;Pirellulaceae;Pirellula;
ASV369	0.994338	93.3884	99	Bacteria;Cyanobacteria/Chloroplast;Cyanobacteria;Family IV;GpIV;	Bacteria;Cyanobacteria;Cyanobacteriia;SepB-3;
ASV370	0.9943	99.1736	99	Bacteria;"Actinobacteria";Actinobacteria;Actinobacteridae;Actinomycetales;	Bacteria;Actinobacteriota;Actinobacteria;PeM15;
ASV371	0.987461	98.7603	98	Bacteria;"Planctomycetes";Planctomycetia;Planctomycetales;Planctomycetaceae;unclassified_Planctomycetaceae;	Bacteria;Planctomycetota;Planctomycetes;Pirellulales;Pirellulaceae;Pir4 lineage;
ASV372	0	100	100	Bacteria;"Proteobacteria";Betaproteobacteria;Burkholderiales;	Bacteria;Proteobacteria;Gammaproteobacteria;Burkholderiales;Comamonadaceae;
ASV373	0.994404	98.7603	99	Bacteria;"Proteobacteria";Betaproteobacteria;Burkholderiales;Burkholderiales_incertae_sedis;	Bacteria;Proteobacteria;Gammaproteobacteria;Burkholderiales;Comamonadaceae;
ASV374	0.994185	98.7603	99	Bacteria;"Planctomycetes";Planctomycetia;Planctomycetales;Planctomycetaceae;	Bacteria;Planctomycetota;Planctomycetes;Pirellulales;Pirellulaceae;Pirellula;

ASV375	0	100	100	Bacteria;"Proteobacteria";Alphaproteobacteria;Rhizobiales;Bradyrhizobiaceae;unclassified_Bradyrhizobiaceae;	Bacteria;Proteobacteria;Alphaproteobacteria;Rhizobiales;Xanthobacteraceae;uncultured;
ASV376	0	100	100	Bacteria;"Actinobacteria";Actinobacteria;Actinobacteridae;Actinomycetales;Micrococcineae;Microbacteriaceae;Agromyces;	Bacteria;Actinobacteriota;Actinobacteria;Micrococcales;Microbacteriaceae;Agromyces;
ASV377	0	100	100	Bacteria;Firmicutes;Bacilli;Bacillales;Bacillaceae 1;Bacillus;	Bacteria;Firmicutes;Bacilli;Bacillales;Bacillaceae;Bacillus;
ASV378	0.98793	97.9339	98	Bacteria;"Actinobacteria";Actinobacteria;Actinobacteridae;Actinomycetales;	Bacteria;Actinobacteriota;Actinobacteria;
ASV379	0.988101	99.1736	98	Bacteria;"Proteobacteria";Alphaproteobacteria;Rhodobacterales;Rhodobacteraceae;	Bacteria;Proteobacteria;Alphaproteobacteria;Rhodobacterales;Rhodobacteraceae;
ASV380	0	100	100	Bacteria;"Proteobacteria";Betaproteobacteria;Burkholderiales;Burkholderiales_incertae_sedis;	Bacteria;Proteobacteria;Gammaproteobacteria;Burkholderiales;Comamonadaceae;
ASV381	0.985448	97.9339	98	Bacteria;"Proteobacteria";Alphaproteobacteria;Sphingomonadales;Sphingomonadaceae;	Bacteria;Proteobacteria;Alphaproteobacteria;Sphingomonadales;Sphingomonadaceae;uncultured;
ASV382	0.994435	99.1736	99	Bacteria;"Proteobacteria";Alphaproteobacteria;Sphingomonadales;Erythrobacteraceae;	Bacteria;Proteobacteria;Alphaproteobacteria;Sphingomonadales;Sphingomonadaceae;
ASV383	0.994131	97.1074	99	Bacteria;"Verrucomicrobia";Verrucomicrobiae;Verrucomicrobiales;Verrucomicrobiaceae;unclassified_Verrucomicrobiaceae;	Bacteria;Verrucomicrobiota;Verrucomicrobiae;Verrucomicrobiales;DEV007;
ASV384	0	100	100	Bacteria;"Proteobacteria";Alphaproteobacteria;Rhizobiales;Methylobacteriaceae;Methylobacterium;	Bacteria;Proteobacteria;Alphaproteobacteria;Rhizobiales;Beijerinckiaceae;Methylobacterium-Methylorubrum;
ASV385	0	100	100	Bacteria;"Proteobacteria";Alphaproteobacteria;Sphingomonadales;Sphingomonadaceae;Sphingomonas;	Bacteria;Proteobacteria;Alphaproteobacteria;Sphingomonadales;Sphingomonadaceae;Sphingomonas;
ASV386	0.994264	100	99	Bacteria;"Proteobacteria";Deltaproteobacteria;unclassified_Deltaproteobacteria;	Bacteria;Myxococcota;bacteriap25;
ASV387	0.994372	99.5868	99	Bacteria;"Proteobacteria";Alphaproteobacteria;Caulobacterales;Hyphomonadaceae;Hyphomonas;	Bacteria;Proteobacteria;Alphaproteobacteria;Caulobacterales;Hyphomonadaceae;Hyphomonas;
ASV388	0	100	100	Bacteria;"Proteobacteria";Alphaproteobacteria;Rhodospirillales;Reyranella;	Bacteria;Proteobacteria;Alphaproteobacteria;Reyranellales;Reyranellaceae;Reyranella;
ASV391	0.988909	97.5309	98	Bacteria;"Chloroflexi";unclassified_"Chloroflexi";	Bacteria;Chloroflexi;KD4-96;
ASV392	0	100	100	Bacteria;"Planctomycetes";Planctomycetia;Planctomycetales;Planctomycetaceae;unclassified_Planctomycetaceae;	Bacteria;Planctomycetota;Planctomycetes;Planctomycetales;Rubinisphaeraceae;SH-PL14;
ASV393	0	100	100	Bacteria;"Proteobacteria";Alphaproteobacteria;Rhizobiales;	Bacteria;Proteobacteria;Alphaproteobacteria;Rhizobiales;Xanthobacteraceae;uncultured;
ASV394	0	100	100	Bacteria;"Proteobacteria";Betaproteobacteria;Burkholderiales;Comamonadaceae;Polaromonas;	Bacteria;Proteobacteria;Gammaproteobacteria;Burkholderiales;Comamonadaceae;Polaromonas;
ASV395	0	100	100	Bacteria;"Actinobacteria";Actinobacteria;Actinobacteridae;Actinomycetales;Corynebacterineae;Nocardiaceae;Gordonia;	Bacteria;Actinobacteriota;Actinobacteria;Corynebacteriales;Nocardiaceae;Gordonia;
ASV396	0.994456	99.5868	99	Bacteria;"Proteobacteria";Betaproteobacteria;Burkholderiales;	Bacteria;Proteobacteria;Gammaproteobacteria;Burkholderiales;Comamonadaceae;
ASV397	0.994445	99.5868	99	Bacteria;"Bacteroidetes";Flavobacteriia;"Flavobacteriales";Flavobacteriaceae;Flavobacterium;	Bacteria;Bacteroidota;Bacteroidia;Flavobacteriales;Flavobacteriaceae;Flavobacterium;
ASV398	0.994331	99.1736	99	Bacteria;"Proteobacteria";Gammaproteobacteria;Xanthomonadales;Xanthomonadaceae;Pseudoxanthomonas;	Bacteria;Proteobacteria;Gammaproteobacteria;Xanthomonadales;Xanthomonadaceae;Pseudoxanthomonas;

ASV399	0	100	100	Bacteria;"Actinobacteria";Actinobacteria;Actinobacteridae;Actinomycetales;Micrococcineae;Micrococcaceae;Arthrobacter;	Bacteria;Actinobacteriota;Actinobacteria;Micrococcales;Micrococcaceae;Glutamicibacter;
ASV400	0.994304	99.5868	99	Bacteria;"Acidobacteria";Acidobacteria_Gp4;Aridibacter;	Bacteria;Acidobacteriota;Blastocatellia;Blastocatellales;Blastocatellaceae;
ASV401	0.99415	97.1193	99	Bacteria;"Proteobacteria";Alphaproteobacteria;Rhodospirillales;Acetobacteraceae;Roseomonas;	Bacteria;Proteobacteria;Alphaproteobacteria;Acetobacterales;Acetobacteraceae;Roseomonas;
ASV402	0.991405	96.6942	99	Bacteria;"Proteobacteria";Alphaproteobacteria;Sphingomonadales;Sphingomonadaceae;	Bacteria;Proteobacteria;Alphaproteobacteria;Sphingomonadales;Sphingomonadaceae;
ASV403	0.994359	99.1736	99	Bacteria;"Proteobacteria";Alphaproteobacteria;Sphingomonadales;Sphingomonadaceae;Sphingomonas;	Bacteria;Proteobacteria;Alphaproteobacteria;Sphingomonadales;Sphingomonadaceae;Sphingomonas;
ASV404	0.98928	98.3471	98	Bacteria;"Acidobacteria";Acidobacteria_Gp6;Gp6;	Bacteria;Acidobacteriota;Vicinamibacteria;Vicinamibacterales;uncultured;
ASV405	0.994285	99.1736	99	Bacteria;"Proteobacteria";Gammaproteobacteria;Xanthomonadales;Xanthomonadaceae;Arenimonas;	Bacteria;Proteobacteria;Gammaproteobacteria;Xanthomonadales;Xanthomonadaceae;Arenimonas;
ASV406	0.991539	97.9339	99	Bacteria;"Proteobacteria";Betaproteobacteria;unclassified_Betaproteobacteria;	Bacteria;Proteobacteria;Gammaproteobacteria;Burkholderiales;SC-I-84;
ASV407	0	100	100	Bacteria;"Proteobacteria";Alphaproteobacteria;Sphingomonadales;Sphingomonadaceae;Sphingomonas;	Bacteria;Proteobacteria;Alphaproteobacteria;Sphingomonadales;Sphingomonadaceae;Sphingomonas;
ASV408	0.994399	99.5868	99	Bacteria;"Proteobacteria";Alphaproteobacteria;Rhizobiales;	Bacteria;Proteobacteria;Alphaproteobacteria;Rhizobiales;Rhizobiaceae;
ASV409	0	100	100	Bacteria;"Actinobacteria";Actinobacteria;Rubrobacteridae;Solirubrobacterales;Solirubrobacteraceae;Solirubrobacter;	Bacteria;Actinobacteriota;Thermoleophilia;Solirubrobacterales;Solirubrobacteraceae;Solirubrobacter;
ASV410	0.975783	97.9339	97	Bacteria;"Proteobacteria";Alphaproteobacteria;Rhizobiales;	Bacteria;Proteobacteria;Alphaproteobacteria;Rhizobiales;Rhizobiaceae;
ASV411	0.98868	98.3539	98	Bacteria;"Proteobacteria";Alphaproteobacteria;Rhizobiales;Hyphomicrobiaceae;Hyphomicrobium;	Bacteria;Proteobacteria;Alphaproteobacteria;Rhizobiales;Hyphomicrobiaceae;Hyphomicrobium;
ASV412	0.983231	89.2562	98	Bacteria;"Actinobacteria";Actinobacteria;Rubrobacteridae;	Bacteria;Actinobacteriota;Thermoleophilia;Gaiellales;uncultured;
ASV414	0.994449	99.1736	99	Bacteria;"Proteobacteria";Gammaproteobacteria;Pseudomonadales;Moraxellaceae;Acinetobacter;	Bacteria;Proteobacteria;Gammaproteobacteria;Pseudomonadales;Moraxellaceae;Acinetobacter;
ASV416	0.994316	97.5207	99	Bacteria;"Bacteroidetes";Sphingobacteriia;"Sphingobacteriales";Chitinophagaceae;	Bacteria;Bacteroidota;Bacteroidia;Chitinophagales;Chitinophagaceae;
ASV417	0.973772	90.4959	97	Bacteria;Cyanobacteria/Chloroplast;Chloroplast;Chloroplast;Bacillariophyta;	Bacteria;Cyanobacteria;Cyanobacteriia;Chloroplast;
ASV418	0.983924	96.6942	98	Bacteria;"Proteobacteria";Alphaproteobacteria;Sphingomonadales;	Bacteria;Proteobacteria;Alphaproteobacteria;Sphingomonadales;Sphingomonadaceae;Altererythrobacter;
ASV419	0.99437	99.1803	99	Bacteria;"Proteobacteria";Alphaproteobacteria;Rhizobiales;	Bacteria;Proteobacteria;Alphaproteobacteria;Rhizobiales;Beijerinckiaceae;
ASV420	0	100	100	Bacteria;"Proteobacteria";Gammaproteobacteria;Xanthomonadales;Xanthomonadaceae;	Bacteria;Proteobacteria;Gammaproteobacteria;Xanthomonadales;Xanthomonadaceae;
ASV421	0	100	100	Bacteria;"Actinobacteria";Actinobacteria;Actinobacteridae;Actinomycetales;Corynebacterineae;Dietziaceae;Dietzia;	Bacteria;Actinobacteriota;Actinobacteria;Corynebacteriales;Dietziaceae;Dietzia;
ASV427	0	100	100	Bacteria;"Acidobacteria";Acidobacteria_Gp4;Aridibacter;	Bacteria;Acidobacteriota;Blastocatellia;Blastocatellales;Blastocatellaceae;

ASV428	0	100	100	Bacteria;"Proteobacteria";Alphaproteobacteria;Rhizobiales;Beijerinckiaceae;Camelimonas;	Bacteria;Proteobacteria;Alphaproteobacteria;Rhizobiales;Beijerinckiaceae;Camelimonas;
ASV429	0.98815	97.9339	98	Bacteria;"Proteobacteria";Alphaproteobacteria;Sphingomonadales;Sphingomonadaceae;	Bacteria;Proteobacteria;Alphaproteobacteria;Sphingomonadales;Sphingomonadaceae;
ASV431	0	100	100	Bacteria;"Proteobacteria";Alphaproteobacteria;Sphingomonadales;Sphingomonadaceae; Sphingomonas;	Bacteria;Proteobacteria;Alphaproteobacteria;Sphingomonadales;Sphingomonadaceae;Sphingomonas;
ASV432	0.994437	99.1736	99	Bacteria;"Proteobacteria";Betaproteobacteria;Burkholderiales;Oxalobacteraceae;Noviherbaspirillum;	Bacteria;Proteobacteria;Gammaproteobacteria;Burkholderiales;Oxalobacteraceae;Noviherbaspirillum;
ASV434	0.98802	98.3471	98	Bacteria;"Proteobacteria";Alphaproteobacteria;Rhodobacterales;Rhodobacteraceae;	Bacteria;Proteobacteria;Alphaproteobacteria;Rhodobacterales;Rhodobacteraceae;
ASV437	0.987892	98.3471	98	Bacteria;"Proteobacteria";Alphaproteobacteria;Rhodobacterales;Rhodobacteraceae;	Bacteria;Proteobacteria;Alphaproteobacteria;Rhodobacterales;Rhodobacteraceae;
ASV439	0	100	100	Bacteria;"Proteobacteria";Gammaproteobacteria;Pseudomonadales;Moraxellaceae;Acinetobacter;	Bacteria;Proteobacteria;Gammaproteobacteria;Pseudomonadales;Moraxellaceae;Acinetobacter;
ASV440	0.963125	97.9339	96	Bacteria;"Proteobacteria";Alphaproteobacteria;Rhodobacterales;Rhodobacteraceae;unclassified_Rhodobacteraceae;	Bacteria;Proteobacteria;Alphaproteobacteria;Rhodobacterales;Rhodobacteraceae;
ASV441	0.994372	99.1736	99	Bacteria;"Proteobacteria";Gammaproteobacteria;Xanthomonadales;Xanthomonadaceae; Lysobacter;	Bacteria;Proteobacteria;Gammaproteobacteria;Xanthomonadales;Xanthomonadaceae;Lysobacter;
ASV442	0.960152	95.0413	96	Bacteria;"Proteobacteria";Alphaproteobacteria;Rhizobiales;Hyphomicrobiaceae;	Bacteria;Proteobacteria;Alphaproteobacteria;Rhizobiales;Hyphomicrobiaceae;Pedomicrobium;
ASV447	0	100	100	Bacteria;"Fusobacteria";Fusobacteriia;"Fusobacteriales";"Fusobacteriaceae";Cetobacterium;	Bacteria;Fusobacteriota;Fusobacteriia;Fusobacteriales;Fusobacteriaceae;Cetobacterium;
ASV450	0.978769	95.8678	97	Bacteria;"Proteobacteria";Betaproteobacteria;unclassified_Betaproteobacteria;	Bacteria;Proteobacteria;Gammaproteobacteria;Burkholderiales;Sutterellaceae;AAP99;
ASV451	0.977024	95.4545	97	Bacteria;"Proteobacteria";Betaproteobacteria;unclassified_Betaproteobacteria;	Bacteria;Proteobacteria;Gammaproteobacteria;Burkholderiales;Sutterellaceae;AAP99;
ASV463	0.994056	98.3471	99	Unclassified;	Bacteria;Firmicutes;Bacilli;Alicyclobacillales;Alicyclobacillaceae;Tumebacillus;
ASV467	0.99445	98.7603	99	Bacteria;"Bacteroidetes";Flavobacteriia;"Flavobacteriales";Flavobacteriaceae;Chryseobacterium;	Bacteria;Bacteroidota;Bacteroidia;Flavobacteriales;Weeksellaceae;Chryseobacterium;
ASV485	0.963499	97.9339	96	Bacteria;"Proteobacteria";Alphaproteobacteria;Rhizobiales;Brucellaceae;Ochrobactrum;	Bacteria;Proteobacteria;Alphaproteobacteria;Rhizobiales;
